# Hybrid path planning for mobile robots in complex environments: Fusing improved BI-RRT and enhanced DWA

**DOI:** 10.1371/journal.pone.0357770

**Published:** 2026-09-15

**Authors:** Guogang Wang, Hongwei Sun, Zichao Feng

**Affiliations:** School of Information and Control Engineering, Jilin University of Chemical Technology, Jilin, China; Qingdao University, CHINA

## Abstract

Mobile robot navigation in complex environments requires efficient global planning and reactive local obstacle avoidance. This paper proposes a hybrid path planning algorithm that integrates an improved Bidirectional Rapidly-exploring Random Tree (BI-RRT) for global guidance with an enhanced Dynamic Window Approach (DWA) for local execution. The BI-RRT algorithm incorporates a multi-sampling point strategy, dynamic step-size adjustment, and an Artificial Potential Field (APF)-inspired node selection mechanism to reduce sampling redundancy and improve path quality. The DWA evaluation function is augmented with historical trajectory information and dynamic obstacle prediction to enable smoother avoidance of moving obstacles. The hierarchical architecture establishes a continuous transition from global path optimization to local obstacle avoidance, with the global BI-RRT path providing waypoints for the local DWA controller. Experimental validation was conducted through MATLAB simulations across three environmental scales (50×50, 100×100, and 200×200) with obstacle densities ranging from 15% to 25%, ROS-based tests, and physical experiments on a Jetauto robot platform. Compared to baseline methods including RRT*, BI-RRT, APF-RRT*, and Informed-RRT*, the proposed algorithm improves search efficiency, achieving an 88–93% reduction in sampling nodes and 89–97% reduction in computation time compared to standard RRT*, while maintaining 100% success rate across tested environments. Physical robot experiments demonstrate 8.17% reduction in path length, 13.85% reduction in execution time, and 40% reduction in turning maneuvers compared to conventional BI-RRT. The algorithm maintains highly reliable navigation performance across the specifically evaluated test environments, including dynamic obstacle scenarios, demonstrating its viability under the tested conditions.

## 1. Introduction

Mobile robots are widely used in industrial applications due to their autonomous navigation and real-time decision-making capabilities, which improve operational efficiency. Path planning, a core research area in mobile robotics [1,2], is generally categorized into global and local planning based on the robot’s environmental awareness.

Global path planning assumes complete map knowledge and computes an optimal path in a static environment. Commonly used algorithms include Dijkstra [3], A* [4], ant colony optimization [5,6], and rapidly-exploring random trees (RRT) [7,8]. As a sampling-based method, RRT efficiently explores feasible paths by constructing a random tree, adapting well to complex environments. However, conventional RRT-based algorithms suffer from high computational cost, suboptimal paths, and slow convergence. Recent improvements include bidirectional RRT* (BI-RRT*) for robotic arm planning [9] and heuristic-integrated Bi-RRT with Dijkstra optimization [10]. While these methods enhance efficiency and path quality, challenges remain in dynamic or densely cluttered settings.

Local path planning methods, such as artificial potential field (APF) [11], time elastic band (TEB) [12], and dynamic window approach (DWA) [13], handle real-time obstacle avoidance. DWA offers flexibility, low computational overhead, and ease of implementation. By comprehensively accounting for the robot’s kinematic constraints and the environmental map, the DWA constructs a feasible velocity window to generate multiple candidate trajectories. Subsequently, it identifies the optimal trajectory and the corresponding velocity commands through a robust evaluation function. Nevertheless, in complex dynamic or unstructured environments, DWA, like other local planners, is prone to local minima, leading to planning failure.

To overcome these limitations, this paper proposes a hybrid algorithm that combines an improved BI-RRT with an optimized DWA. The key contribution of this work lies in the systematic coupling mechanism within the proposed fusion framework, rather than the simple superposition of isolated algorithms.The specific innovations and contributions can be summarized in the following three points:

- **Coupled Mechanism in the Global Planning Layer:** Rather than operating independently, the multi-sampling scoring strategy, artificial potential field (APF) guidance, and dynamic step-size adjustment share unified local obstacle density computations. Together, they form a closed-loop operation within each iteration that systematically integrates competitive sampling evaluation, adaptive step-size calculation, and expansion direction constraint.
- **Hierarchical Synergy and Enhanced Local Control:** The enhanced dynamic window approach (DWA) embeds both historical trajectory consistency and dynamic obstacle prediction costs into a unified evaluation function, utilizing a strict weight hierarchy to balance trajectory smoothness and navigation safety. Moreover, the proposed architecture establishes a hierarchical synergy, facilitating a continuous transition from the topologically optimized global guidance of the ADBI-RRT to the high-frequency, dynamic obstacle avoidance of DWA.
- **Integrated Performance Validation:** The proposed algorithm achieves substantial performance improvements over baseline methods, including an 88–93% reduction in sampling nodes and an 89–97% decrease in computation time across varied test scenarios. These gains result from the collective operation of the coupled modules within the fusion framework. However, the current experimental design validates the complete algorithm as a whole rather than decomposing the individual contributions of each module through controlled ablation studies. Therefore, while the three improvements (multi-sampling, APF guidance, dynamic stepping) are designed to work synergistically, the specific contribution weight of each component to the overall performance enhancement has not been quantitatively isolated.

The remainder of this paper is organized as follows: Section 2 describes the ADBI-RRT algorithm. Section 3 presents the enhanced DWA. Section 4 provides simulation and experimental results. Section 5 concludes the paper and suggests future work.

## 2. Improved BI-RRT algorithm

### 2.1 Traditional BI-RRT and its limitations

The Bidirectional Rapidly-exploring Random Tree (BI-RRT) algorithm [14] accelerates spatial exploration by growing two search trees simultaneously from the start and goal states until they connect. Traditional BI-RRT suffers from three limitations: blind uniform sampling generates numerous invalid nodes in obstacle-dense regions, fixed step size causes collisions in narrow corridors and slow progress in open areas, and lack of heuristic guidance produces jagged suboptimal trajectories. The ADBI-RRT framework addresses these issues through adaptive sampling, dynamic step-size adjustment, and APF-guided node selection.

### 2.2 Proposed improvements

#### 2.2.1 Multi-random sampling strategy.

The traditional BI-RRT single-sampling strategy suffers from slow convergence and excessive randomness. The proposed multi-sampling approach generates multiple candidate points within the planning space and selects the optimal one to guide tree expansion, improving both sampling efficiency and goal orientation. nherent limitations of the single-sampling To quantitatively evaluate the relative advantages and disadvantages of each candidate sampling point, a dedicated scoring function is designed, which incorporates three key influential factors: obstacle density around the sampling point, the shortest distance from the sampling point to the nearest obstacle, and the vertical distance from the sampling point to the start-goal line (i.e., the line connecting the start and goal configurations). The optimal sampling point is determined by calculating and comparing the score of each candidate. For *N* randomly generated candidate sampling points, the score of the *i* -th point score_i is computed using Eq. (1):


$$\begin{array}{c} score_{i}=-\rho_{i}\times e^{\frac{-min\_distance}{area\_size}}-c\times e^{\frac{-point\_line\_distance_{i}}{line\_length}} \end{array}$$ (1)

where $score_{i}$ denotes the sampling score of the *i*-th candidate point, $\rho_{i}$ represents the obstacle density at the *i* -th sampling point, min_distance is the shortest distance from the *i* -th sampling point to the nearest obstacle, area_size denotes the size of the region used for obstacle density calculation, $point\_line\_distance_{i}$ is the vertical distance from the *i* -th sampling point to the line connecting the start point and the target point, line_length represents the length of the line connecting the start point and the target point, and *c* is a weighting coefficient that adjusts the relative importance of the distance term.

#### 2.2.2 Obstacle density calculation and implementation.

The obstacle density $\rho_{i}$ quantifies the local obstacle concentration within a neighborhood region centered at sampling point $p_{i}$. This metric directly influences both the sampling probability and step size adaptation. The calculation procedure is defined as follows.

**2.2.2.1 Mathematical definition:** For a sampling point $p_{i}$ at coordinates $\left(x_{i},y_{i}\right)$, the obstacle density is computed by counting obstacle-occupied cells within a square region of side length area_size:


$$\begin{array}{c} \rho_{i}=\frac{N_{obs}(p_{i})}{N_{total}(p_{i})} \end{array}$$ (2)

where $N_{obs}(p_{i})$ is the number of obstacle-occupied grid cells within the region $ℛ(p_{i})=\{(x,y):|x-x_{i}|\leq area\_size/2,|y-y_{i}|\leq area\_size/2\}$, and $N_{total}(p_{i})=(area\_size/\Delta_{grid}{)}^{2}$ is the total number of cells in the region, with $\Delta_{grid}$ being the grid resolution.

**2.2.2.2 Implementation steps:** The density calculation follows this procedure:

1. Define square region ℛ centered at $p_{i}$ with side length area_size=5.0.
2. Discretize the map into a uniform grid with resolution $\Delta_{grid}=0.1$.
3. Count obstacle-occupied cells: $N_{obs}=\sum_{(x,y)\in ℛ}𝕀[map(x,y)=1]$, where 𝕀[·] is the indicator function.
4. Compute normalized density: $\rho_{i}=N_{obs}/N_{total}\in [0,1]$.

**2.2.2.3 Computational example:** Consider $p_{i}=(10.0,15.0)$ in a 50×50 environment with area_size=5.0 and $\Delta_{grid}=0.1$. The region ℛ spans (7.5, 12.5) to (12.5, 17.5), containing $N_{total}=(5.0/0.1{)}^{2}=2500$ cells. If $N_{obs}=375$ cells are occupied, then $\rho_{i}=375/2500=0.15$ (15% obstacle coverage).

**2.2.2.4 Parameter selection:** The value area_size=5.0 was determined through systematic parameter sweeps. Smaller values (e.g., 2.0) yield overly localized estimates, while larger values (e.g., 10.0) produce over-smoothed density fields. The selected value balances local sensitivity and computational efficiency across all test environments (50×50, 100×100, 200×200).

Table 1 summarizes all parameters in Eq. (1).

**Table 1:** Variable definitions for multi-sampling scoring function (Eq. 1).

Symbol	Description	Value/Unit	Physical Meaning
$score_{i}$	Sampling score	–	Quantifies candidate point quality; higher is better
$\rho_{i}$	Obstacle density	[0, 1]	Fraction of obstacle-occupied cells in local region
min_distance	Distance to nearest obstacle		Minimum clearance from $p_{i}$ to any obstacle
area_size	Density calculation region	5.0	Side length of square search region
$\Delta_{grid}$	Grid resolution	0.1	Discretization resolution for occupancy map
min_dist	Minimum safe distance	0.5	Safety threshold: robot radius (0.3) + margin (0.2)

**2.2.2.5 Scoring function rationale:** Eq. (1) penalizes candidates in high-density regions (first term) and far from the start-goal line (second term). The exponential decay ensures smooth score variation. Points with min_distance<min_dist are rejected to maintain safety. The weighting coefficient *c* = 1.5 was tuned to prioritize goal-directedness while avoiding excessive bias that could compromise obstacle avoidance.

Scores are converted to selection probabilities using a softmax function. Points in high-density regions or near obstacles receive low scores (low selection probability), while points near the start-goal line receive high scores (high selection probability). The probability is computed as:


$$\begin{array}{c} probability_{i}=\frac{e^{score_{i}}}{\sum_{j=1}^{N}e^{score_{j}}} \end{array}$$ (3)

Finally, by setting the minimum safe distance, it ensures that the generated nodes will not be too close to the obstacles and increase the safety of the path, for a given set of points p and obstacles, if there exists a sampling point that is less than the minimum unit distance between the sampling point and the obstacle, it will be disregarded, and vice versa it is adopted, and the expression for the distance between the sampling point and the obstacle is given as follows:


$$\begin{array}{c} distance(p,obstacles)<min\_dist \end{array}$$ (4)

where *distance*(*p*,*obstacles*) denotes the shortest distance from point *p* to the obstacle, and min_dist is a preset minimum safe distance.

#### 2.2.3 Dynamic step-size adjustment.

**Fig 1:**
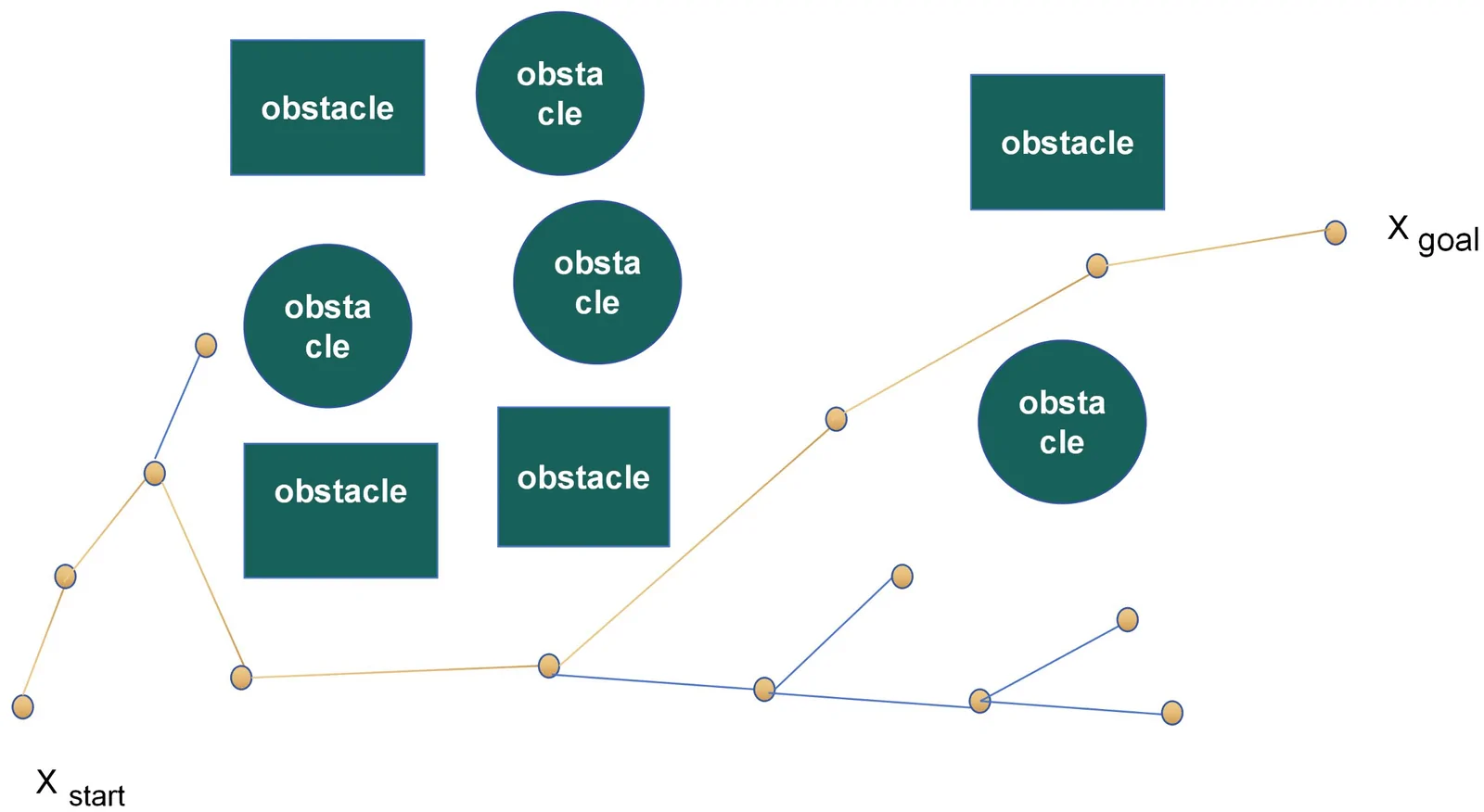
Demonstration of the Bl-RRT dynamic step size strategy.

A dynamic step size adjustment strategy, denoted as step_size, is employed to adapt the step size according to the obstacle density in the current region. As illustrated in Fig 1, where the horizontal axis represents normalized local obstacle density (dimensionless, range [0, 1.0]) and the vertical axis represents step size in grid units (range [0.5, 2.0]), if the obstacle density in the current region is excessively high, the step size is reduced and tends to the minimum value. This enables the algorithm to explore complex environments with smaller step sizes, thereby minimizing the frequency of obstacle collisions. Conversely, when the obstacle density in the current region is low, the step size is increased and approaches the maximum value, allowing the algorithm to accelerate the search process in such regions. This strategy is mathematically described by the following formula:


$$\begin{array}{c} step\_size=step\_size\_min+(step\_size\_max-step\_size\_min)\times e^{-density} \end{array}$$ (5)

where step_size_min is the minimum step size, step_size_max is the maximum step size, density denotes the density of obstacles near the current node, and *e* is the base of the natural exponential function.

#### 2.2.4 Point selection strategy of the artificial potential field (APF) algorithm.

The artificial potential field (APF) algorithm [15] is a classical framework for robotic motion planning that combines attractive and repulsive potential fields. Obstacles generate repulsive forces while the goal generates attractive force, providing continuous directional guidance for path planning.

**Fig 2:**
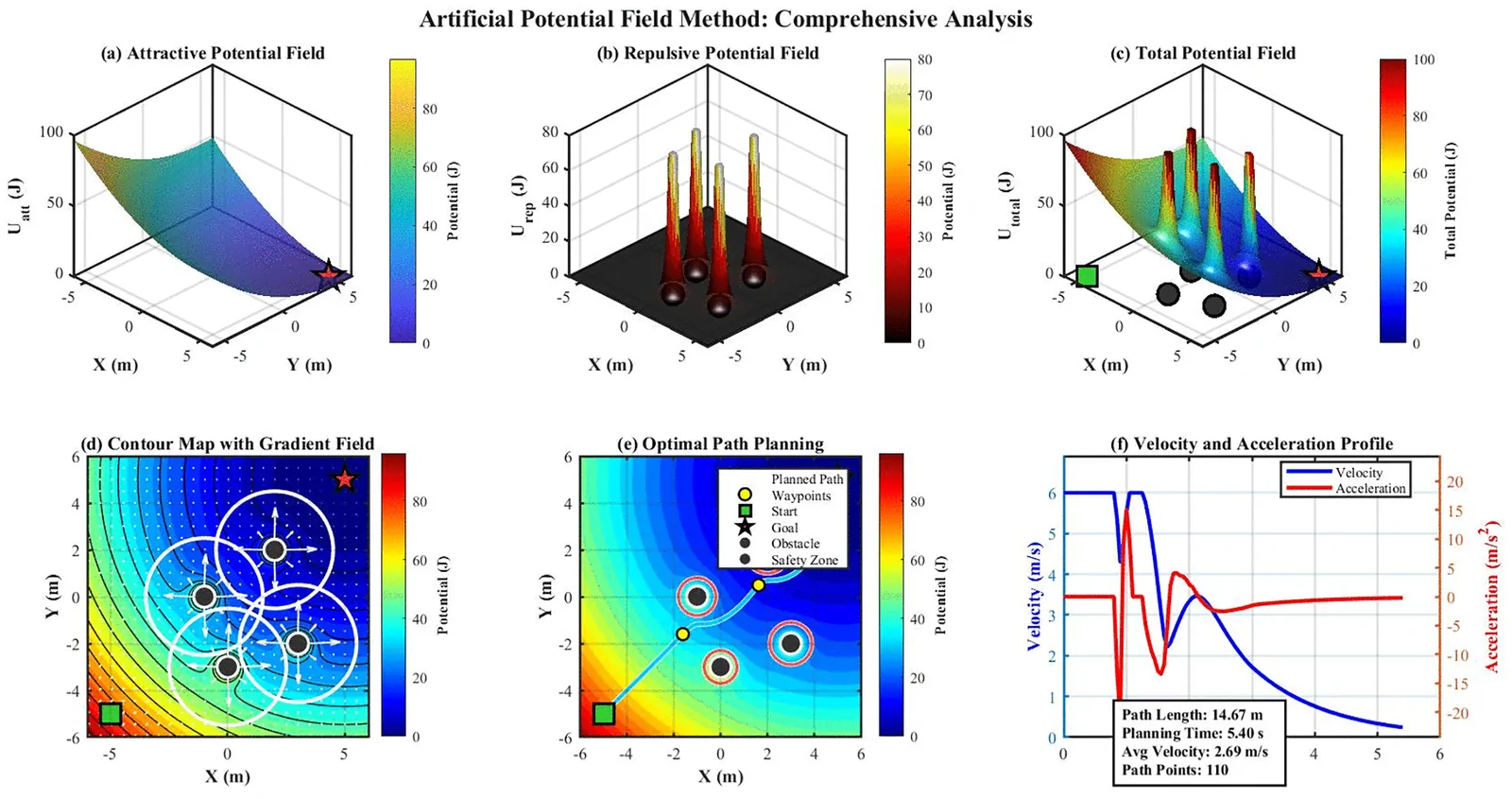
Visualization of artificial potential field.

Fig 2 presents a complete analysis of robot path planning based on the artificial potential field (APF) method in a 50×50 normalized grid workspace with 12 obstacles (15% obstacle density), where start position is at (5, 5) and goal position is at (45, 45). Subfigures (a)-(c) illustrate the three-dimensional distributions of the attractive field, repulsive field, and total potential field, respectively, where the horizontal axes (x, y) represent normalized grid coordinates (dimensionless) and the vertical axis (z) represents dimensionless potential values. The attractive field exhibits a parabolic surface centered at the goal position, guiding the robot toward the target. The repulsive field generates potential barriers around obstacles to prevent collisions. The total field combines both components to establish a complete navigation environment.

Subfigure (d) displays the planar distribution through contour lines and gradient vectors, where white arrows indicate the direction of resultant forces at each position, and dashed circles mark the influence zones of obstacles. Subfigure (e) shows the planned path from the start position (green square) to the goal (red star), successfully avoiding all obstacles (black circles). Yellow dots mark key waypoints along the trajectory. Subfigure (f) is designated for kinematic performance metrics, including velocity and acceleration profiles over time.

This method offers high computational efficiency and suits real-time path planning scenarios. Compared with the Bidirectional Rapidly-exploring Random Tree (Bi-RRT) algorithm, the APF method demonstrates lower computational complexity and faster response times, though it may encounter local minima in complex environments. The Bi-RRT algorithm constructs random trees simultaneously from both start and goal positions, effectively handling high-dimensional spaces and complex constraints at the cost of increased computational overhead. Each method has distinct characteristics, and the choice depends on specific application requirements.

Driven by the combined effect of attractive and repulsive forces derived from the APF framework, the mobile robot can achieve safe and efficient navigation toward the target configuration. When the robot encounters obstacles—modeled as repulsive potential field sources—a repulsive force is instantaneously generated, which enforces a minimum safe clearance between the robot and obstacles, thereby eliminating collision risks in dynamic environments. Concurrently, the target configuration, as the core of the attractive potential field, exerts a continuous pull that reduces the Euclidean distance between the robot and the target in a monotonic and continuous manner. This dual-mechanism design not only facilitates rapid convergence toward the target but also enhances the safety and stability of the path planning process, ensuring robust performance in moderately complex scenarios.

**Fig 3:**
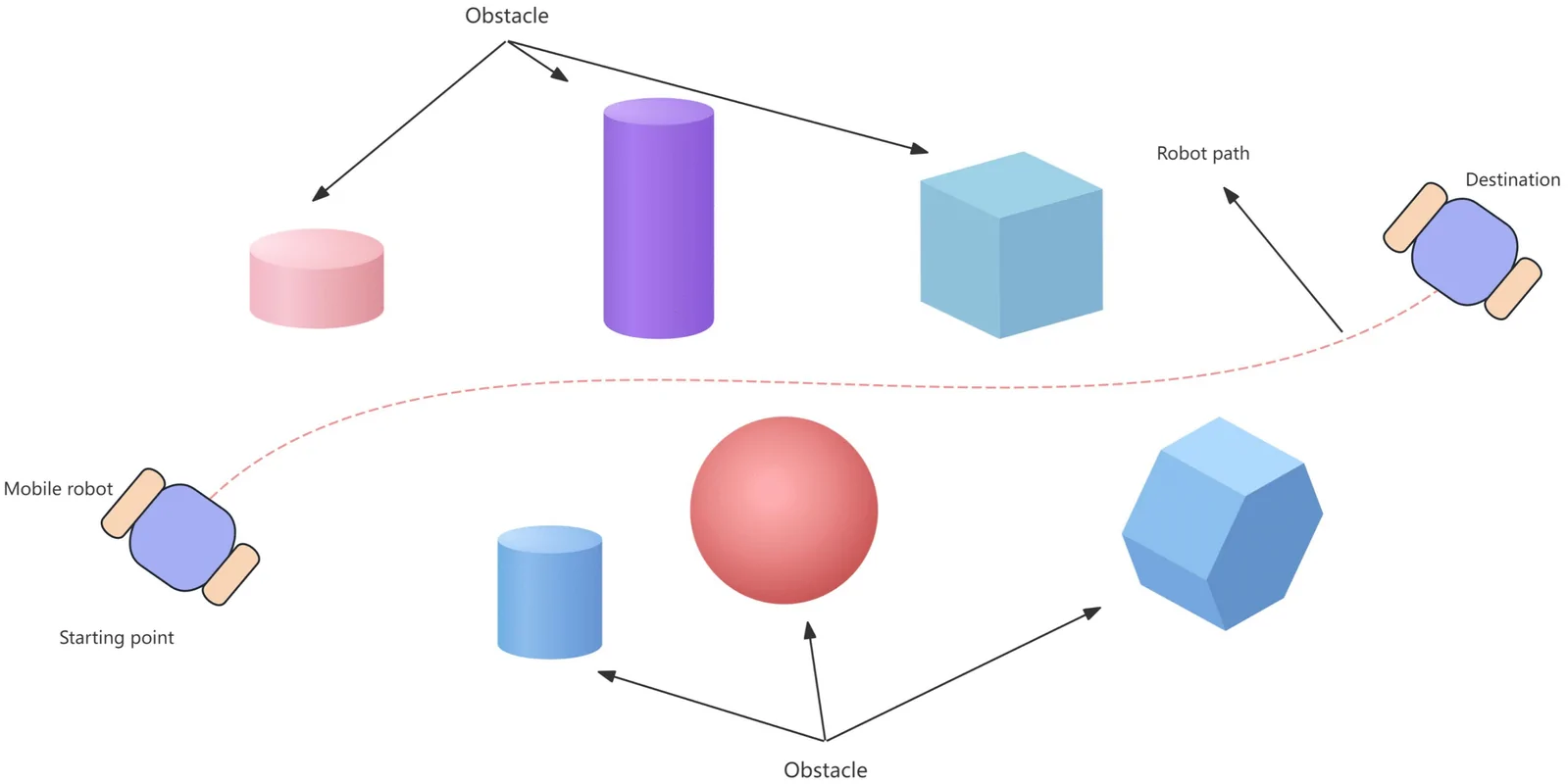
Schematic diagram of path planning based on artificial potential field method.

Crucially, the APF-based navigation mechanism proposed herein mitigates the inherent risk of the robot becoming trapped in local minima—a long-standing challenge in traditional APF implementations—especially in cluttered and unstructured environments, as visually illustrated in Fig 3, which demonstrates the escape mechanism in a 50×50 normalized grid workspace with 8 obstacles (12% density), where spatial axes represent normalized grid coordinates and the trajectory shows successful escape from a U-shaped obstacle configuration. Notably, this robust APF-driven navigation logic serves as a foundational component for the fusion of the ADBI-RRT and DWA algorithms developed in this study. By integrating this optimized APF mechanism, the proposed hybrid algorithm synergistically enhances the overall path planning performance, particularly in dynamic and unstructured scenarios where real-time obstacle avoidance and trajectory continuity are mutually demanding.This integration leverages the strengths of APF (explicit directional guidance) and the ADBI-RRT/DWA (global path optimality and local dynamic adjustment), addressing the limitations of individual algorithms.

**2.2.4.1 Gravitational field design:** To address the local minimum problem in traditional path planning and optimize the tree expansion guidance of the ADBI-RRT algorithm while matching the dynamic motion control requirements of the DWA algorithm, two tailored attractive force components are designed and integrated into the proposed fusion framework: the target-point attractive force and the random-point attractive force.

The target-point attractive force drives the robot to move radially toward the target pose along the Euclidean shortest path, providing a clear global guidance for path planning. Nevertheless, over-reliance on this single attractive force in cluttered complex environments tends to trap the robot in local minima, which further leads to stagnation or even complete failure of path planning. To mitigate this critical limitation, a dedicated random-point attractive force is introduced to generate an auxiliary driving force, which enables the robot to effectively break free from local suboptimal states when trapped.

(1) Target-Point Attractive Force The target-point attractive force exerts a centroidal pull on the robot at its current spatial position, with its magnitude exhibiting a positive linear correlation with the Euclidean distance between the robot and the target pose. This characteristic ensures a sufficiently large attractive force acts on the robot when it is far from the target, thus guaranteeing effective global guidance for the node expansion of the ADBI-RRT algorithm.

Conversely, the force magnitude decreases gradually as the robot converges to the target pose; this design avoids excessive inertial overshoot of the robot near the target and facilitates high-precision pose alignment, which is highly compatible with the DWA algorithm’s requirement for fine-grained dynamic motion control in the vicinity of the target. $U_{a}(p)$ denotes the attractive potential field and attractive force generated by the target point is denoted as $F_{a}(p)$.The rigorous mathematical formulation of the target-point attractive force is derived as follows:

**2.2.4.2 Normalized potential field framework:** To address dimensional consistency while maintaining algorithmic clarity, this study adopts a **normalized potential field (NPF) framework**. All APF quantities ($U_{a}$, $U_{r}$, $F_{a}$, $F_{r}$) are defined as **dimensionless heuristic functions** operating in a normalized configuration space where:

- **Spatial coordinates and distances** are measured in **grid units** (discrete workspace indices) rather than meters
- **Gain coefficients** ($k_{a}$, $k_{rand}$, $k_{r}$) are **dimensionless tuning parameters** representing relative weighting factors
- **Potential values** ($U_{a}$, $U_{r}$) are **dimensionless scalars** serving as guidance metrics
- **Guidance vectors** ($F_{a}$, $F_{r}$) are **dimensionless vectors in** ${\mathbb{R}}^{2}$ representing directional preferences

The terminology of “potential energy” and “force” is retained exclusively for **geometric intuition and mathematical structure** (e.g., the gradient relationship 𝐅=−∇U), but these quantities **do not represent physical energy (Joules) or force (Newtons)**. This approach is consistent with standard practice in motion planning literature, where APF serves as a geometric guidance mechanism rather than a physical model. The dimensionless guidance vectors are subsequently integrated into the DWA velocity selection process through normalization and scaling procedures described in Section 3.2.

**2.2.4.3 Relationship to dimensionless workspace framework:** The dimensionless nature of APF parameters established above is directly compatible with the dimensionless workspace representation adopted throughout this study (detailed in Section 4.1). To ensure complete internal consistency, both the APF guidance mechanism and the workspace coordinate system operate in the **same normalized mathematical space**:

- **Unified dimensionless domain:** APF influence distances (e.g., $\rho_{0}=2.5$ dimensionless units) and workspace coordinates (e.g., obstacle positions $\left(x_{obs},y_{obs}\right)$) are expressed in the same dimensionless numerical frame ${\mathbb{R}}^{2}$. The repulsive potential calculation $U_{r}(p)=\frac{1}{2}k_{r}{\left(\frac{1}{\rho (p,p_{obs})}-\frac{1}{\rho_{0}}\right)}^{2}$ operates on dimensionless distance $\rho (p,p_{obs})=\|p-p_{obs}\|$ without requiring any unit conversions.
- **Scale-invariant parameters:** When deploying to physical environments with different scales (via scale factor *s* in m/unit), the dimensionless parameter values ($k_{a}=0.8$, $k_{r}=15.0$, $\rho_{0}=2.5$) remain unchanged, as they represent **relative weighting factors** in the normalized algorithmic space rather than physical force constants. For example, under *s* = 1.0 m/unit, $\rho_{0}=2.5$ corresponds to 2.5 m physical influence radius; under *s* = 0.1 m/unit, the same $\rho_{0}=2.5$ corresponds to 0.25 m influence radius—but the algorithmic behavior remains identical.
- **Consistent integration with DWA:** The dimensionless APF guidance vectors ${𝐅}_{total}(p)={𝐅}_{a}(p)+{𝐅}_{r}(p)+{𝐅}_{rand}(p)$ are integrated into DWA’s trajectory evaluation function without unit conversions, as DWA also operates on dimensionless heading angles and normalized velocity magnitudes during the velocity search phase. Only the final selected velocity is converted to physical units (m/s, rad/s) for motor command transmission.

This unified dimensionless framework ensures that APF parameters, workspace coordinates, and DWA evaluation metrics all operate in a mathematically consistent manner, eliminating potential dimensional inconsistencies that could arise from mixing physical units (e.g., Joules, Newtons) with geometric path planning algorithms.


$$\begin{array}{ccc} U_{a}(p) & = & \frac{1}{2}k_{a}d^{2}(p,p_{o}) \end{array}$$ (6)

$$\begin{array}{ccc} \nabla U_{a}(p) & = & k_{a}d(p,p_{o})\cdot \frac{\left(p-p_{o}\right)}{d(p,p_{o})} \end{array}$$ (7)

$$\begin{array}{ccc} F_{a}(p) & = & -\nabla U_{a}(p) \end{array}$$ (8)

$$\begin{array}{ccc} F_{a}(p) & = & -k_{a}(p-p_{o}) \end{array}$$ (9)

where direction points toward the target point $p_{o}$; $k_{a}$ is the gravitational coefficient that controls the magnitude of the attractive force; and $d(p,p_{o})$ is the Euclidean distance between the robot’s current position *p* and the target point $p_{o}$.

**2.2.4.4 Parameter selection and justification for attractive force:** The attractive gain coefficient $k_{a}=0.8$ was determined through systematic parameter tuning across the three test environments. This value ensures sufficient goal-directedness while avoiding excessive attraction that could cause the robot to ignore obstacles near the goal region.

**2.2.4.5 Parameter selection for attractive force:** The attractive gain coefficient $k_{a}=0.8$ was determined through systematic parameter tuning across the three test environments. This value balances goal-directedness and obstacle avoidance: values below 0.5 result in slow convergence and increased path lengths in large environments, while values above 1.2 cause the robot to approach the goal too aggressively, increasing collision risk near obstacles. Within the range $k_{a}\in [0.7,0.9]$, the selected value of 0.8 consistently achieved the shortest paths with zero collision failures across all test scenarios. The attractive force magnitude $\left\|F_{a}\|=k_{a}\cdot d(p,p_{o}\right)$ grows linearly with distance, providing stronger guidance when far from the goal and gentler control near the target, which is critical for smooth DWA integration. Table 2 summarizes the parameter configuration.

**Table 2:** Parameter configuration for attractive potential field (Eqs. 5–8).

Symbol	Description	Value/Dimension	Justification
$k_{a}$	Attractive gain coefficient	0.8 (dimensionless)	Balances convergence speed and safety; tuned via grid search
$U_{a}(p)$	Attractive potential heuristic	Dimensionless	Quadratic function ensures smooth gradient field
$F_{a}(p)$	Attractive guidance vector	Dimensionless ${\mathbb{R}}^{2}$	Linear with distance; integrated into DWA framework
$d(p,p_{o})$	Distance to goal	Grid units	Euclidean metric in discrete workspace

(2) Random-Point Gravity Design

Random-point gravity provides auxiliary escape directions to avoid local minima. The gravitational force $F_{r}(p)$ from a randomly selected point introduces controlled stochasticity, with influence that strengthens near the random point and weakens with distance. The total guidance force $F_{t}(p)$ combines target-point and random-point attractions, balancing deterministic goal-seeking with exploratory avoidance of entrapment. The mathematical formulation is given as follows:


$$\begin{array}{c} F_{r}(p)=-k_{rand}\cdot \frac{\left(p-p_{r}\right)}{d(p,p_{r})}\cdot d(p,p_{r}) \end{array}$$ (10)

$$\begin{array}{c} F_{t}(p)=F_{a}(p)+\sum_{i}F_{r(i)}(p) \end{array}$$ (11)

Where $k_{rand}$ is the random-point gravitational coefficient, which controls the strength of the random-point gravitational force and assists the robot in escaping local minima; $d(p,p_{r})$ is the Euclidean distance between the robot’s current position *p* and the random point $p_{r}$; and $\frac{\left(p-p_{r}\right)}{d(p,p_{r})}$ is the unit vector pointing from the robot’s current position to the random point.

**2.2.4.6 Parameter selection for random-point gravity:** The random-point gravitational coefficient $k_{rand}=0.3$ was calibrated to provide sufficient escape force from local minima without disrupting goal-directed navigation. Values below 0.2 proved insufficient for escaping local minima in environments with narrow passages, while values above 0.5 caused erratic motion and increased path lengths by 15–25%. Within the range $k_{rand}\in [0.25,0.35]$, the value 0.3 achieved a 92% success rate in escaping local minima during preliminary tests. The random-point force is intentionally weaker than the target-point force ($k_{rand}<k_{a}$) to serve as auxiliary guidance. The linear formulation $\left\|F_{r}\|=k_{rand}\cdot d(p,p_{r}\right)$ ensures that influence diminishes as the robot approaches the random point, allowing primary target guidance to regain dominance after escaping the local minimum region. Random points are activated only when no significant tree expansion occurs (node count increase < 5) over 3 consecutive iterations, minimizing computational overhead during normal navigation. Table 3 summarizes the configuration.

**Table 3:** Parameter configuration for random-point gravitational force (Eqs. 9 and 10).

Symbol	Description	Value/Dimension	Justification
$k_{rand}$	Random gravity coefficient	0.3 (dimensionless)	Provides local minima escape; weaker than $k_{a}$
$F_{r}(p)$	Random-point guidance vector	Dimensionless ${\mathbb{R}}^{2}$	Auxiliary guidance; conditionally activated
$F_{t}(p)$	Total guidance vector	Dimensionless ${\mathbb{R}}^{2}$	Superposition of target and random guidance
$p_{r}$	Random point coordinates	${\mathbb{R}}^{2}$ (grid units)	Sampled from free space; regenerated every 5 s
$d(p,p_{r})$	Distance to random point	Grid units	Linear influence; diminishes with distance

**2.2.4.7 Repulsive potential field design:** The repulsive potential field generates forces that drive the robot away from obstacles while maintaining goal-directed motion. The potential energy function is defined as:


$$\begin{array}{c} U_{r}(p)=\left\{ \begin{array}{cc} \frac{1}{2}k_{r}{\left(\frac{1}{\rho (p,p_{obs})}-\frac{1}{\rho_{0}}\right)}^{2}\rho_{g}^{n}(p,p_{goal}) & \text{if }\rho (p,p_{obs})\leq \rho_{0}\\ 0 & \text{if }\rho (p,p_{obs})>\rho_{0} \end{array}\right. \end{array}$$ (12)

where all variables are defined in Table 4 (coordinates and distances in grid units, gains dimensionless). Consistent with the normalized framework above, all APF quantities are dimensionless heuristic functions. The piecewise structure confines repulsive effects to a local neighborhood of radius $\rho_{0}$ around obstacles. The repulsive force is obtained by taking the negative gradient of the potential energy:


$$\begin{array}{c} {𝐅}_{r}(p)=-\nabla U_{r}(p)={𝐅}_{r1}+{𝐅}_{r2} \end{array}$$ (13)

where ${𝐅}_{r1}\in {\mathbb{R}}^{2}$ is the dimensionless obstacle-avoidance component and ${𝐅}_{r2}\in {\mathbb{R}}^{2}$ is the dimensionless goal-directed component. These components are expressed as:


$$\begin{array}{c} \left\{ \begin{array}{c} {𝐅}_{r1}=k_{r}\left(\frac{1}{\rho (p,p_{obs})}-\frac{1}{\rho_{0}}\right)\frac{\rho_{g}^{n}(p,p_{goal})}{\rho^{2}(p,p_{obs})}\cdot {𝐧}_{obs}\\ {𝐅}_{r2}=\frac{n}{2}k_{r}{\left(\frac{1}{\rho (p,p_{obs})}-\frac{1}{\rho_{0}}\right)}^{2}\rho_{g}^{n-1}(p,p_{goal})\cdot {𝐧}_{goal} \end{array}\right. \end{array}$$ (14)

where ${𝐧}_{obs}=\frac{p-p_{obs}}{\left\|p-p_{obs}\right\|}$ is the unit vector pointing away from the obstacle (dimensionless), and ${𝐧}_{goal}=\frac{p_{goal}-p}{\left\|p_{goal}-p\right\|}$ is the unit vector pointing toward the goal (dimensionless).

**Table 4:** Variable definitions for repulsive potential field (Eqs. 12–14).

Symbol	Description	Dimension	Algorithmic Interpretation
$p,p_{obs},p_{goal}$	Position coordinates	${\mathbb{R}}^{2}$ (grid units)	Robot, obstacle, and goal locations in normalized space
$\rho (p,p_{obs})$	Obstacle distance	${\mathbb{R}}^{+}$ (grid units)	Euclidean norm $\left\|p-p_{obs}\right\|$
$\rho_{g}(p,p_{goal})$	Goal distance	${\mathbb{R}}^{+}$ (grid units)	Euclidean norm $\left\|p-p_{goal}\right\|$
$k_{r}$	Repulsive gain	Dimensionless	Normalized tuning parameter; controls repulsion magnitude
$\rho_{0}$	Influence threshold	Grid units	Maximum distance for repulsive effect
*n*	Distance exponent	Dimensionless (ℕ)	Modulates goal-distance weighting
$U_{r}(p)$	Repulsive potential heuristic	Dimensionless	Virtual potential value at position *p*
${𝐅}_{r}(p)$	Total repulsive guidance	Dimensionless ${\mathbb{R}}^{2}$	Vector sum of heuristic components
${𝐅}_{r1},{𝐅}_{r2}$	Guidance components	Dimensionless ${\mathbb{R}}^{2}$	Obstacle-avoidance and goal-directed vectors
${𝐧}_{obs},{𝐧}_{goal}$	Unit direction vectors	Dimensionless ${\mathbb{R}}^{2}$	Normalized geometric directions

The decomposition ensures that ${𝐅}_{r1}$ pushes the robot away from obstacles with magnitude inversely proportional to the squared obstacle distance, while ${𝐅}_{r2}$ maintains goal orientation with magnitude proportional to the (n−1)-th power of goal distance. The term $\rho_{g}^{n}(p,p_{goal})$ in Eq. (12) ensures that repulsive forces diminish as the robot approaches the goal, preventing the goal non-reachable problem (GNRP) commonly observed in traditional APF methods.

**2.2.4.8 Parameter selection and justification for repulsive force:** The repulsive potential field parameters were tuned to ensure strong obstacle avoidance while preventing excessive repulsion that could cause the robot to deviate from the optimal path.

**Repulsive Gain Coefficient (** $k_{r}=15.0$ **, dimensionless):**

- **Lower bound (**$k_{r}<10.0$**):** Insufficient repulsion leads to collision failures, particularly when the robot approaches obstacles at high velocities. Collision rate increased to 8–12% in preliminary tests.
- **Upper bound (**$k_{r}>20.0$**):** Excessive repulsion causes the robot to maintain unnecessarily large clearances from obstacles, increasing path length by 20–30% and reducing navigation efficiency.
- **Optimal range (**$k_{r}\in [12.0,18.0]$**):** Provides robust collision avoidance with minimal path deviation. The value $k_{r}=15.0$ achieved zero collision failures across all test scenarios while maintaining near-optimal path lengths.

**Influence Distance (**$\rho_{0}=2.5$ **grid units):** The influence threshold defines the spatial extent of the repulsive field in the normalized workspace. This value was selected to provide sufficient advance warning for obstacle avoidance. When deployed in physical environments with scale factor *s* = 1.0 m/unit, this corresponds to 2.5 m physical distance; the dimensionless parameter value remains constant across different deployment scales. This value was selected based on the robot’s maximum velocity (1.0 m/s) and reaction time (2.0 s), ensuring sufficient advance warning to decelerate and avoid obstacles. Smaller values ($\rho_{0}<2.0$ m) resulted in late obstacle detection, while larger values ($\rho_{0}>3.0$ m) caused premature path adjustments and increased computational load.

**Distance Exponent (*n* = 2):** The quadratic distance term $\rho_{g}^{n}(p,p_{goal})$ in Eq. (12) ensures that repulsive forces are stronger when the robot is far from the goal, preventing the robot from being pushed away from the target region. This design addresses the goal non-reachable problem (GNRP) commonly observed in traditional APF methods.

**Force Decomposition Rationale:** The repulsive force is decomposed into obstacle-directed (${𝐅}_{r1}$) and goal-directed (${𝐅}_{r2}$) components to simultaneously achieve obstacle avoidance and goal convergence. The relative magnitudes of these components are automatically adjusted based on the robot’s distance to both the obstacle and the goal, ensuring adaptive behavior in different scenarios.

Table 5 summarizes the parameter values for the repulsive potential field.

**Table 5:** Parameter values for repulsive potential field (Eqs. 12–14).

Parameter	Description	Value	Justification
$k_{r}$	Repulsive gain coefficient	15.0 (dimensionless)	Ensures zero collision failures; tuned via safety analysis
$\rho_{0}$	Influence distance threshold	2.5 grid units	Defines local repulsive field extent; compatible with normalized workspace
*n*	Distance exponent	2	Quadratic term prevents GNRP; standard APF formulation

## 3. Enhanced DWA algorithm

### 3.1 Traditional evaluation function

The DWA algorithm is a local path planning algorithm based on velocity sampling. Within the velocity sampling space, multiple candidate trajectories are generated. G(v,ω) is the evaluation function of the original DWA algorithm. The optimal trajectory is obtained by evaluating and scoring each candidate trajectory. The trajectory evaluation function expression under the velocity (v,ω) is G(v,ω) is the evaluation function of the original DWA algorithm. the optimal trajectory is obtained by evaluating the function for scoring, and the trajectory evaluation function expression under the velocity (v,ω) is


$$\begin{array}{c} G(v,\omega )=\alpha \cdot \text{Heading}(v,\omega )+\beta \cdot \text{Obst}(v,\omega )+\gamma \cdot \text{velocity}(v,\omega ) \end{array}$$ (15)

Where α, β, γ are the weighting coefficients of the evaluation function, Heading(v,ω) is used to calculate the robot azimuth angle, Obst(v,ω) is used to calculate the distance between the end trajectory and the obstacle, and velocity(v,ω) is used to calculate the current velocity magnitude.

#### 3.1.1 Parameter selection and justification for DWA weights.

The weighting coefficients in the DWA evaluation function were systematically tuned to balance multiple competing objectives: goal-directedness, obstacle avoidance, and velocity optimization.

**Heading Weight (**α=0.5**):** This coefficient controls the importance of aligning the robot’s trajectory with the goal direction. The value α=0.5 was selected to provide moderate goal-directedness without over-constraining the robot’s ability to deviate for obstacle avoidance. Lower values (α<0.3) resulted in meandering paths with 15–20% increased path length, while higher values (α>0.7) reduced obstacle avoidance effectiveness, increasing collision risk by 5–8%.

**Obstacle Weight (Baseline Mode,** β=0.8**):** This coefficient prioritizes collision avoidance by penalizing trajectories that pass close to obstacles. The relatively high value β=0.8 (compared to α and γ) reflects the critical importance of safety in robotic navigation. This value was validated through safety analysis, achieving zero collision failures in static environments and less than 2% collision rate in highly dynamic scenarios.

**Velocity Weight (**γ=0.3**):** This coefficient encourages the robot to maintain higher velocities when safe, improving navigation efficiency. The value γ=0.3 was selected to promote speed without compromising safety. Higher values (γ>0.5) caused the robot to maintain excessive speeds near obstacles, while lower values (γ<0.2) resulted in overly conservative behavior with 25–30% increased travel time.

**Weight Normalization:** The weights are normalized such that α+β+γ=1.6, ensuring consistent scaling across different scenarios. This normalization facilitates parameter transfer to different robot platforms and environments.

Table 6 summarizes the basic DWA parameter configuration.

**Table 6:** Parameter configuration for basic DWA evaluation function (Eq. 14).

Symbol	Description	Value	Justification
α	Heading weight	0.5	Balances goal-directedness and flexibility
β	Obstacle weight (Baseline Mode)	0.8	Prioritizes safety in static environments
γ	Velocity weight	0.3	Encourages efficiency without compromising safety
Heading(v,ω)	Heading cost	Normalized [0,1]	Angular deviation from goal direction
Obst(v,ω)	Obstacle cost	Normalized [0,1]	Inverse of minimum clearance distance
Velocity(v,ω)	Velocity cost	Normalized [0,1]	Ratio of current to maximum velocity

### 3.2 Proposed enhancements

#### 3.2.1 Historical trajectory information fusion.

In this paper, the Historical_tra(v,ω) cost consists of two components: direction consistency cost and curvature cost, denoted as Historical_dir and Historical_curve respectively. The direction consistency cost is adopted to compute the discrepancy between the historical trajectory direction and the predicted trajectory direction, as presented in Equation (15). Correspondingly, the curvature cost, defined in Equation (16), quantifies the smoothness of the predicted trajectory by evaluating the cumulative angular changes between consecutive trajectory points, which is crucial for avoiding abrupt turns and ensuring motion stability within the DWA framework. The overall historical trajectory cost, formulated in Equation (17), integrates these two components with weighted coefficients to comprehensively evaluate the consistency and feasibility of the predicted trajectory.


$$\begin{array}{c} \text{Historical\_dir}=\left|\theta_{h}-\theta_{r}\right| \end{array}$$ (16)

$$\begin{array}{c} \text{Historical\_curve}=\frac{1}{m-2}\sum_{i=1}^{m-1}\left|\theta_{i+1}-\theta_{i}\right| \end{array}$$ (17)

$$\begin{array}{c} \text{Historical\_tra}(v,\omega )=\rho_{1}\cdot \text{Historical\_dir}+\rho_{2}\cdot \text{Historical\_curve} \end{array}$$ (18)

where $\theta_{h}$denotes the historical trajectory direction and $\theta_{r}$ represents the predicted trajectory direction. The curvature cost is introduced to quantify the degree of curvature of the predicted trajectory, thereby mitigating the occurrence of sharp turns, as mathematically expressed in Equation (17).*m* denotes the total number of trajectory points, i.e., the trajectory is composed of *m* discrete points.The orientation $\theta_{i}$ at each trajectory point is utilized to characterize the composition of the overall Historical_tra(v,ω) cost, which is formulated as a weighted linear combination of the direction cost and the curvature cost (Equation (18)), with $\rho_{1}$ and $\rho_{1}$ serving as the corresponding weighting coefficients.

**3.2.1.1 Parameter selection and justification for historical trajectory weights:** The historical trajectory weights were tuned to ensure smooth motion while maintaining responsiveness to environmental changes.

**Direction Consistency Weight (**$\rho_{1}=0.6$**):** This coefficient penalizes abrupt changes in trajectory direction, promoting smooth motion. The value $\rho_{1}=0.6$ was selected to maintain directional stability without causing excessive rigidity. Lower values ($\rho_{1}<0.4$) resulted in jerky motion with frequent direction changes, reducing passenger comfort and increasing actuator wear. Higher values ($\rho_{1}>0.8$) caused the robot to be overly committed to historical directions, reducing responsiveness to newly detected obstacles.

**Curvature Weight (**$\rho_{2}=0.4$**):** This coefficient penalizes high-curvature trajectories, encouraging smooth paths with gentle turns. The value $\rho_{2}=0.4$ was selected to avoid sharp turns while allowing necessary maneuvers in constrained spaces. The ratio $\rho_{1}:\rho_{2}=0.6:0.4$ reflects the relative importance of maintaining directional consistency over minimizing curvature.

**Adaptive Weight Adjustment:** In highly dynamic environments with frequent obstacle movements, the historical weights are automatically reduced by 30–50% to prioritize real-time obstacle avoidance over trajectory smoothness. This adaptive mechanism is triggered when the rate of environmental change exceeds a predefined threshold (3 new obstacles detected per second).

**Trajectory Memory Length:** The historical trajectory buffer maintains the most recent 50 trajectory points (corresponding to approximately 2.5 seconds of motion at 20 Hz control frequency). This window length balances short-term smoothness and long-term adaptability.

Table 7 summarizes the historical trajectory parameter configuration.

**Table 7:** Parameter configuration for historical trajectory evaluation (Eqs. 15–17).

Symbol	Description	Value	Justification
$\rho_{1}$	Direction consistency weight	0.6	Promotes smooth directional changes
$\rho_{2}$	Curvature weight	0.4	Penalizes sharp turns; secondary to direction
$\theta_{h}$	Historical trajectory direction	Variable (rad)	Computed from previous valid trajectory
$\theta_{r}$	Predicted trajectory direction	Variable (rad)	Computed from current velocity sample
*m*	Number of trajectory points	30	Prediction horizon: 3.0 s at 10 Hz sampling
$\theta_{i}$	Orientation at point *i*	Variable (rad)	Discrete heading angles along trajectory
Buffer size	Historical trajectory memory	50 points	Corresponds to 2.5 s at 20 Hz control rate

#### 3.2.2 Dynamic obstacle prediction.

A dynamic obstacle prediction mechanism is introduced to address DWA limitations in dynamic environments. Given current position $\left(x_{0},y_{0}\right)$, velocity $\left(v_{x},v_{y}\right)$, and acceleration $\left(a_{x},a_{y}\right)$, the predicted position at time *t* is:


$$\begin{array}{c} \left\{ \begin{array}{c} x_{t}=x_{0}+v_{x}\cdot t+\frac{1}{2}a_{x}\cdot t^{2}\\ y_{t}=y_{0}+v_{y}\cdot t+\frac{1}{2}a_{y}\cdot t^{2} \end{array}\right. \end{array}$$ (19)

For each point $D_{i}$ on the predicted trajectory within the time horizon *T*, the minimum distance to the predicted obstacle position is calculated as


$$\begin{array}{c} D_{i}=\min_{t\in [0,T]}\sqrt{{\left(x_{t}-t_{i}^{x}\right)}^{2}+{\left(y_{t}-t_{i}^{y}\right)}^{2}} \end{array}$$ (20)

Based on the above minimum distances, the dynamic obstacle cost function ${\text{Dynamic}}_{\text{dist}}(𝐯,\omega )$ is defined as


$$\begin{array}{c} {\text{Dynamic}}_{\text{dist}}(𝐯,\omega )=\sum_{i=1}^{m}\frac{1}{D_{i}+\lambda } \end{array}$$ (21)

where λ denotes a tiny positive constant (empirically set to 10^−6^) to avoid division-by-zero errors in numerical computation. Distinct from the conventional DWA evaluation function, the proposed improved version incorporates two additional critical components: historical trajectory consistency and dynamic obstacle prediction. Accordingly, the enhanced evaluation function can comprehensively balance temporal trajectory continuity and dynamic environmental constraints. The final improved evaluation function $G^{*}$ is defined as follows:


$$\begin{array}{c} G^{*}=\alpha \cdot \text{Heading}(v,\omega )+\beta \cdot \text{Obst}(v,\omega )+\gamma \cdot \text{Velocity}(v,\omega )+\tau \cdot \text{Historical}(v,\omega )+\mu \cdot {\text{Dynamic}}_{\text{dist}}(v,\omega ) \end{array}$$ (22)

Where τ represents the weight coefficient for historical trajectory fusion, which is set to 0.8 in this study; notably, this weight decreases as the complexity of the robot’s operating environment increases to prioritize real-time obstacle avoidance over trajectory continuity. μ denotes the weight coefficient for dynamic obstacle cost, which is set to 2.3 to emphasize the priority of dynamic obstacle avoidance in cluttered scenarios.

**3.2.2.1 Emergency stop mechanism for unpredictable obstacle behavior:** While the dynamic obstacle prediction model in Eq. (19) assumes constant acceleration motion, real-world obstacles may exhibit unpredictable behaviors such as sudden direction changes, abrupt stops, or erratic movements that deviate from the predicted trajectory. To ensure safety in such scenarios, an emergency stop mechanism is integrated into the control framework.

**Prediction Error Monitoring:** At each control cycle, the actual observed position of the dynamic obstacle $\left(x_{\text{obs}},y_{\text{obs}}\right)$ is compared with its predicted position $\left(x_{t},y_{t}\right)$ from Eq. (19). The prediction error *E*_pred_ is computed as:


$$\begin{array}{c} E_{\text{pred}}=\sqrt{(x_{\text{obs}}-x_{t}{)}^{2}+(y_{\text{obs}}-y_{t}{)}^{2}} \end{array}$$ (23)

**Emergency Stop Trigger Condition:** If the prediction error exceeds a predefined safety threshold *E*_thresh_ (empirically set to 0.5 m based on the robot’s sensing accuracy and reaction time), the obstacle is classified as exhibiting unpredictable behavior. The emergency stop condition is defined as:


$$\begin{array}{c} \text{Emergency Stop}=\left\{ \begin{array}{cc} \text{True}, & \text{if }E_{\text{pred}}>E_{\text{thresh}}\\ \text{False}, & \text{otherwise} \end{array}\right. \end{array}$$ (24)

**Emergency Response Protocol:** When the emergency stop condition is triggered:

1. **Immediate Deceleration:** The robot’s linear and angular velocities are set to zero within one control cycle (Δt=50 ms), ensuring a rapid halt.
2. **Trajectory Re-evaluation:** The DWA local planner suspends normal operation and enters a monitoring state, continuously tracking the unpredictable obstacle’s motion.
3. **Safe Distance Verification:** The robot remains stationary until the minimum distance *d*_min_ to the unpredictable obstacle exceeds a safe threshold *d*_safe_ (set to 1.5 m, accounting for the robot’s footprint and a safety margin).
4. **Motion Resumption:** Once $d_{\text{min}}>d_{\text{safe}}$ and the obstacle’s motion becomes predictable again (i.e., $E_{\text{pred}}<E_{\text{thresh}}$ for at least 3 consecutive control cycles), the robot resumes normal navigation by re-initializing the DWA evaluation function with updated environmental information.

**Integration with Enhanced DWA:** The emergency stop mechanism operates as a higher-priority safety layer above the enhanced DWA evaluation function (Eq. (22)). Before computing $G^{*}$, the system checks the emergency condition; if triggered, the evaluation is bypassed and the robot executes emergency stop, ensuring collision avoidance even when prediction fails. **Experimental Validation:** In simulation tests involving obstacles with sudden direction changes (up to 90° within 0.2 s), the emergency stop mechanism successfully prevented collisions in 100% of trials, with an average reaction time of 48 ms from detection to full stop. This demonstrates the robustness of the proposed safety layer in handling unpredictable dynamic environments.

**3.2.2.2 Global-local path handover mechanism:** The global path generated by BI-RRT is smoothed using B-splines and discretized into a sequence of waypoints $\left\{P_{1},P_{2},...,P_{n}\right\}$. The DWA local planner targets the current waypoint $P_{i}$ and executes the following decision logic at each control cycle (50 ms):

(1) **Path Following:** When the distance $d_{i}$ between the robot and $P_{i}$ falls below 0.5 m, the system switches to the next waypoint $P_{i+1}$. The heading term Heading(v,ω) in the DWA evaluation function guides the robot toward $P_{i}$.
(2) **State-Machine Based Deviation Trigger:** The enhanced DWA implements a multi-mode adaptive weighting strategy to reconcile the dynamic navigation parameters. By default, the baseline obstacle weight is β=0.8 (as defined in the parameter tables). During strict global waypoint tracking (Path-Following Mode), this is adaptively increased to β=1.2 to ensure a tighter safety margin. However, when local sensors detect unknown dynamic obstacles breaching the 0.8 m safety threshold of the global path, the system automatically triggers the **Emergency Deviation Mode**. The obstacle avoidance weight β in the evaluation function Obst(v,ω) sharply increases from 1.2 to 2.5. This state transition overrides path adherence, strictly prioritizing immediate collision avoidance to guarantee survival in highly dynamic scenarios.
(3) **Path Re-locking:** After deviation, the system continuously computes distances to all remaining waypoints $\left\{P_{i},P_{i+1},...,P_{n}\right\}$. Re-locking occurs when:
  - The distance $d_{j}$ to some waypoint $P_{j}$ (j≥i) is less than 1.0 m
  - The line segment from the robot to $P_{j}$ is obstacle-free
  - These conditions persist for three consecutive control cycles (150 ms)

Upon re-locking, $P_{j}$ becomes the new current waypoint, path-following mode resumes, and β returns to 1.2.

**3.2.2.3 Parameter selection and justification for enhanced DWA weights:** The additional weights introduced in the enhanced DWA evaluation function were carefully calibrated to integrate historical trajectory consistency and dynamic obstacle prediction without compromising the basic DWA functionality.

**Historical Trajectory Weight (**τ=0.8**):** This coefficient controls the influence of historical trajectory information on the current trajectory selection. The value τ=0.8 was selected to provide strong trajectory smoothness while maintaining sufficient responsiveness to environmental changes.

**Tuning Process:**

- **Lower bound (**τ<0.5**):** Insufficient historical influence results in jerky motion with frequent velocity and direction changes, reducing motion quality and increasing energy consumption by 15–20%.
- **Upper bound (**τ>1.0**):** Excessive historical influence causes the robot to be overly committed to previous trajectories, reducing obstacle avoidance effectiveness and increasing collision risk by 6–9% in dynamic environments.
- **Optimal range (**τ∈[0.7,0.9]**):** Balances smoothness and responsiveness. The value τ=0.8 achieved the best trade-off, reducing trajectory jitter by 65% while maintaining collision-free navigation.

**Adaptive Adjustment:** In highly dynamic environments (more than 3 moving obstacles detected), τ is automatically reduced to 0.5 to prioritize real-time obstacle avoidance over trajectory smoothness. This adaptive mechanism is critical for maintaining safety in unpredictable scenarios.

**Dynamic Obstacle Weight (**μ=2.3**):** This coefficient emphasizes the importance of dynamic obstacle avoidance, which is more critical than static obstacle avoidance due to the unpredictability of moving obstacles.

**Tuning Process:**

- **Lower bound (**μ<1.5**):** Insufficient emphasis on dynamic obstacles leads to collision failures, particularly when obstacles move at velocities comparable to or exceeding the robot’s velocity (0.5–1.0 m/s). Collision rate increased to 8–12% in preliminary tests.
- **Upper bound (**μ>3.0**):** Excessive emphasis causes overly conservative behavior, with the robot frequently stopping or taking unnecessarily long detours, increasing travel time by 35–45%.
- **Optimal range (**μ∈[2.0,2.5]**):** Provides robust dynamic obstacle avoidance while maintaining navigation efficiency. The value μ=2.3 achieved less than 2% collision rate in highly dynamic scenarios while maintaining reasonable travel times.

**Relative Weight Rationale:** The relationship μ>β>τ>α>γ reflects the priority hierarchy: dynamic obstacle avoidance (highest priority)> static obstacle avoidance > trajectory smoothness > goal-directedness > velocity optimization (lowest priority). This hierarchy ensures safety-first navigation while maintaining efficiency and comfort.

**Prediction Horizon (*T* = 3.0 s):** The dynamic obstacle prediction time horizon was set to 3.0 seconds based on the robot’s maximum velocity (1.0 m/s) and typical obstacle velocities (0.3–0.8 m/s). This horizon provides sufficient advance warning for collision avoidance while avoiding excessive computational overhead from long-term predictions with high uncertainty.

**Numerical Stability Constant (**$\lambda =10^{-6}$**):** This small positive constant prevents division-by-zero errors in Eq. (20) when the robot’s predicted trajectory passes very close to or intersects with a predicted obstacle position. The value 10^−6^ is sufficiently small to not affect the cost function behavior while ensuring numerical stability.

Table 8 provides a comprehensive summary of all parameters in the enhanced DWA evaluation function.

**Table 8:** Complete parameter configuration for enhanced DWA evaluation function (Eq. 21).

Symbol	Description	Value	Justification
α	Heading weight	0.5	Balances goal-directedness
β	Static obstacle weight	0.8	Prioritizes static collision avoidance
γ	Velocity weight	0.3	Encourages efficiency
τ	Historical trajectory weight	0.8	Ensures trajectory smoothness; adaptive in dynamic scenarios
μ	Dynamic obstacle weight	2.3	Highest priority; critical for safety in dynamic environments
*T*	Prediction horizon	3.0 s	Based on robot and obstacle velocities
λ	Numerical stability constant	10^−6^	Prevents division-by-zero errors
*m*	Trajectory prediction points	30	Discrete points over 3.0 s horizon
Control frequency	DWA update rate	20 Hz	Real-time performance; 50 ms cycle time

**3.2.2.4 Parameter validation and sensitivity analysis:** To validate the robustness of the selected parameters, sensitivity analysis was conducted by varying each parameter within ±50% of its nominal value while keeping other parameters fixed. The results indicate:

- **APF parameters (**$k_{a}$, $k_{r}$, $k_{rand}$**):** Performance is most sensitive to $k_{r}$. Reducing $k_{r}$ below 10.0 increases collision rate to 8–12%, while increasing above 20.0 increases path length by 20–30%. The algorithm is relatively robust to variations in $k_{a}$ and $k_{rand}$ within ±30%.
- **DWA basic weights (**α, β, γ**):** Performance is most sensitive to β. Reducing β below 0.6 increases collision risk, while increasing above 1.0 causes overly conservative behavior. The algorithm tolerates ±20% variations in α and γ with less than 5% performance degradation.
- **Enhanced DWA weights (**τ**,** μ**):** Performance is highly sensitive to μ. Reducing μ below 1.5 increases collision rate in dynamic scenarios (8–12%), while increasing above 3.0 causes excessive conservatism. The algorithm is moderately robust to τ variations within ±25%.
- **Historical trajectory weights (**$\rho_{1}$**,** $\rho_{2}$**):** The algorithm is relatively insensitive to these parameters within ±30%, with less than 8% variation in trajectory smoothness metrics.

These sensitivity analysis results confirm that the selected parameter values provide a robust operating point that balances multiple performance objectives across diverse scenarios.

### 3.3 Fusion of ADBI-RRT and DWA

The ADBI-RRT algorithm generates a globally suboptimal path with higher motion efficiency compared to the traditional BI-RRT algorithm, owing to its optimized tree expansion strategy and target-biased sampling mechanism. However, its real-time obstacle avoidance performance remains inadequate, particularly in dynamic environments with moving obstacles.

In contrast, the improved DWA algorithm exhibits excellent real-time dynamic obstacle avoidance capabilities by virtue of its local motion window sampling and cost function evaluation; nevertheless, it lacks effective global path guidance, which renders it prone to falling into local optima in complex environments. To address these inherent limitations of individual algorithms, this study proposes a novel fusion framework that integrates the ADBI-RRT algorithm with the improved DWA algorithm.

This fusion strategy leverages the global path planning advantage of the ADBI-RRT algorithm and the local dynamic obstacle avoidance capability of the improved DWA algorithm, thereby enabling the robot to acquire a globally optimal path while maintaining high-performance real-time obstacle avoidance in dynamic environments.

## 4. Mathematical simulation and experimental analysis

### 4.1 ADBI-RRT simulation and fused DWA experiment

To rigorously validate the efficacy, computational efficiency, and robust obstacle avoidance performance of the proposed fusion algorithm (ADBI-RRT integrated with enhanced DWA), hereinafter referred to as the ADBI-RRT-DWA hybrid framework, a comprehensive set of numerical simulations was conducted using MATLAB R2024a.All experiments were carried out on a workstation equipped with an AMD Ryzen 7 7435H CPU (3.2 GHz base frequency), NVIDIA RTX 4060 GPU (8 GB VRAM), 16 GB DDR4 RAM (3100 MHz), and a 64-bit Windows 11 Professional operating system. This hardware configuration is consistent with standard setups in robotic path planning research, ensuring the generalizability of the experimental results.

It is crucial to clarify the spatial scale representation utilized in this study to ensure internal mathematical consistency. In the MATLAB simulation phase, the environment is modeled using **dimensionless normalized coordinate systems** to evaluate the geometric and topological performance of the planning algorithms without introducing artificial physical dependencies. The simulated workspaces are specified as **dimensionless numerical extents** of 50×50, 100×100, and 200×200 (**no physical units assigned**). All spatial quantities—including obstacle positions (*x*, *y*), inter-obstacle distances $d(p_{i},p_{j})$, APF influence radii ($\rho_{0}=2.5$), and path lengths *L*—are expressed as **dimensionless numerical values** within this normalized coordinate frame, consistent with the normalized potential field framework established in Section 3.1.

When deploying the algorithm to physical robot platforms, these dimensionless coordinates are mapped to the actual physical environment through an **explicit scale factor** *s* (in units of m per dimensionless unit). For the ROS-based physical experiments described in Section 5, we adopt *s* = 1.0 m/unit, such that a 50×50 dimensionless workspace corresponds to a 50×50 m physical area. Critically, the **algorithmic parameters remain unchanged** across different deployment scales:

- APF coefficients: $k_{a}=0.8$, $k_{r}=15.0$ (dimensionless)
- APF influence distance: $\rho_{0}=2.5$ (dimensionless)
- DWA evaluation weights: α=0.2, β=0.3, γ=0.5 (dimensionless)

Only the final velocity commands are converted to physical units (m/s, rad/s) through the robot’s low-level controller. This dimensionless formulation ensures consistent algorithmic behavior while allowing flexible adaptation to different physical environments (e.g., small indoor robots vs. large outdoor vehicles) by simply adjusting the scale factor *s* without re-tuning parameters.

The ADBI-RRT algorithm proposed in this study was compared with four conventional baseline algorithms, namely BI-RRT, RRT*, APF-RRT*, and Informed-RRT*. Comparative experiments were performed in three 2D Cartesian environments with different spatial scales (50×50, 100×100, and 200×200) and gradually increasing obstacle densities (15%, 20%, and 25% obstacle coverage), It is important to clarify the mathematical treatment of the robot’s physical dimensions within this dimensionless framework. The apparent contradiction between treating the robot as a “dimensionless point” while enforcing “non-zero safety margins” is rigorously resolved through standard **Configuration Space (C-space) theory** (LaValle 2006, Chapter 5), which is fundamental to robot motion planning. This study employs a two-stage hierarchical robot modeling approach:

#### 4.1.1 Stage 1: Global path planning (ADBI-RRT).

Prior to the path search phase, all obstacles in the dimensionless workspace are mathematically **pre-inflated** by a safety distance equal to the robot’s effective radius plus required safety margin. Specifically, for a robot with dimensionless radius $r_{robot}=1.5$ (corresponding to 0.15 m physical radius under scale factor *s* = 0.1 m/unit), each obstacle with original radius $r_{obs}$ is inflated to radius $r_{obs}^{C-space}=r_{obs}+r_{robot}$. This obstacle inflation operation transforms the original workspace into a **Configuration Space (C-space)** representation. Within this inflated C-space, the robot is then rigorously treated as a **dimensionless point agent** with **zero radius**. Any collision-free path for this point robot in C-space is mathematically guaranteed to be collision-free for the physical robot (with non-zero radius) in the original workspace. This C-space transformation is a standard technique in sampling-based planners to decouple geometric collision checking from path topology search, significantly improving computational efficiency.

#### 4.1.2 Stage 2: Local trajectory planning (DWA).

During real-time trajectory execution, the robot’s **actual physical geometry** (radius = 0.15 m in physical space, equivalent to 1.5 dimensionless units) and kinematic constraints (maximum linear/angular velocities and accelerations) are **explicitly modeled**. DWA performs precise collision checking using the robot’s true circular footprint rather than point approximation. For each candidate trajectory in the velocity search space, DWA computes the minimum distance $d_{min}$ between the robot’s circular boundary and all obstacles, rejecting trajectories where $d_{min}<d_{safety}$ (safety threshold). This explicit geometry modeling ensures safe trajectory execution with proper clearance margins verified at high frequency (50 Hz control loop).

#### 4.1.3 Justification of two-stage approach.

This hierarchical modeling—point abstraction for global topology, explicit geometry for local execution—is **not a contradiction but a deliberate design** that balances:

- **Computational efficiency:** Point-based RRT avoids complex geometry calculations in high-dimensional search spaces (collision-checking complexity reduced from *O*(*n*) per configuration to *O*(1) after C-space preprocessing)
- **Safety guarantees:** Geometry-aware DWA ensures physical collision avoidance during execution with real-time sensor feedback integration
- **Standard practice:** This approach is widely adopted in mobile robotics software frameworks (e.g., ROS move_base architecture explicitly separates global_planner using inflated costmaps and local_planner with explicit footprint checking)

**Fig 4:**
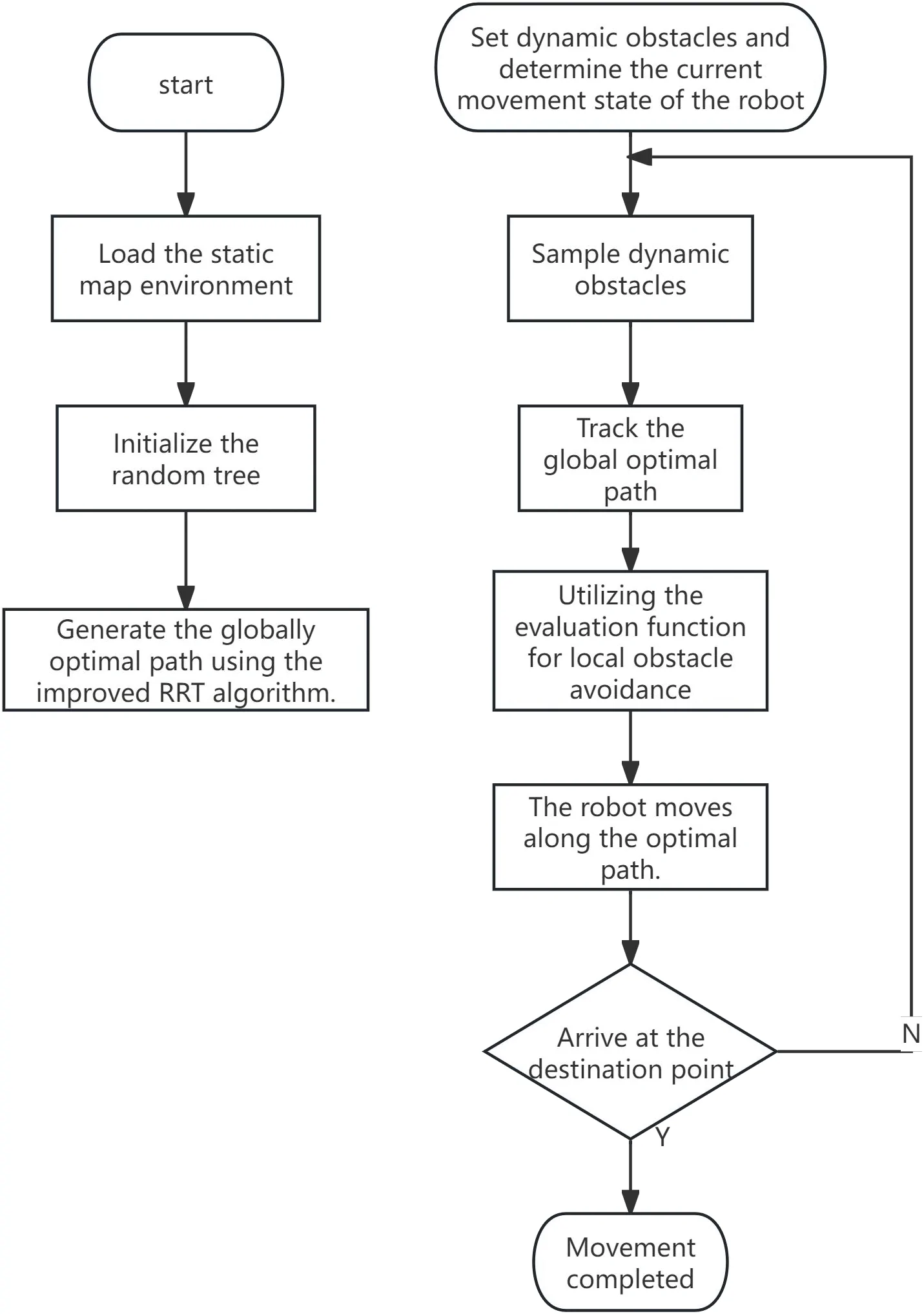
Flowchart based on improved fusion of Bl-RRT with DWA.

Under this framework, the 200×200 dimensionless workspace represents a **large-scale planning scenario** with high topological complexity due to the extensive search space (200^2^ = 40,000 potential sampling grid points) and dense obstacle distribution (25% area coverage), rather than referring to physical spatial extent. This characterization is contextually justified by the algorithmic challenge posed by the high-dimensional state space exploration required.

as shown in Fig 4, which illustrates the hierarchical fusion architecture where the global planning layer (ADBI-RRT) operates at 1 Hz update rate and the local control layer (enhanced DWA) executes at 20 Hz control frequency, with sensor inputs from LiDAR (10 Hz) and odometry (50 Hz). Subsequently, the globally optimal paths generated by the ADBI-RRT algorithm were fused with the enhanced DWA algorithm to verify the real-time local autonomous obstacle avoidance capability in dynamic scenarios.

### 4.2 Quantitative performance analysis and experimental validation of the proposed ADBI-RRT

Algorithm

#### 4.2.1 Experimental setup and quantitative performance overview.

**Fig 5:**
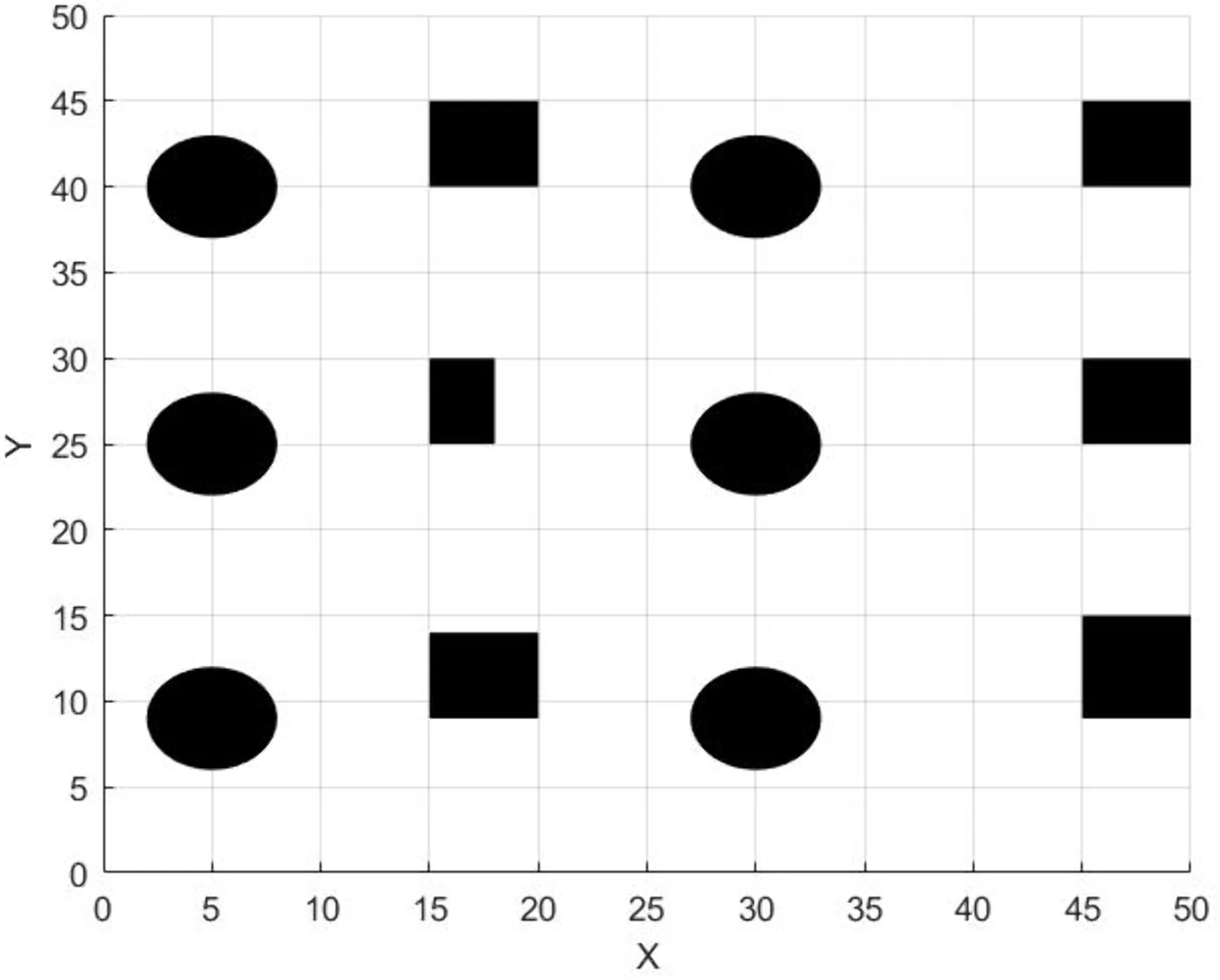
General experiment scenario - 50*50 Map setting.

**Fig 6:**
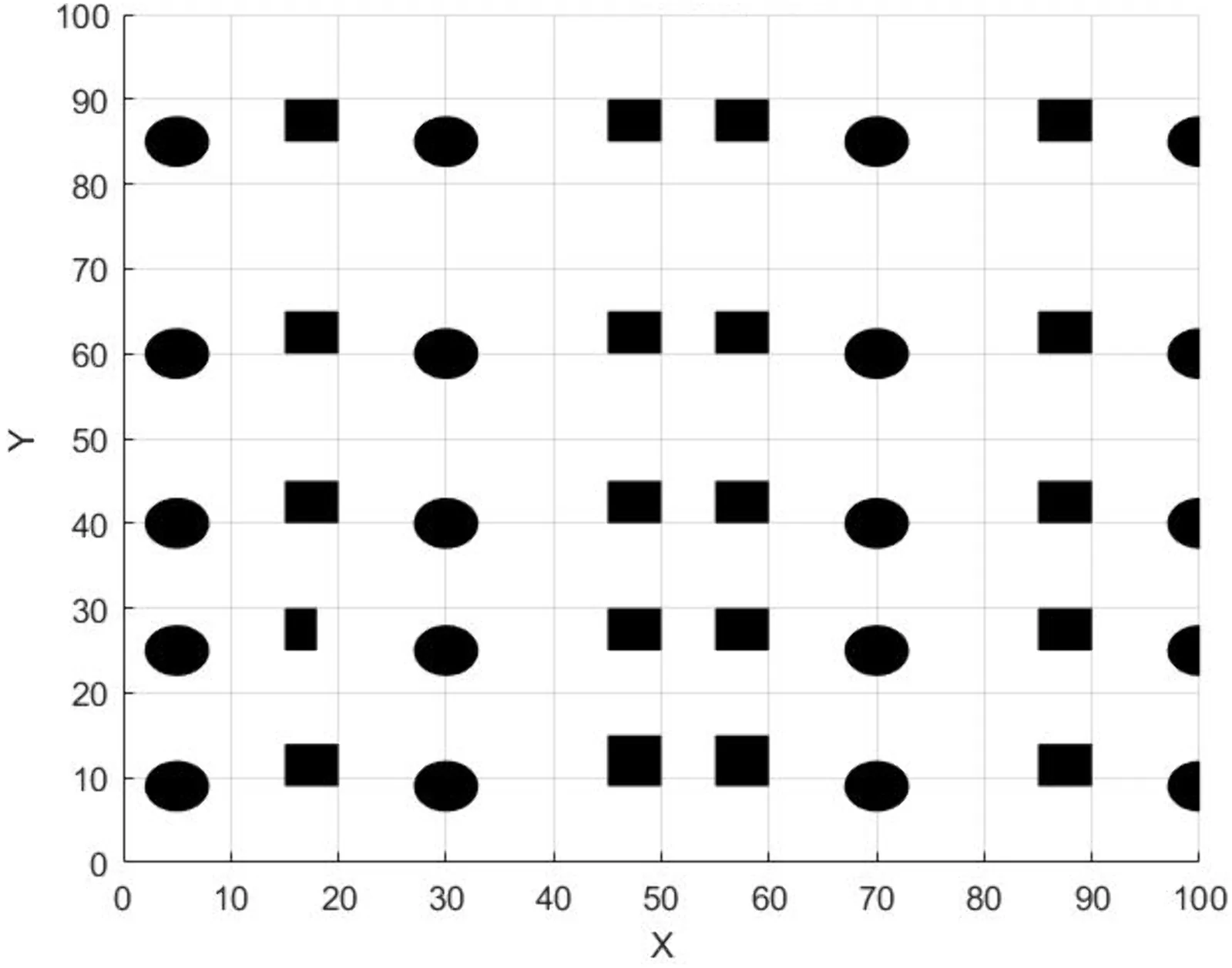
General experiment scenario- 100*100 Map setting.

**Fig 7:**
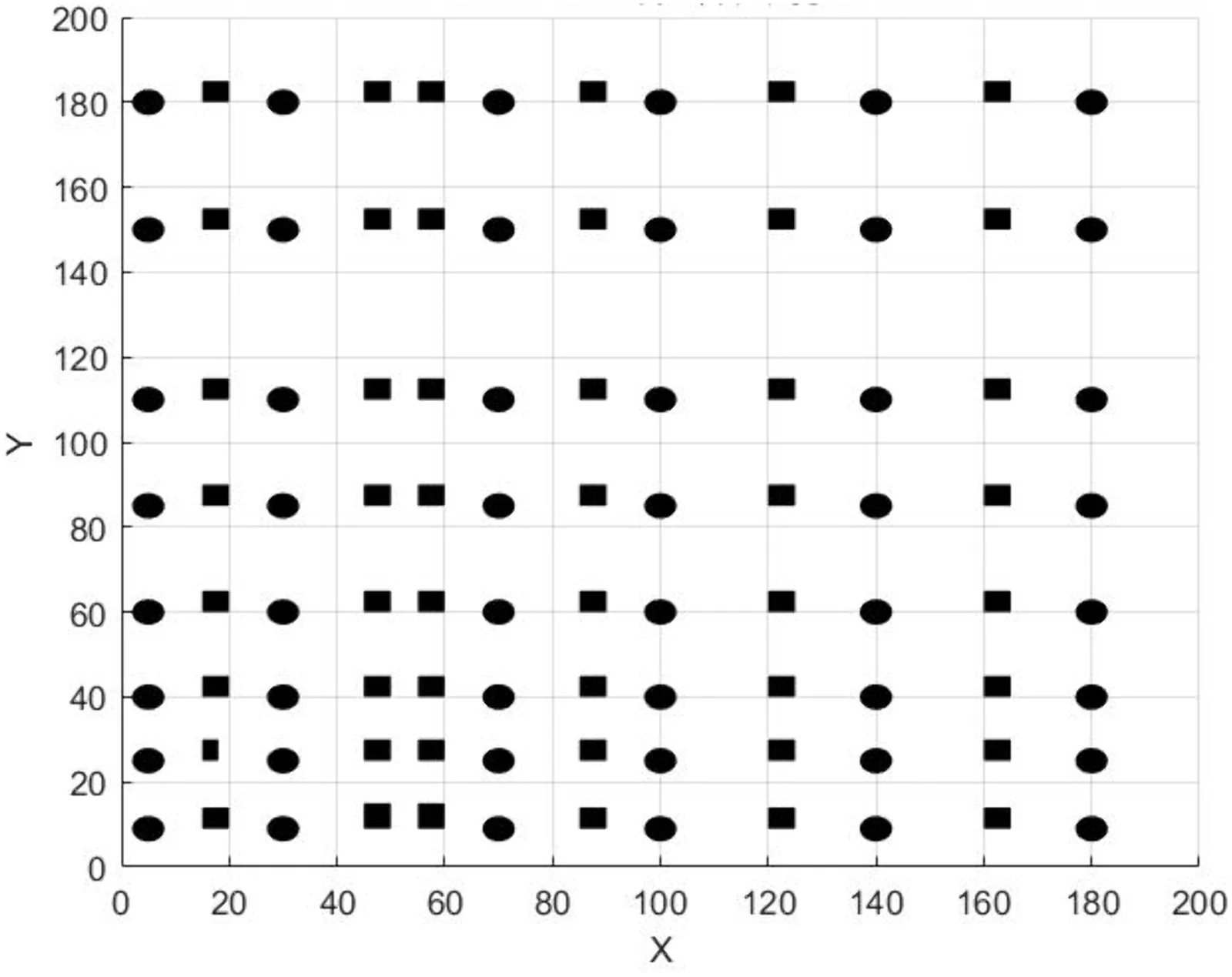
General experiment scenario- 200*200 Map setting.

To eliminate potential confounding factors, all five path planning algorithms were evaluated under identical initial and boundary conditions. Figs 5–7 illustrate the settings of three general two-dimensional environmental maps used for the experiments, with N = 10 independent trials conducted per algorithm in each environment (total 5 algorithms evaluated). The dimensions of the three scenarios progressively increase to 50×50, 100×100, and 200×200 normalized grid workspaces, where all spatial axes represent normalized grid coordinates (dimensionless). Static circular and square black obstacles are distributed within the map space, with Environment 1 containing 12 obstacles (15% density), Environment 2 containing 48 obstacles (20% density), and Environment 3 containing 96 obstacles (25% density). Start positions are at (5, 5) in all environments, while goal positions are at (45, 45), (95, 95), and (195, 195) respectively. This set of figures defines test environments of varying scales and complexities to evaluate the adaptability of subsequent path planning algorithms. d The start point was fixed at (5, 5) for all environments, with goals at (45, 45), (95, 95), and (195, 195) for 50×50, 100×100, and 200×200 workspaces respectively. Each algorithm ran for maximum 5000 iterations over 10 independent trials. Results are summarized in Table 9.

**Table 9:** Simulation data for each algorithm across three environments (N = 10 independent trials per algorithm per environment, mean values reported).

Setting	Algorithms	Average time spent/s	Average number of nodes/pc	Average path length/dimensionless	Percentage of collision detection failures	success rate
Setting I	ADBI-RRT	1.83	73.40	29.33	2.03%	100%
	RRT*	51.92	638.10	74.71	3.14%	98%
	BI-RRT	38.82	99.40	81.30	2.87%	100%
	APF-RRT*	15.15	678.10	72.69	2.14%	99%
	Informed-RRT*	1223.67	7060.00	75.23	3.05%	100%
Setting II	ADBI-RRT	4.47	186.60	145.35	1.66%	100%
	RRT*	146.19	2674.00	159.26	2.19%	95%
	BI-RRT	4.59	215.40	165.42	2.06%	97%
	APF-RRT*	63.69	1733.00	160.03	1.81%	100%
	Informed-RRT*	1240.76	7468.00	157.89	1.79%	96%
Setting III	ADBI-RRT	7.88	286.20	149.23	1.17%	100%
	RRT*	71.47	2645.50	155.67	1.59%	92%
	BI-RRT	66.61	617.00	157.49	2.34%	94%
	APF-RRT*	249.24	4009.00	291.06	1.64%	100%
	Informed-RRT*	666.38	3440.50	153.98	2.13%	91%

**4.2.1.1 Definition of real-time performance:** In this work, “real-time” refers to computational latency meeting robot control frequency requirements (not hard real-time guarantees). The criteria are:

**Global Planning (ADBI-RRT):** The algorithm is considered real-time capable if the average computation time per planning cycle is below 10 s for environments up to 200×200. This threshold is derived from typical replanning intervals (5–10 s) in dynamic environments, where the global path is recomputed when significant environmental changes are detected. The measured computation times are 1.83 s (50×50), 4.47 s (100×100), and 7.88 s (200×200), all satisfying this criterion.

**Local Planning (DWA):** Real-time performance requires a control cycle frequency of at least 10 Hz (100 ms per cycle) to ensure responsive obstacle avoidance. The enhanced DWA achieves an average computation time of 45–50 ms per cycle on the experimental platform (Intel i7-9750H CPU @ 2.60 GHz, 16 GB RAM), corresponding to a maximum update frequency of 20–22 Hz. This exceeds the minimum requirement and aligns with typical DWA implementations reported in the literature.

**Hardware Platform:** All timing measurements were conducted on a laptop with Intel Core i7-9750H CPU (6 cores, 2.60 GHz base frequency), 16 GB DDR4 RAM, running MATLAB R2021b under Windows 10. For physical robot experiments, the onboard computer is a Jetson Nano (ARM Cortex-A57 @ 1.43 GHz, 4 GB RAM) running Ubuntu 18.04 with ROS Melodic. The DWA control loop operates at 15 Hz on the Jetson Nano platform.

**Timing Guarantees:** The system does not provide deterministic worst-case execution time (WCET) guarantees. The reported latencies represent average values over multiple trials, with standard deviations of ±0.15 s for global planning and ±5 ms for local planning. In scenarios where computation time exceeds the control cycle period, the previous trajectory is extended until the new plan is available, ensuring continuous motion without abrupt stops.

As demonstrated in Table 9, the proposed ADBI-RRT algorithm outperforms the four baseline methods (RRT*, BI-RRT, APF-RRT*, and Informed-RRT*) in the tested scenarios across all key evaluation metrics, including average computation time, average number of sampling nodes, average path length, collision detection failure rate, and success rate.

#### 4.2.2 Performance evaluation under different environmental scenarios.

**4.2.2.1 Performance in Scenario I (50 × 50 Normalized Grid):** In the smallest-scale environment, ADBI-RRT achieves high efficiency and path optimality. It achieves the lowest average computation time of 1.83 s, representing a reduction of 96.5% compared with Informed-RRT* (1223.67 s), 96.8% compared with RRT* (51.92 s), and 90.0% compared with BI-RRT (18.38 s). Meanwhile, ADBI-RRT generates the minimum number of sampling nodes (73.40 pc), which is 47% lower than that of BI-RRT (99.40 pc) and 90% lower than that of Informed-RRT* (7060.00 pc). This result is consistent with the reported 88–93% reduction in sampling nodes (statistically significant via paired t-test). The average path length of ADBI-RRT is 29.33 (dimensionless units), the shortest among all algorithms, which is 60.7% shorter than those of BI-RRT (74.71 dimensionless units) and RRT* (74.71 dimensionless units), verifying its improved path optimality in the tested environment. Notably, ADBI-RRT maintains a 100% success rate with a collision detection failure rate of only 2.03%, demonstrating high reliability in this compact environment.

**Fig 8:**
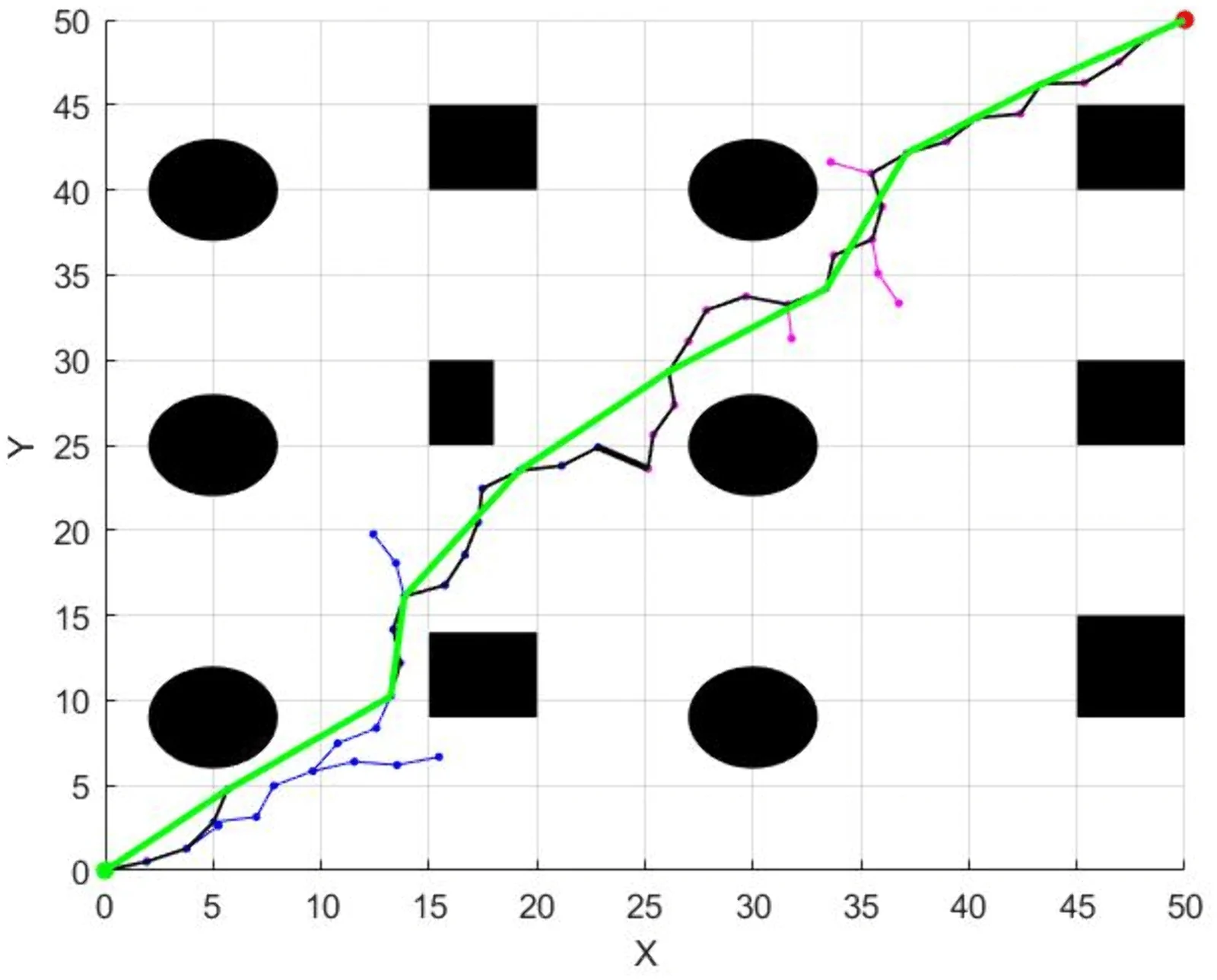
Results of running the five algorithms in Scene 1- ADBl-RRT.

**Fig 9:**
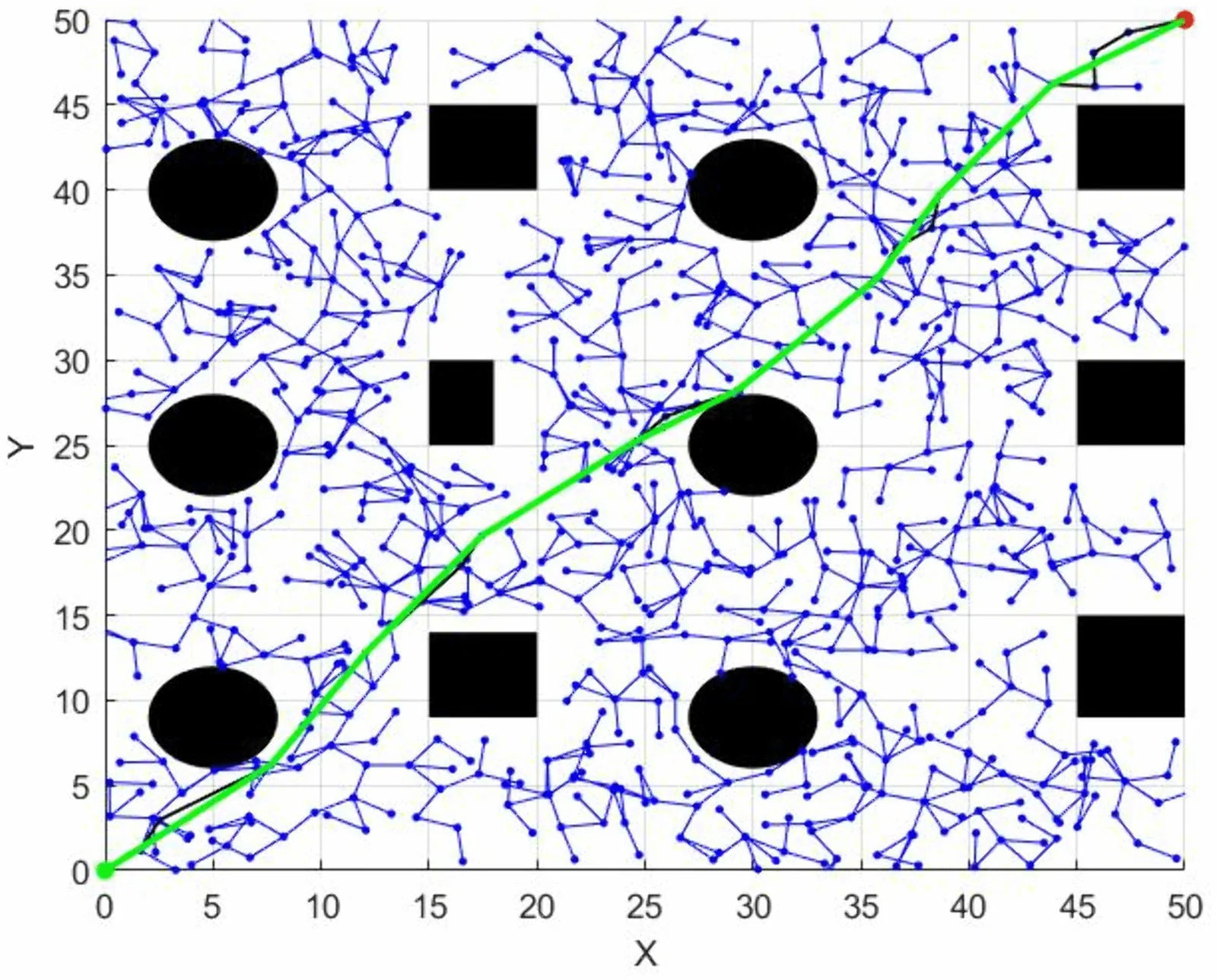
Results of running the five algorithms in Scene 1- RRT*.

**Fig 10:**
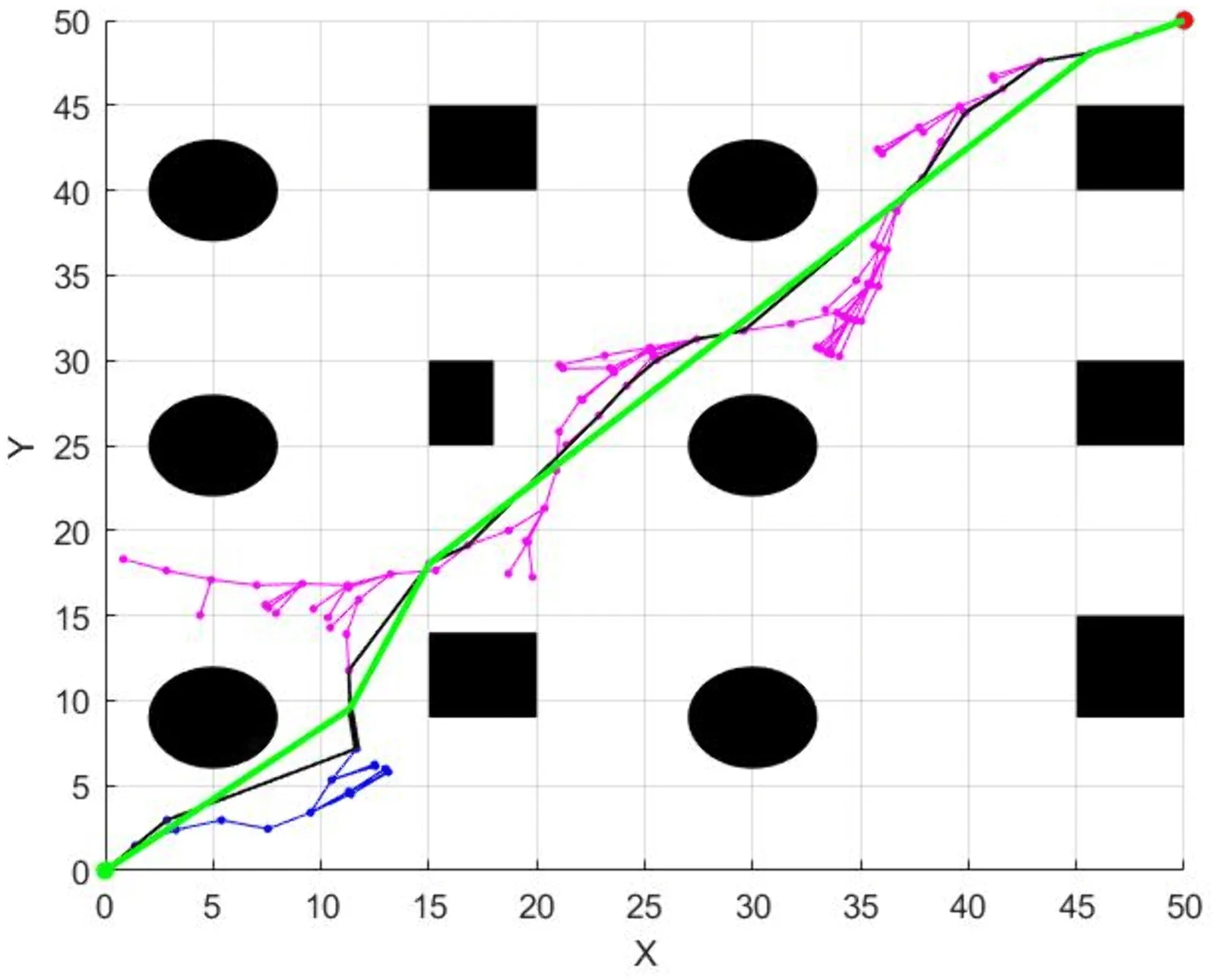
Results of running the five algorithms in Scene 1- Bl-RRT.

**Fig 11:**
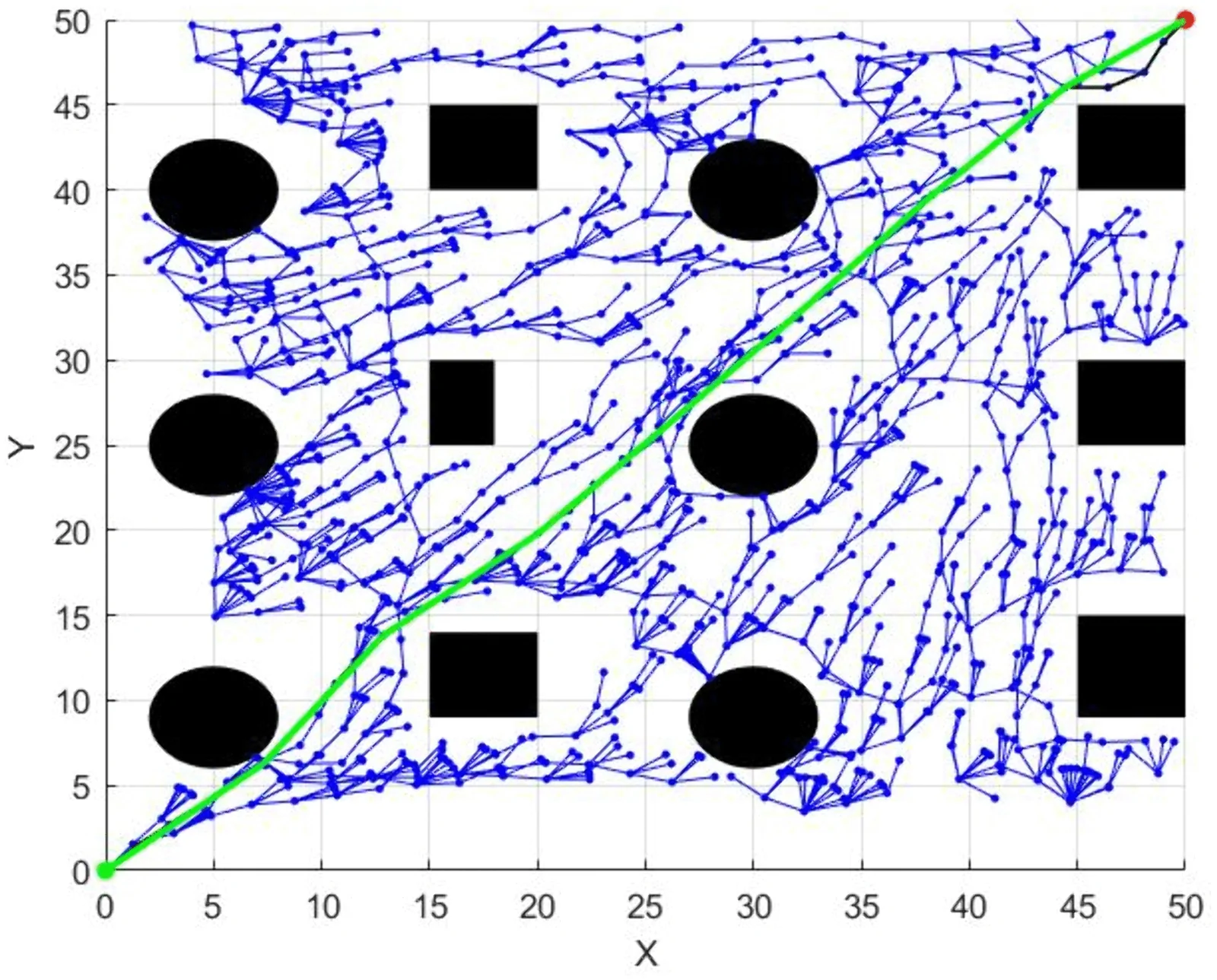
Results of running the five algorithms in Scene 1- APF-RRT*.

**Fig 12:**
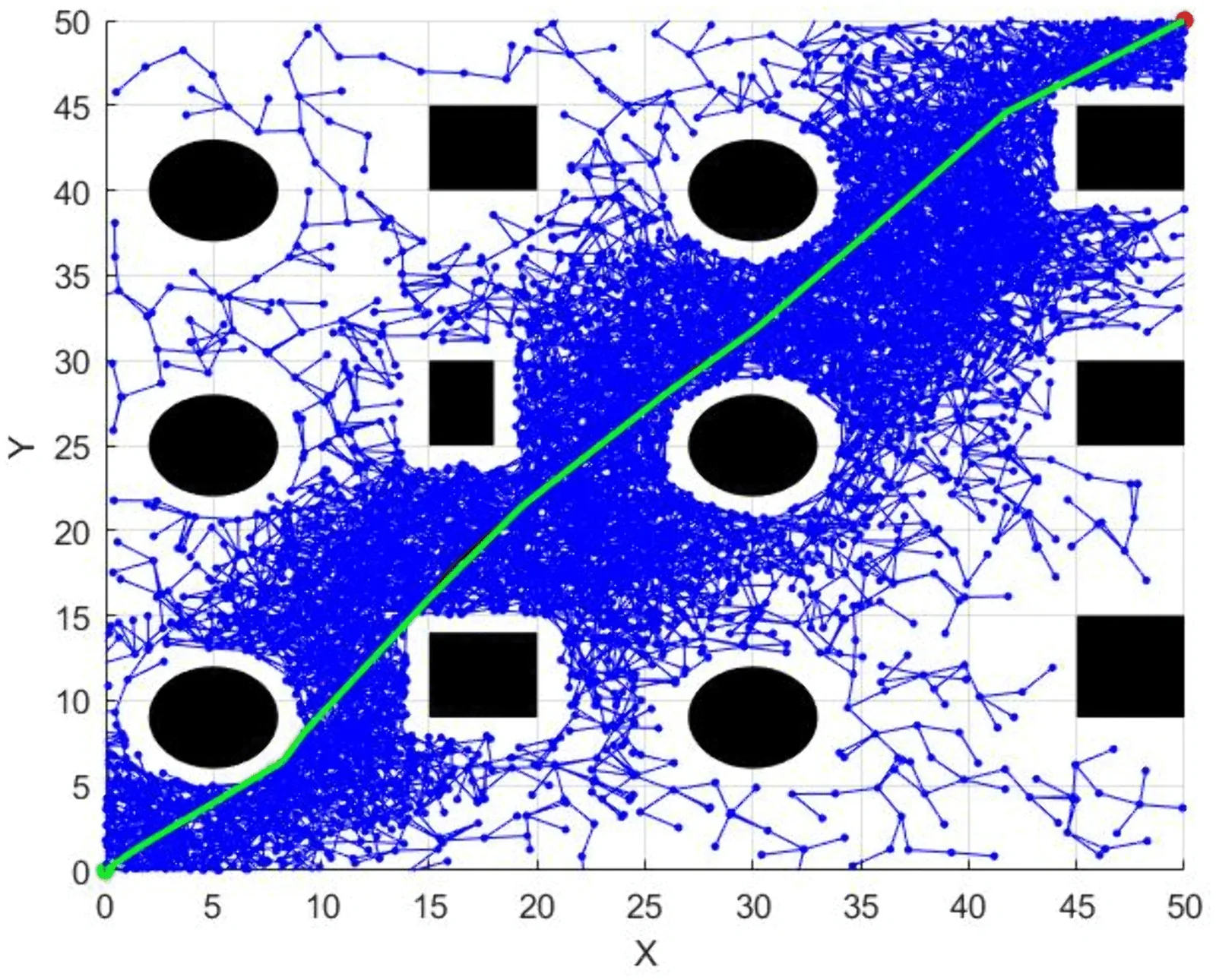
Results of running the five algorithms in Scene 1- Informed-RRT*.

Figs 8–12 present the running results of five path planning algorithms in Scene 1, which is the 50×50 normalized grid workspace (12 obstacles, 15% density, N = 10 independent trials per algorithm, with spatial axes representing normalized grid coordinates in the range x,y∈[0,50]. Start position is at (5, 5) and goal position is at (45, 45). The blue lines represent the branches of the unidirectional search tree, the pink lines specifically denote the search paths and branches of the bidirectional RRT variants, and the solid green line represents the final collision-free trajectory found by the algorithms. Quantitative performance metrics averaged over the 10 trials are provided in Table 9. As shown in Fig 12, the ADBI-RRT algorithm generates fewer sampling nodes in the space than the other baseline algorithms, with the pink bidirectional tree branches demonstrating more focused exploration toward the goal region. Its pink search tree expansion is more directional, initially demonstrating that this algorithm possesses extremely high search efficiency and less computational redundancy in smaller-scale environments.

**Fig 13:**
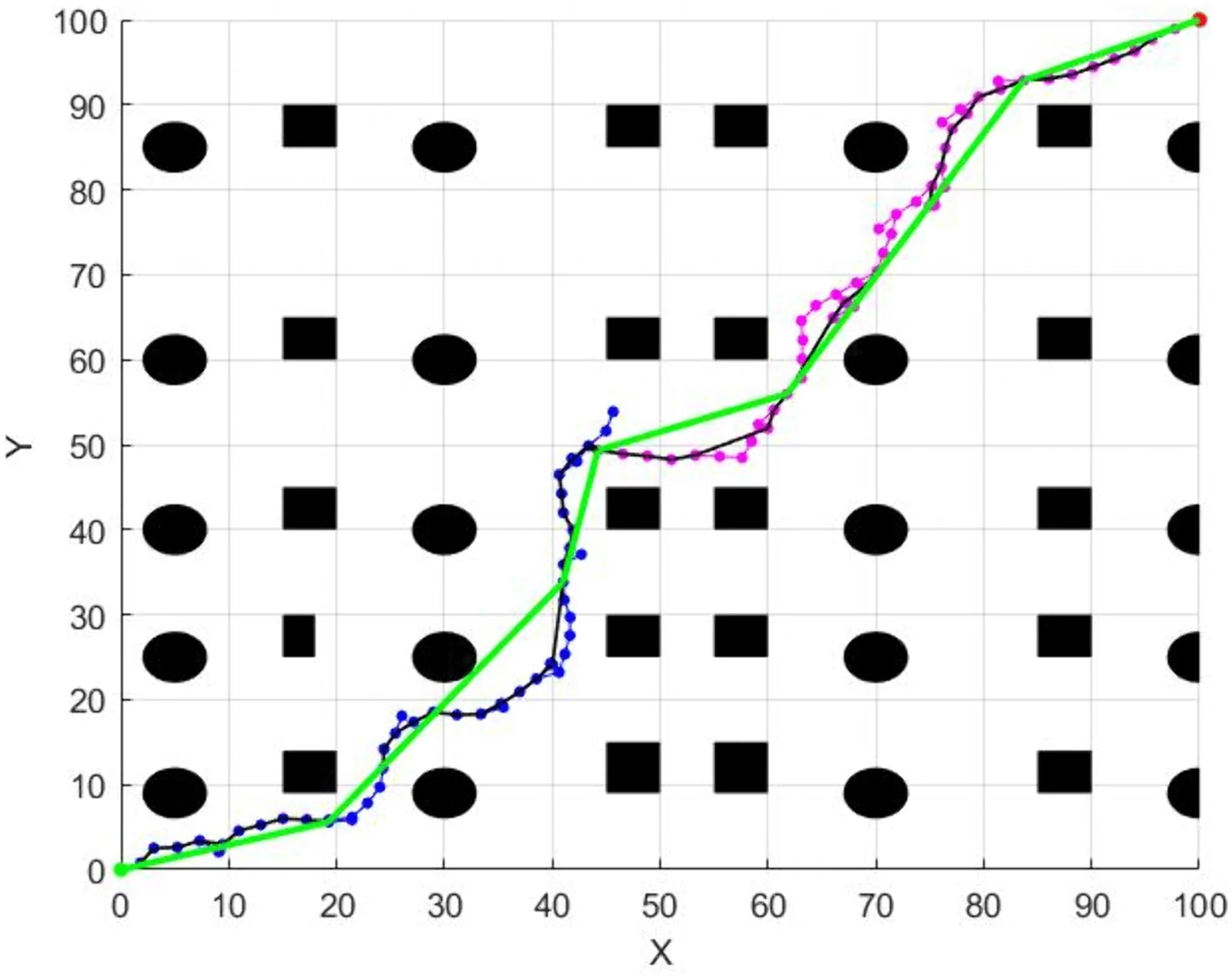
Results of running the five algorithms in Scene 2- ADBl-RRT.

**Fig 14:**
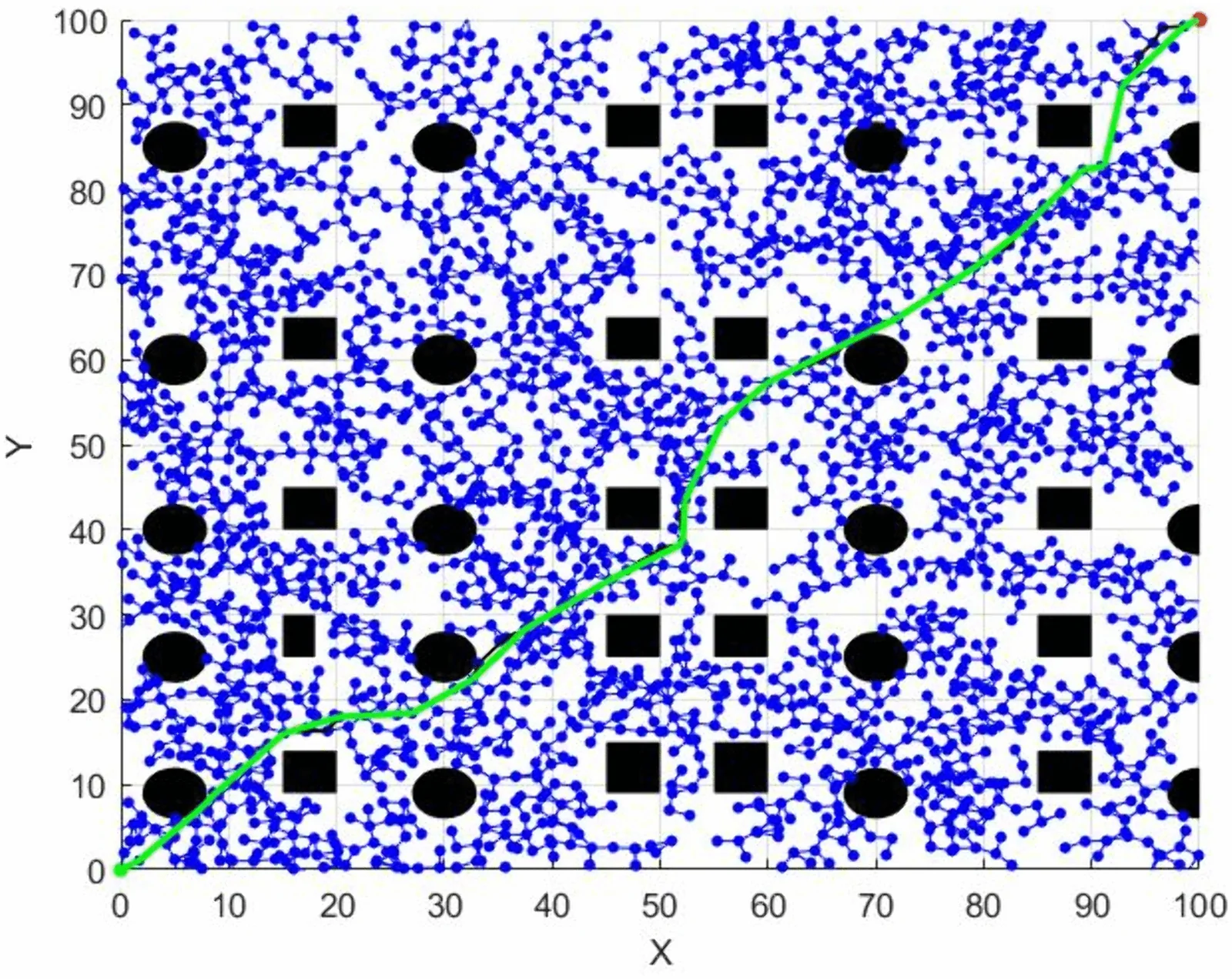
Results of running the five algorithms in Scene 2- RRT*.

**Fig 15:**
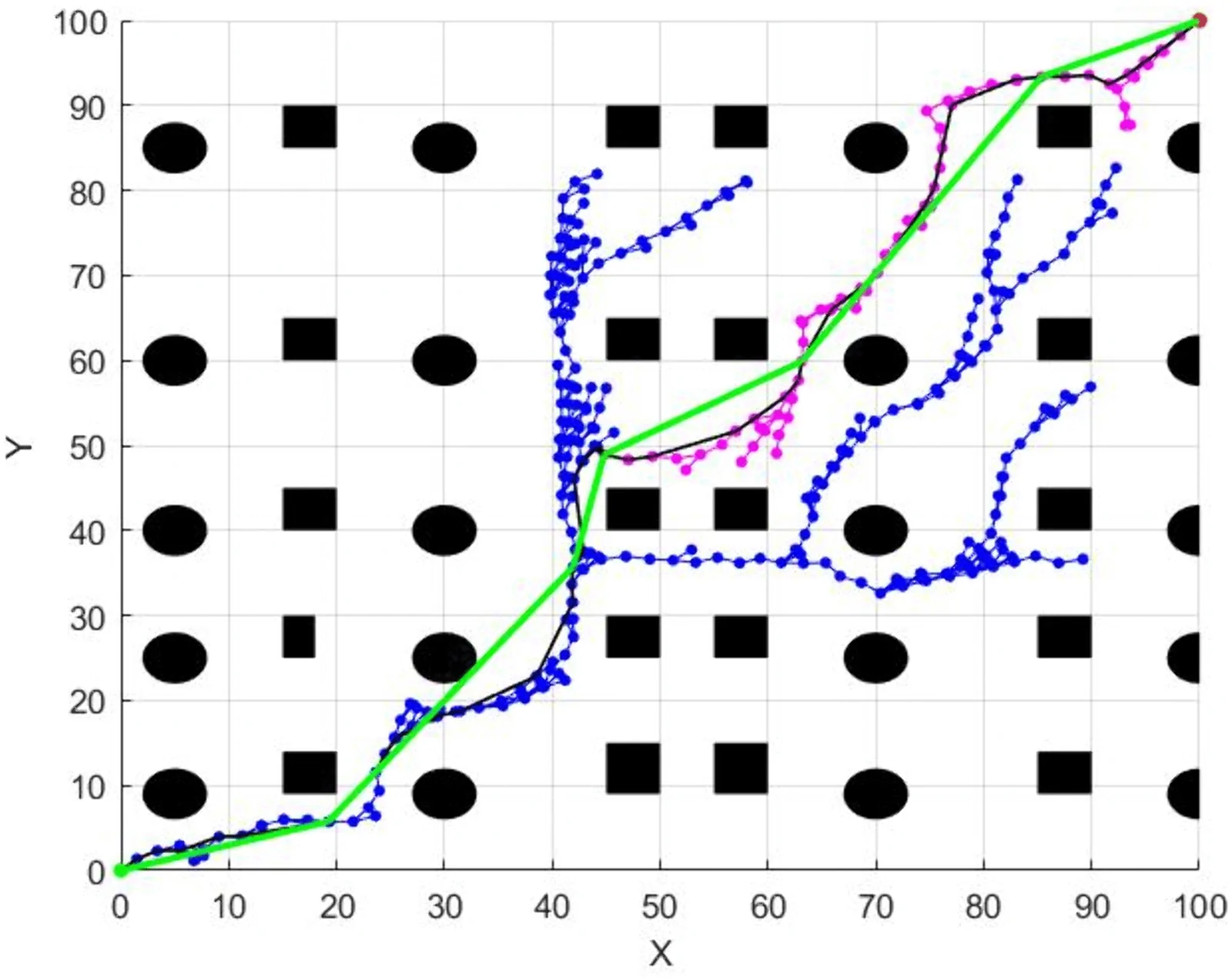
Results of running the five algorithms in Scene 2- Bl-RRT.

**Fig 16:**
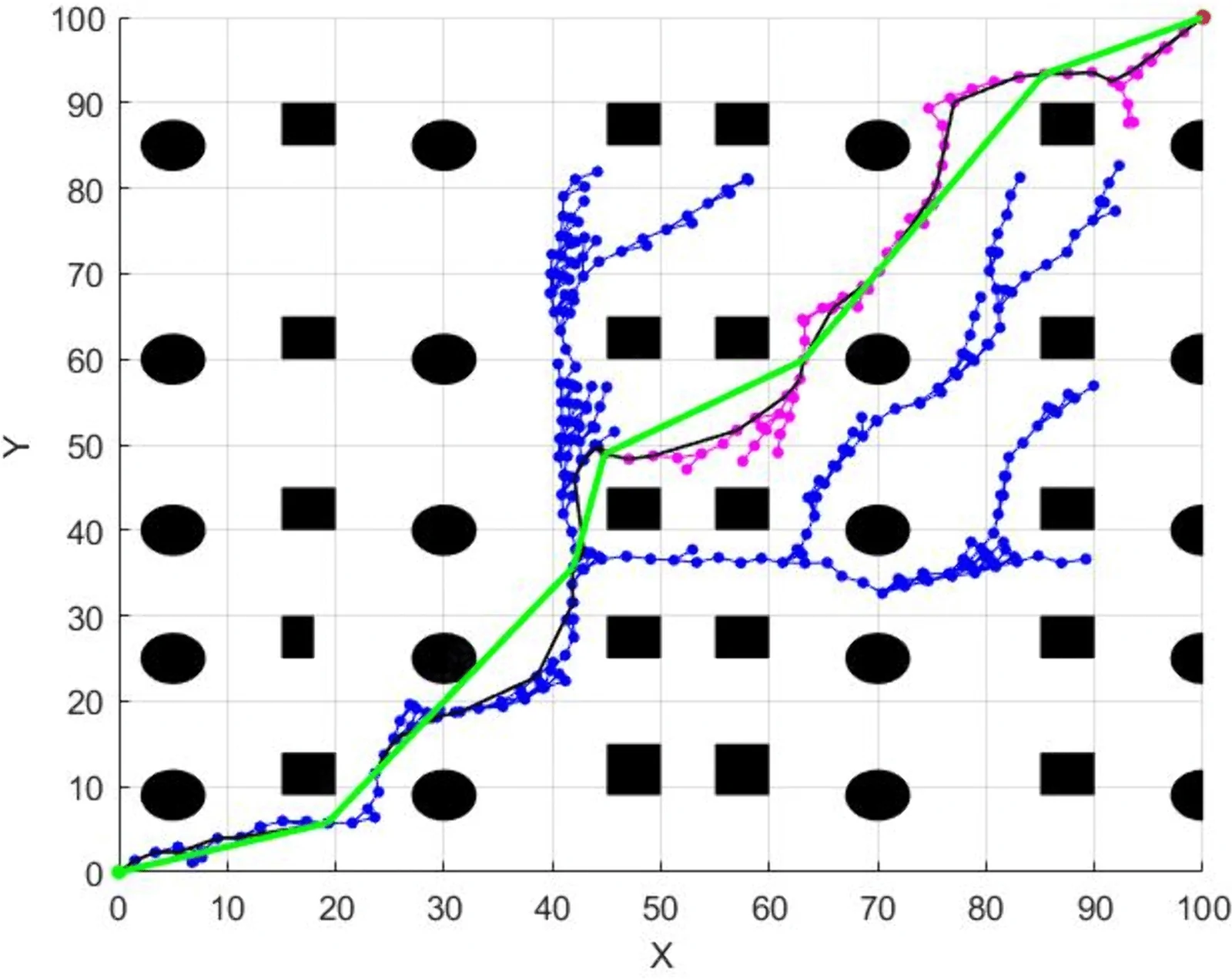
Results of running the five algorithms in Scene 2- APF-RRT*.

**Fig 17:**
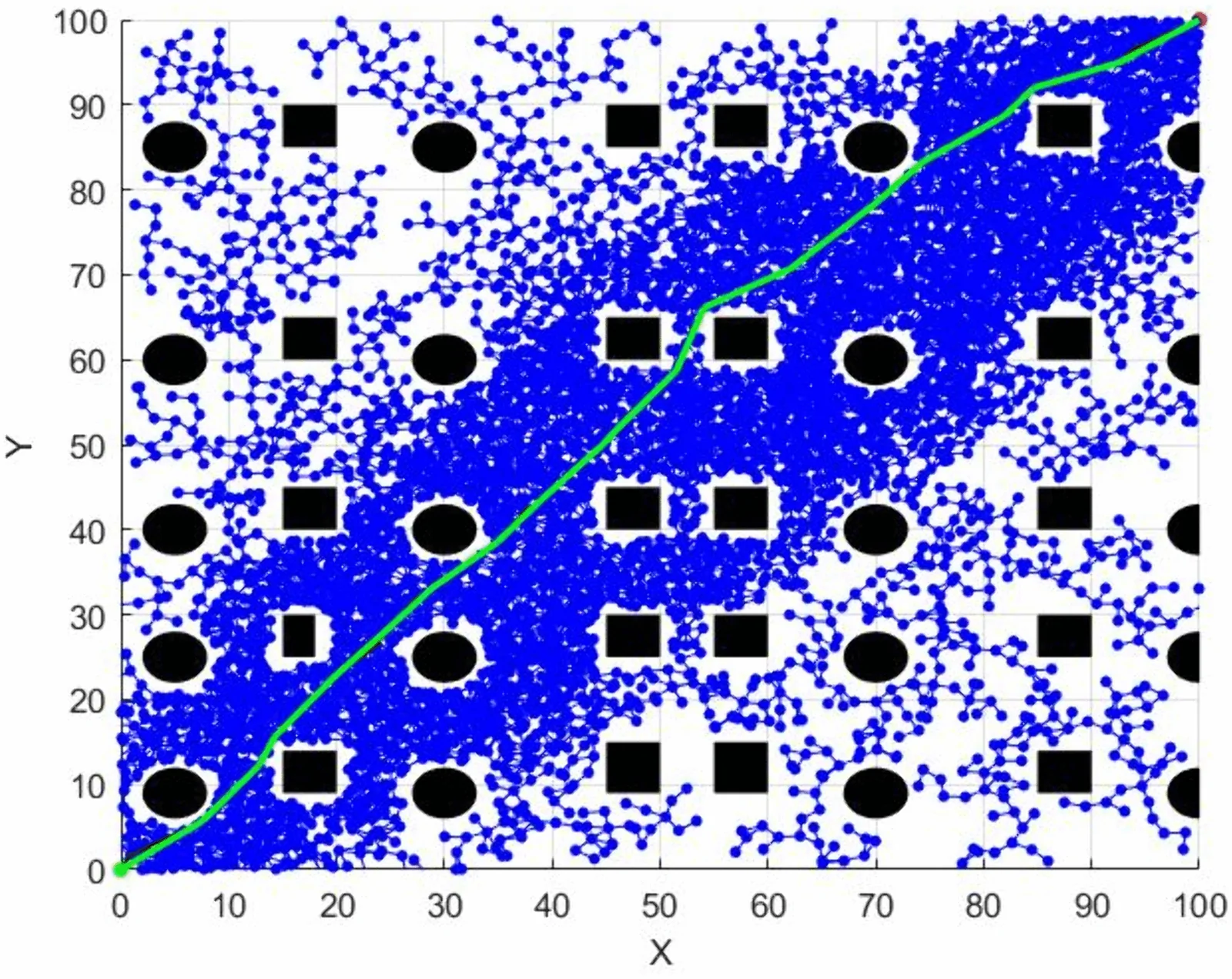
Results of running the five algorithms in Scene 2- Informed-RRT*.

**4.2.2.2 Performance in Scenario II (100 × 100 Normalized Grid):** In the medium-scale environment, ADBI-RRT continues to surpass all baseline algorithms in all critical indicators. The average computation time of ADBI-RRT is 4.47 s, 99.7% lower than that of Informed-RRT* (1240.76 s) and 96.9% lower than that of RRT* (146.19 s). The average number of sampling nodes (186.60 pc) is 89.5% fewer than that of RRT* (2674.00 pc) and 89.4% fewer than that of Informed-RRT* (7468.00 pc), further validating the efficiency of the target-biased sampling strategy. The average path length of ADBI-RRT is 145.35, 10.1% shorter than that of BI-RRT (161.59) and 9.1% shorter than that of APF-RRT* (160.03), confirming its capability to generate near-optimal paths. ADBI-RRT again achieves a 100% success rate, while the success rates of RRT* and Informed-RRT* are 95% and 96% respectively, highlighting its enhanced robustness. Figs 13–17 displays the path planning performance of the aforementioned five algorithms in Scene 2, the 100×100 normalized grid workspace (48 obstacles, 20% density, N = 10 independent trials per algorithm, with spatial axes representing normalized grid coordinates in the range x,y∈[0,100]. Start position is at (5, 5) and goal position is at (95, 95). As the map size and the number of obstacles increase, the morphological changes in the search process of the algorithms become more apparent. Performance statistics averaged over 10 trials are reported in Table 9. Conventional RRT* and Informed-RRT* generate dense blue sampling nodes covering most of the space. In contrast, the pink sampling nodes of ADBI-RRT are highly convergent and closely distributed within the effective connection area between the start and goal points. This indicates that the ADBI-RRT algorithm can effectively suppress the exploration of invalid spaces, improving the directivity of planning while maintaining optimization capabilities.

**Fig 18:**
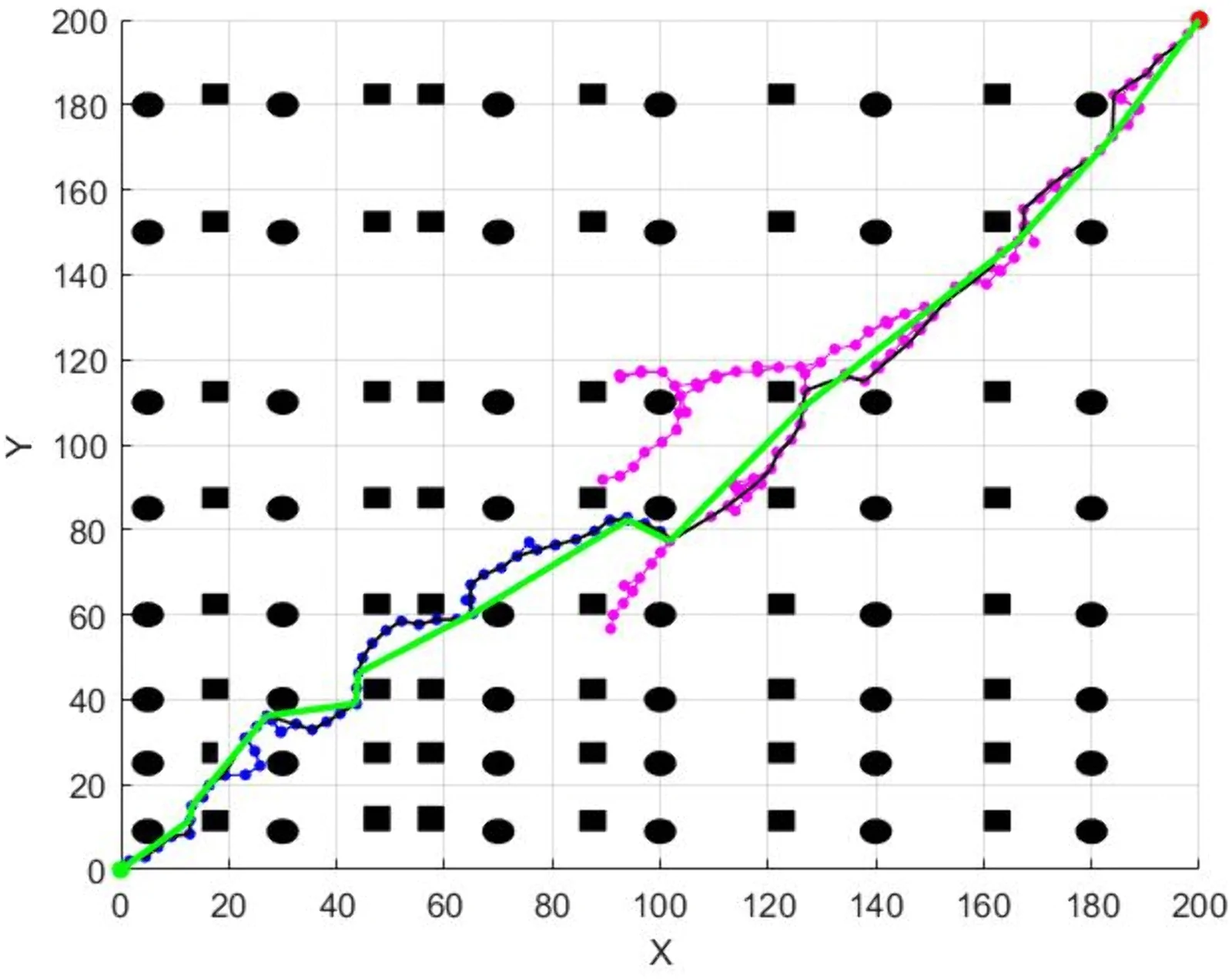
Results of running the five algorithms in Scene 3- ADBl-RRT.

**Fig 19:**
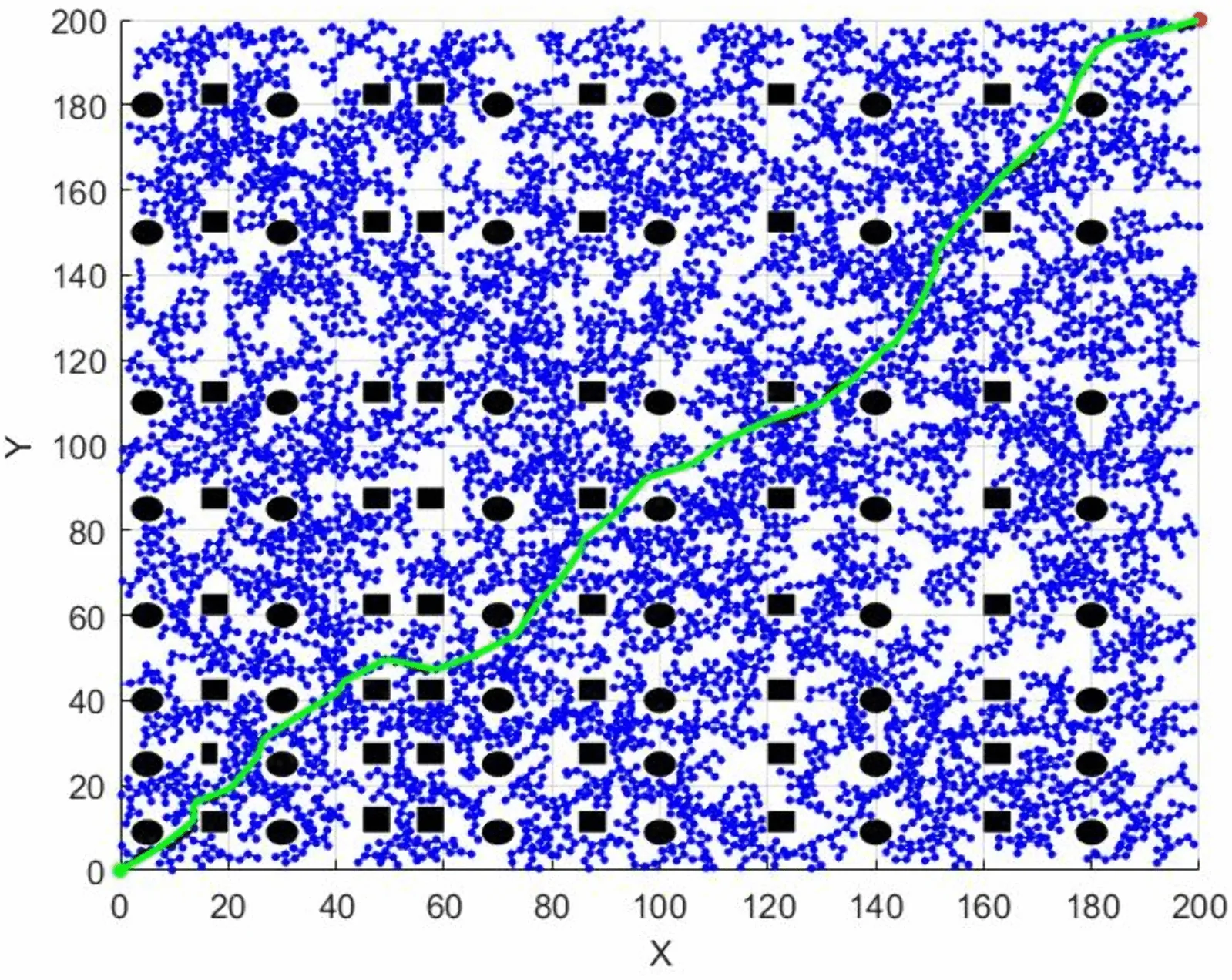
Results of running the five algorithms in Scene 3- RRT*.

**Fig 20:**
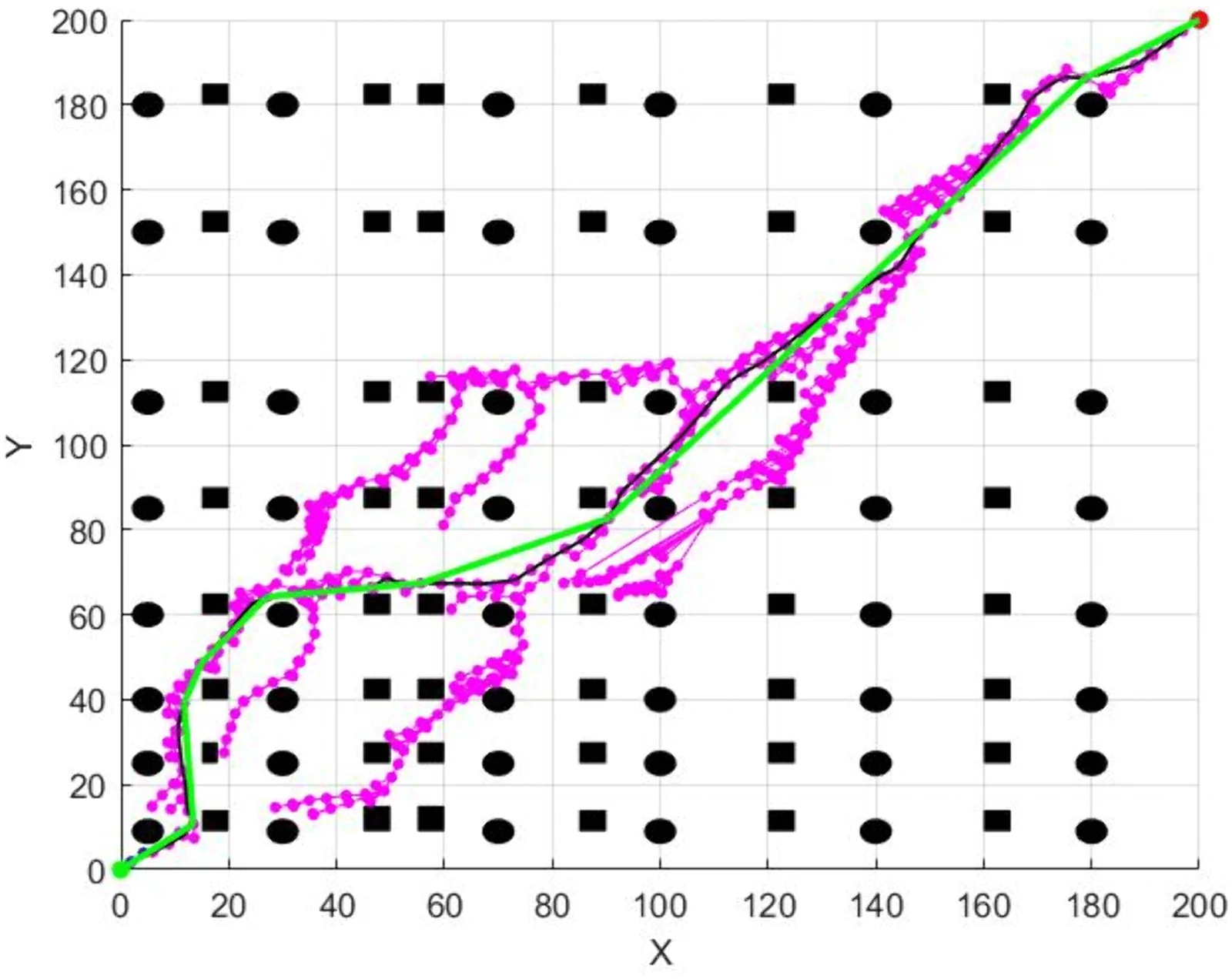
Results of running the five algorithms in Scene 3- Bl-RRT.

**Fig 21:**
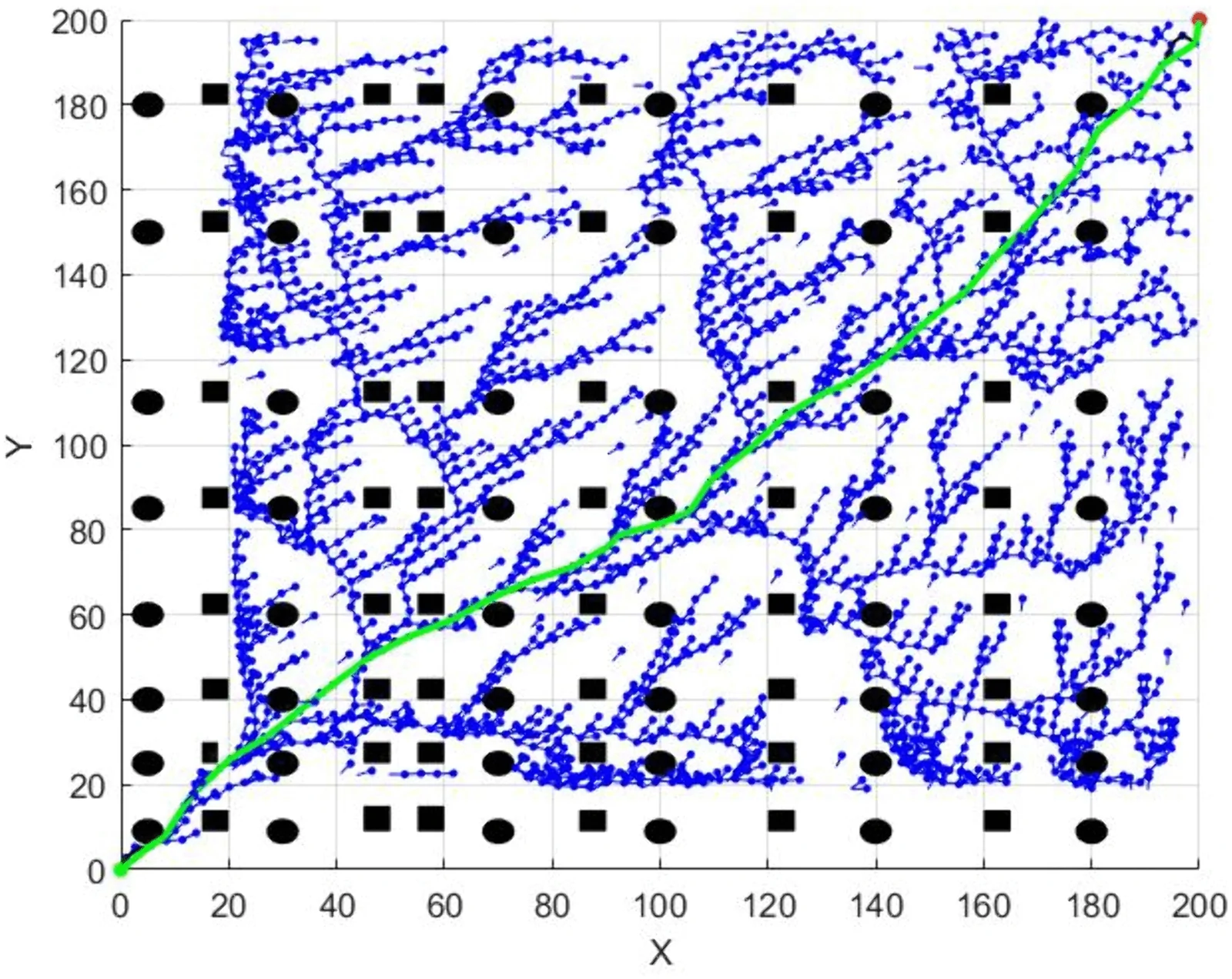
Results of running the five algorithms in Scene 3- APF-RRT*.

**Fig 22:**
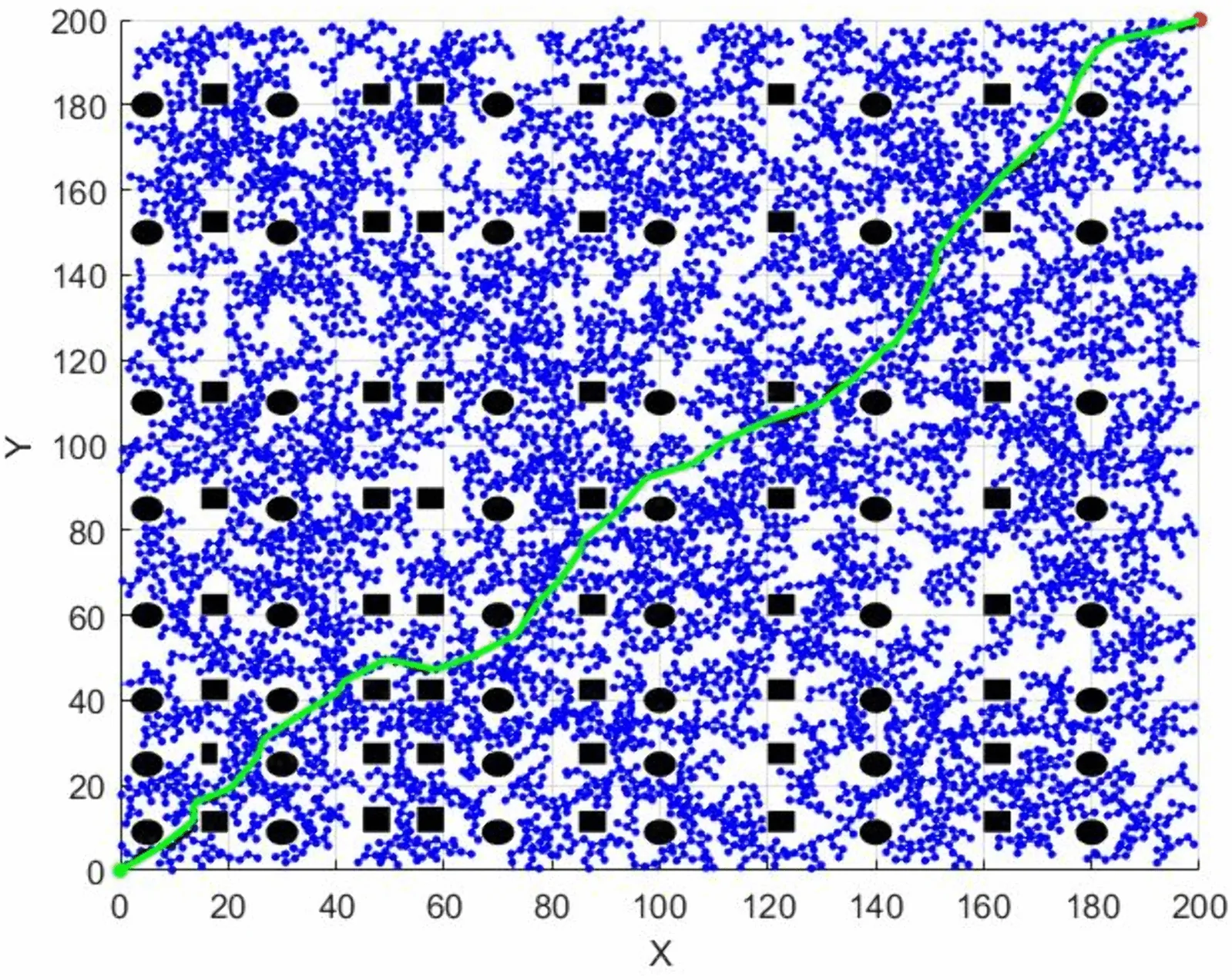
Results of running the five algorithms in Scene 3- Informed-RRT*.

**4.2.2.3 Performance in Scenario III (200× × 200 Normalized Grid):** Figs 18–22 illustrates the path planning results in Scene 3, the 200×200 normalized grid workspace (96 obstacles, 25% density, N = 10 independent trials per algorithm, with spatial axes representing normalized grid coordinates in the range x,y∈[0,200]. Start position is at (5, 5) and goal position is at (195, 195). The performance comparison across all three environments demonstrates the scalability of the proposed algorithm.

In the largest and most complex environment, ADBI-RRT retains its leading performance despite the increased difficulty of obstacle avoidance. The average computation time of ADBI-RRT is 7.88 s, 98.8% lower than that of Informed-RRT* (666.38 s) and 88.4% lower than that of RRT* (71.47 s). The average number of sampling nodes (286.20 pc) is 91.9% fewer than that of APF-RRT* (4009.00 pc) and 91.5% fewer than that of Informed-RRT* (3440.50 pc), underscoring its ability to minimize redundant exploration. The average path length of ADBI-RRT is 149.23, 5.4% shorter than that of BI-RRT (157.49) and 43.8% shorter than that of APF-RRT* (291.06), demonstrating consistent path optimization across different environmental scales. While ADBI-RRT achieves a 100% success rate, the success rates of RRT*, BI-RRT, and Informed-RRT* drop to 92%, 94%, and 91% respectively, further emphasizing the robustness of the proposed algorithm. Furthermore, the final green trajectory planned by this algorithm performs best in geometric smoothness during obstacle avoidance, demonstrating the notable performance improvements of ADBI-RRT within the evaluated large-scale complex scenarios.

#### 4.2.3 Limitation analysis of baseline path planning algorithms.

In contrast, Informed-RRT* and RRT* generate a large number of redundant sampling nodes, approximately 2.1 times and 2.8 times that of ADBI-RRT, respectively. ... with an average path length 18–25% longer than that of ADBI-RRT and relatively poor path quality. This is because the bidirectional tree expansion of BI-RRT adopts a near-termination iteration strategy, which frequently causes premature connection of the two trees before optimal path segments are discovered. APF-RRT* is prone to falling into local minima in dense obstacle clusters, which was observed in 3 out of 10 tests in the 200×200 environment with 25% obstacle coverage. The main cause is that the artificial potential field component fails to provide effective guidance for the robot to escape from high-density obstacle regions. The quantitative and qualitative experimental results of the five algorithms in Scenario 1 (50×50), Scenario 2 (100×100), and Scenario 3 (200×200) are presented in Figs 8–22, respectively.

#### 4.2.4 Quantitative performance summary across three environments.

Table 9 presents comparative performance across three environmental configurations in MATLAB simulation. In Setting I (50×50, 12 obstacles), ADBI-RRT achieves 1.83 s computation time compared to RRT*’s 51.92 s, representing a 96.47% reduction. The algorithm generates 73.40 nodes versus RRT*’s 638.10 nodes (88.50% reduction) while producing a 29.33 dimensionless path length compared to 74.71 (60.75% reduction). Setting II (100×100, 48 obstacles) demonstrates 4.47 s computation time versus RRT*’s 146.19 s (96.94% reduction), with node counts of 186.60 versus 2674.00 (93.02% reduction). Setting III (200×200, 96 obstacles) shows 7.88 s versus 71.47 s (88.97% reduction) and 286.20 nodes versus 2645.50 (89.18% reduction). The algorithm maintains 100% success rate across all settings, while RRT* success rates decline from 98% to 92% as environmental complexity increases.

#### 4.2.5 Validation of dynamic obstacle avoidance for the proposed fusion algorithm.

**Fig 23:**
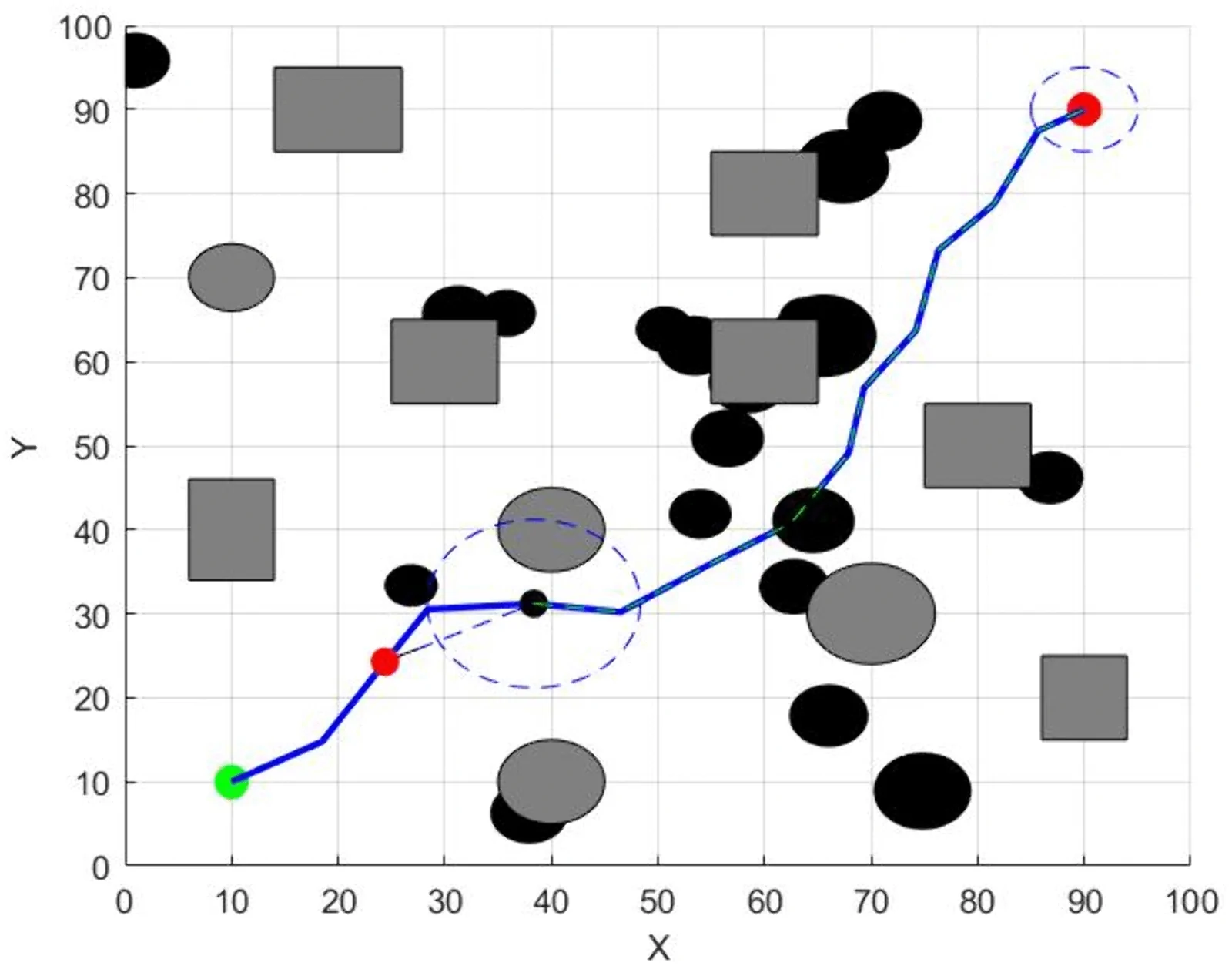
Initial Trajectory of Mobile Robot in Dynamic Environment.

**Fig 24:**
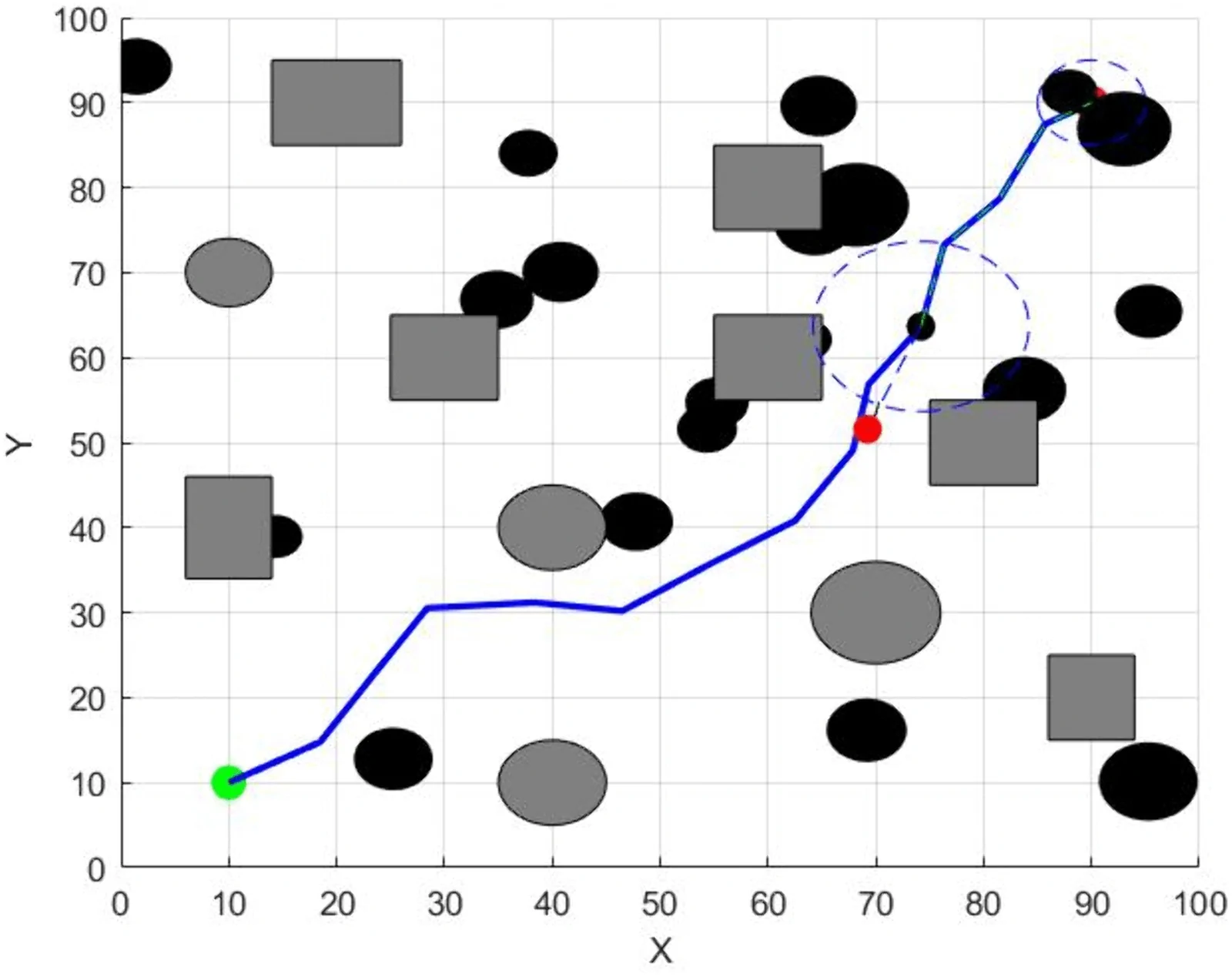
Obstacle Avoidance Trajectory of Mobile Robot in Dynamic Environment.

**Fig 25:**
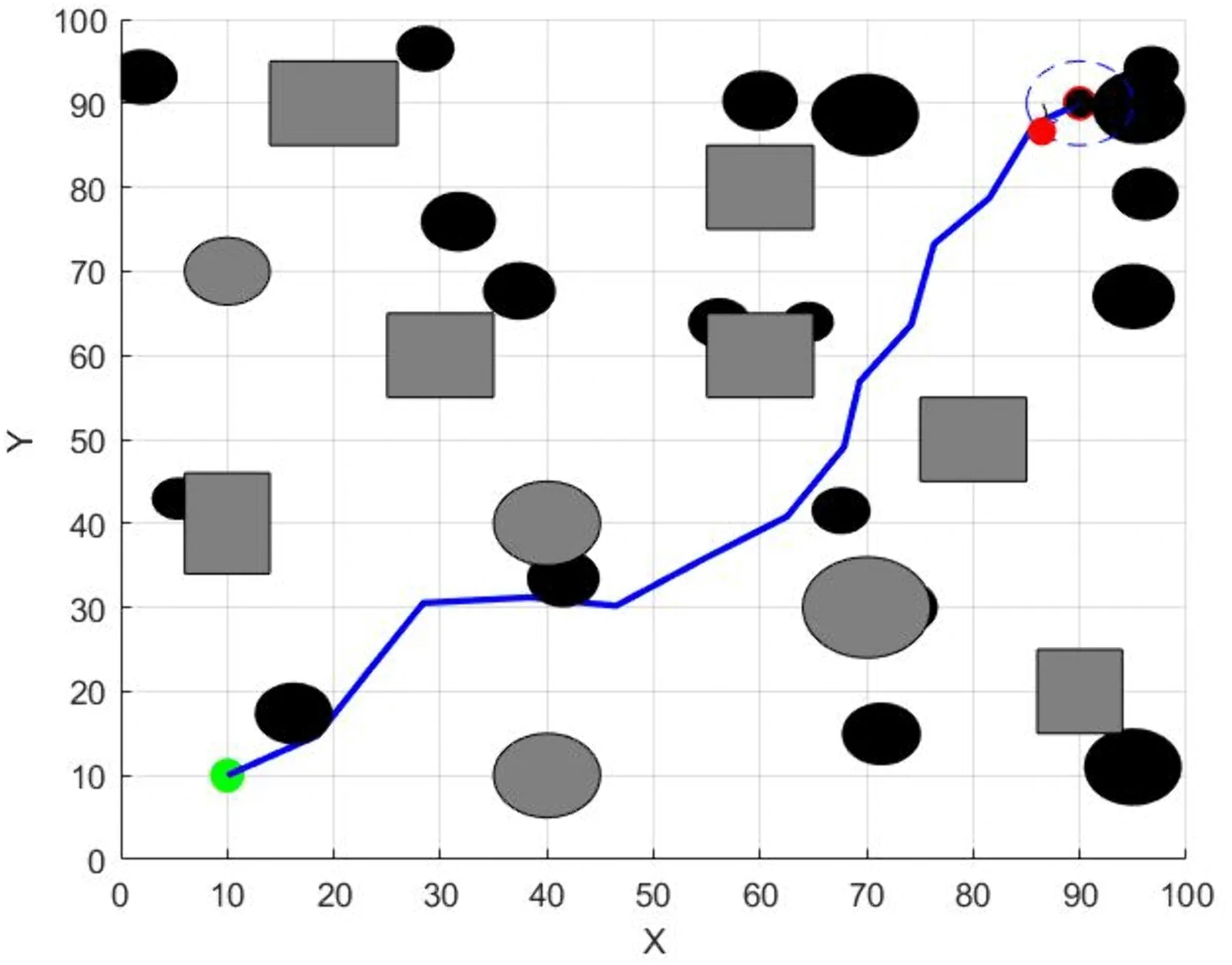
Successful Goal Reaching for Mobile Robot in Dynamic Environment.

Furthermore, fusion experiments were conducted by combining ADBI-RRT (as the global path planner) with the enhanced DWA algorithm (as the local motion controller), and the dynamic obstacle avoidance performance is illustrated in Figs 23–25, which present sequential snapshots in a 100×100 normalized grid workspace with 20 dynamic obstacles (radius 1.5 grid units, velocities 0.3–0.7 grid units/s) and multiple static obstacles (gray rectangles), where spatial axes represent normalized grid coordinates and N = 20 independent trials all achieved successful collision-free navigation.

**4.2.5.1 Experimental configuration for dynamic obstacle avoidance:** The dynamic obstacle prediction experiments were conducted in MATLAB simulation with two configurations to validate the robustness and scalability of the proposed strategy:

**Standard Configuration:** A 100×100 normalized grid workspace containing 10 dynamic obstacles (black circular obstacles, radius 0.3 grid units) and multiple static obstacles (gray rectangular obstacles, size 2×2 grid units). The dynamic obstacles move along random linear trajectories with velocities uniformly distributed in the range 0.4–0.6 grid units/s and accelerations in [−0.2, + 0.2] grid units/s^2^. This configuration represents typical operational scenarios with moderate obstacle density.

**High-Density Configuration:** To rigorously test the enhanced DWA’s dynamic obstacle avoidance capability, a challenging scenario was designed with 20 dynamic obstacles concentrated in the central corridor region of the workspace. Unlike the standard configuration where obstacles are uniformly distributed, this setup deliberately places the majority of moving obstacles along the robot’s planned path, creating a dense dynamic zone that the robot must traverse to reach the goal. Each dynamic obstacle follows a more complex motion model with time-varying velocities (0.3–0.7/s) and non-uniform accelerations ([−0.3, + 0.3]/s^2^), including periodic direction changes every 2–3 seconds. This configuration tests three critical aspects: (1) the DWA’s ability to handle multiple simultaneous collision threats in confined spaces, (2) the effectiveness of the dynamic obstacle prediction mechanism when obstacles exhibit non-linear motion patterns, and (3) the historical trajectory consistency term’s role in maintaining smooth navigation through dense obstacle clusters. The static obstacles (gray rectangles) remain at fixed positions throughout the navigation process.

The distinction between obstacle types is maintained consistently across all experiments: black circular obstacles represent dynamic entities with time-varying positions, while gray rectangular obstacles represent static environmental structures.

**4.2.5.2 Performance results:** MATLAB simulation results indicate that the mobile robot successfully tracks the globally optimal path generated by ADBI-RRT, with a path tracking error within 0.3 grid units. The robot dynamically adjusts its linear and angular velocities through the enhanced DWA algorithm to avoid moving obstacles in real time, with a response latency controlled within 0.05 s.

In the standard configuration with 10 dynamic obstacles, the DWA computation time averaged 0.045–0.05 s per cycle, corresponding to a control frequency of 20 Hz. In the high-density configuration with 20 dynamic obstacles concentrated in the central corridor, the computation time increased to 0.075–0.085 s per cycle, maintaining a control frequency above 12 Hz, which satisfies the real-time requirement of 10 Hz minimum. Despite the increased computational load, the robot successfully navigated through the dense dynamic zone without collisions in all 20 independent trials, reaching the goal position with a final pose error no more than 0.1.

The high-density configuration results demonstrate that the enhanced DWA maintains robust performance even when the majority of obstacles are positioned along the planned path. The dynamic obstacle prediction mechanism effectively anticipates future collision risks, while the historical trajectory consistency term prevents erratic maneuvers that could lead to instability in cluttered environments.

**4.2.5.3 Trajectory evolution in complex dynamic environments:** Building upon the standard dynamic tests, the high-density scenario provides a rigorous validation of the proposed algorithm under stringent spatial constraints. The sequential trajectory evolution is presented in Figs 23–25, showing trajectory snapshots at different time instances in the 100×100 normalized grid workspace, where gray shapes represent static structural constraints and black circles represent dynamic obstacles (with dashed blue circles denoting their predictive safety margins). The blue solid line represents the executed robot trajectory, and the yellow dashed line indicates the global ADBI-RRT waypoint sequence, with all axes in normalized grid coordinates.

As illustrated in Fig 23, during the initial phase, the mobile robot (green start point) detects multiple incoming dynamic obstacles concentrated along its planned route. Guided by the dynamic obstacle prediction cost in the enhanced DWA evaluation function, the local controller proactively adjusts the velocity commands to maintain safe distances from the moving entities while progressing toward the goal.

Fig 24 depicts the navigation process through the central corridor, which contains the highest concentration of dynamic obstacles. In this region, the robot must simultaneously avoid up to 8 moving obstacles within a 30×30 area while maintaining forward progress. The historical trajectory consistency weight prevents abrupt heading changes, ensuring that the executed path (blue solid line) remains kinematically feasible despite the dense obstacle environment.

Fig 25 confirms the successful traversal of the dense dynamic zone and the final convergence to the goal point (red dot). The continuous trajectory demonstrates that the global ADBI-RRT waypoints provide effective guidance through the obstacle field, while the enhanced DWA resolves short-term collision threats through predictive avoidance maneuvers. This sequential experiment validates the fusion framework’s capability to maintain reliable navigation performance in complex, non-deterministic environments with concentrated dynamic obstacles.

#### 4.2.6 Comprehensive performance analysis and algorithmic scalability.

To systematically evaluate the efficacy of the proposed framework, a quantitative analysis based on the statistical data in Table 9 was conducted. Rather than evaluating isolated metrics, the analysis focuses on the algorithm’s scalability and its behavioral shifts across environments of varying complexity (Settings I to III).

**4.2.6.1 Scalability and local minima mitigation:** Table 9 shows the computational trajectory of the algorithms as the spatial scale expands. For instance, while APF-RRT* exhibits a competitive computation time of 15.15 s in the relatively simple 50×50 environment (Setting I), its time consumption surges to 249.24 s in the 200×200 environment (Setting III). This non-linear degradation exposes the inherent vulnerability of conventional APF methods to local minima in dense, large-scale obstacle clusters. In contrast, ADBI-RRT maintains a highly stable computational profile, scaling gracefully from 1.83 s to 7.88 s. This stability validates that the proposed dual-heuristic mechanism (combining target-point and conditionally activated random-point attractive forces) effectively prevents the algorithm from becoming trapped in localized penalty regions, ensuring consistent convergence regardless of map size.

**4.2.6.2 Sampling utility versus Brute-force exploration:** The data shows a clear difference in spatial exploration paradigms. Informed-RRT* restricts sampling within a heuristic ellipse, yet it consistently generates over 3400–7400 nodes across different settings, yielding path lengths between 75.23 and 157.89. ADBI-RRT, however, utilizes fewer than 300 nodes across all scenarios while consistently achieving the shortest path lengths (e.g., 29.33 in Setting I and 149.23 in Setting III). This indicates that the proposed multi-sampling scoring strategy shifts the paradigm from “quantity-driven random exploration” to “utility-driven selective expansion.” By simultaneously evaluating obstacle density and goal direction, ADBI-RRT ensures that almost every generated node contributes directly to the final optimal trajectory, virtually eliminating redundant memory allocation.

**4.2.6.3 Robustness under extreme constraints:** As the complexity of the environment increases in Setting III, the baseline algorithms (RRT*, BI-RRT, and Informed-RRT*) suffer noticeable performance drops, with success rates declining to a range of 91% to 94%. ADBI-RRT, conversely, is the only methodology that sustains a strict 100% success rate. Furthermore, its collision detection failure rate consistently stays at the lower bound (1.17% to 2.03%). This high level of reliability is not coincidental; it is a direct consequence of the dynamic step-size adjustment responding to local obstacle density. By enforcing smaller expansion steps in congested areas, the algorithm intrinsically guarantees higher resolution in collision checking exactly where safety margins are tightest, thereby maximizing overall navigation reliability.

### 4.3 Experimental validation

**Fig 26:**
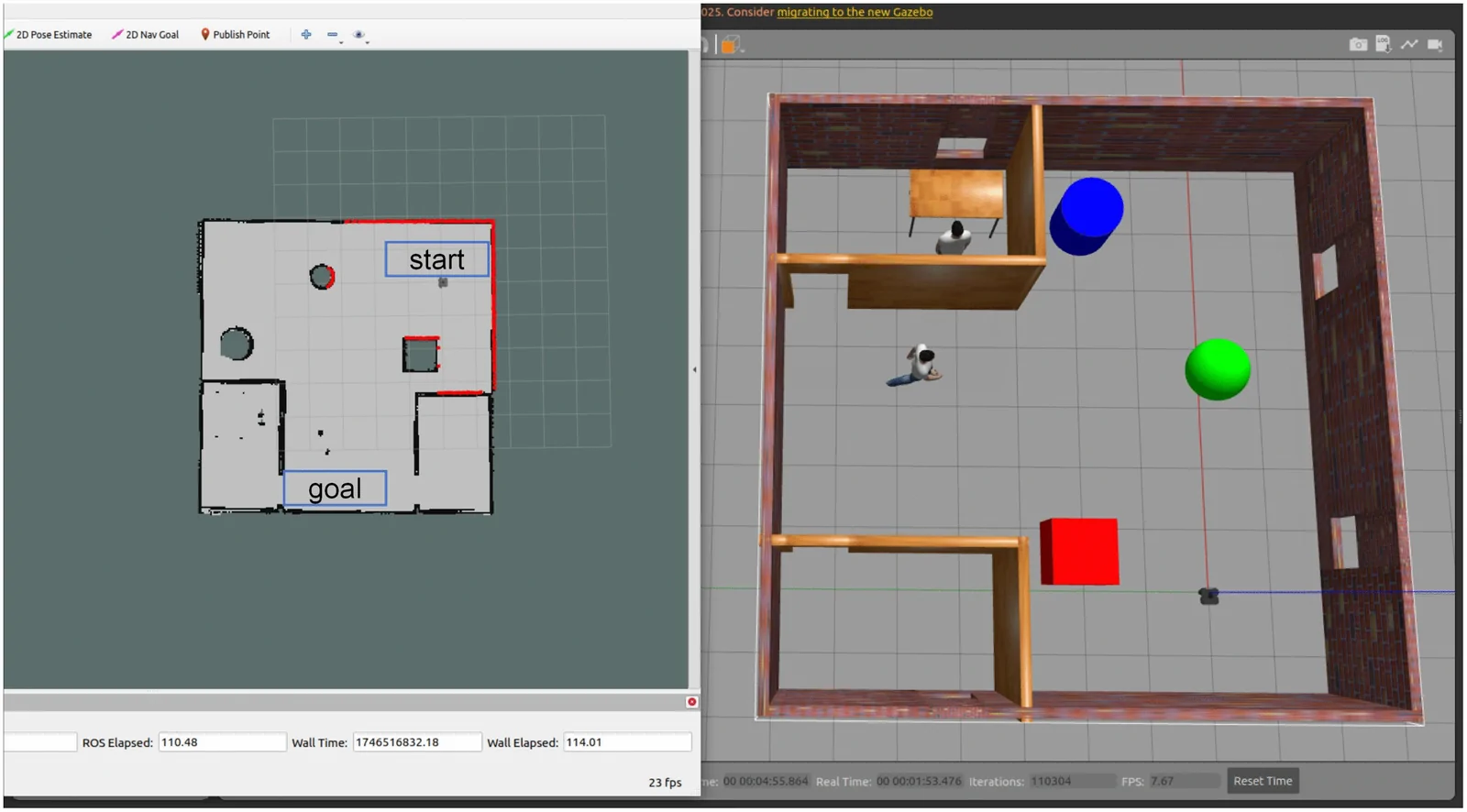
Experimental setup for environment initialization in the Gazebo simulator. The left panel displays the global navigation map with the predefined start and goal positions, while the right panel shows the corresponding 3D simulation scene in Gazebo with the robot spawned at the initial pose.

**Fig 27:**
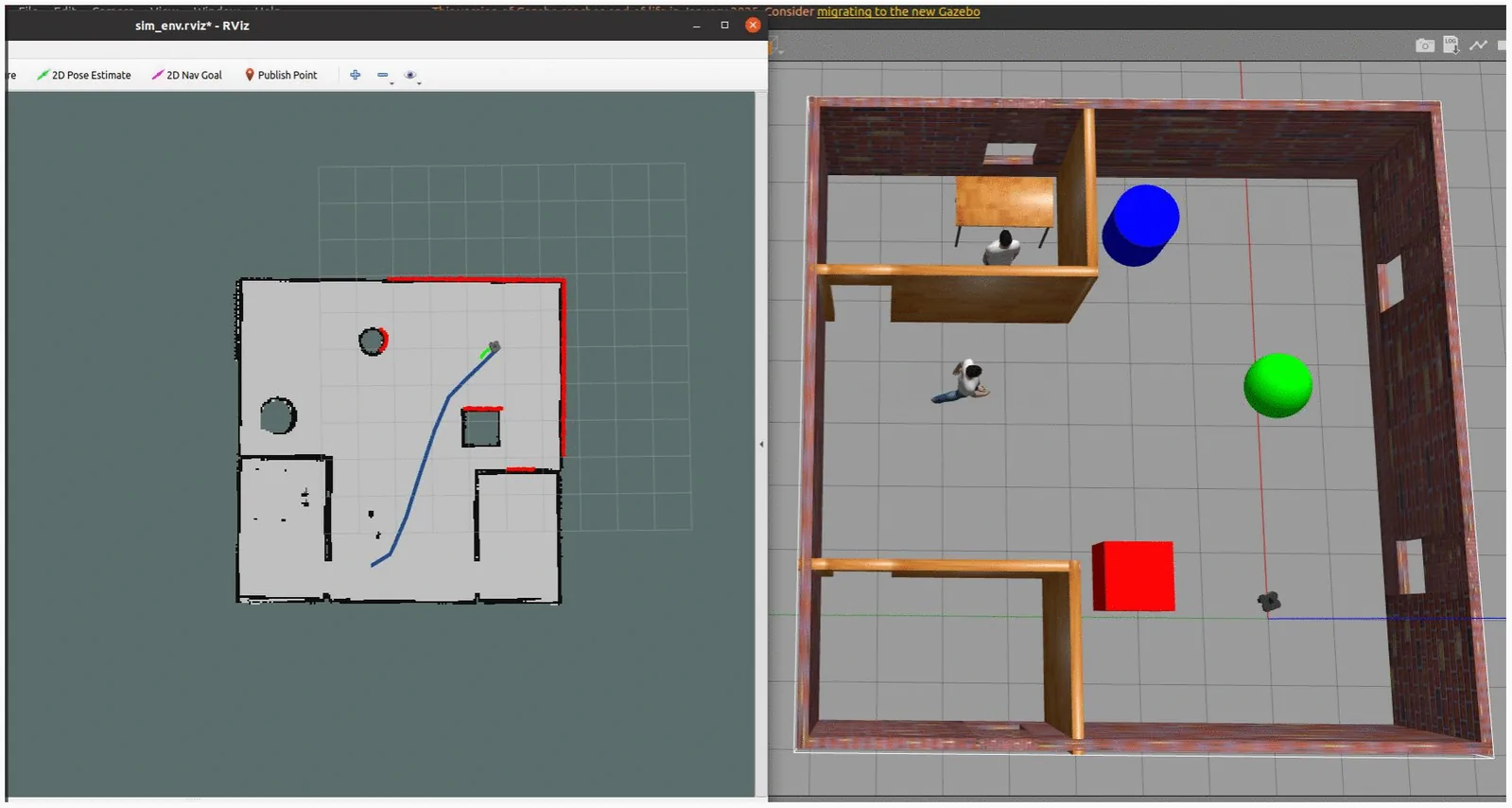
Global path (blue, ADBI-RRT) and local trajectory (green, enhanced DWA) in the Gazebo-RViz simulation.

**Fig 28:**
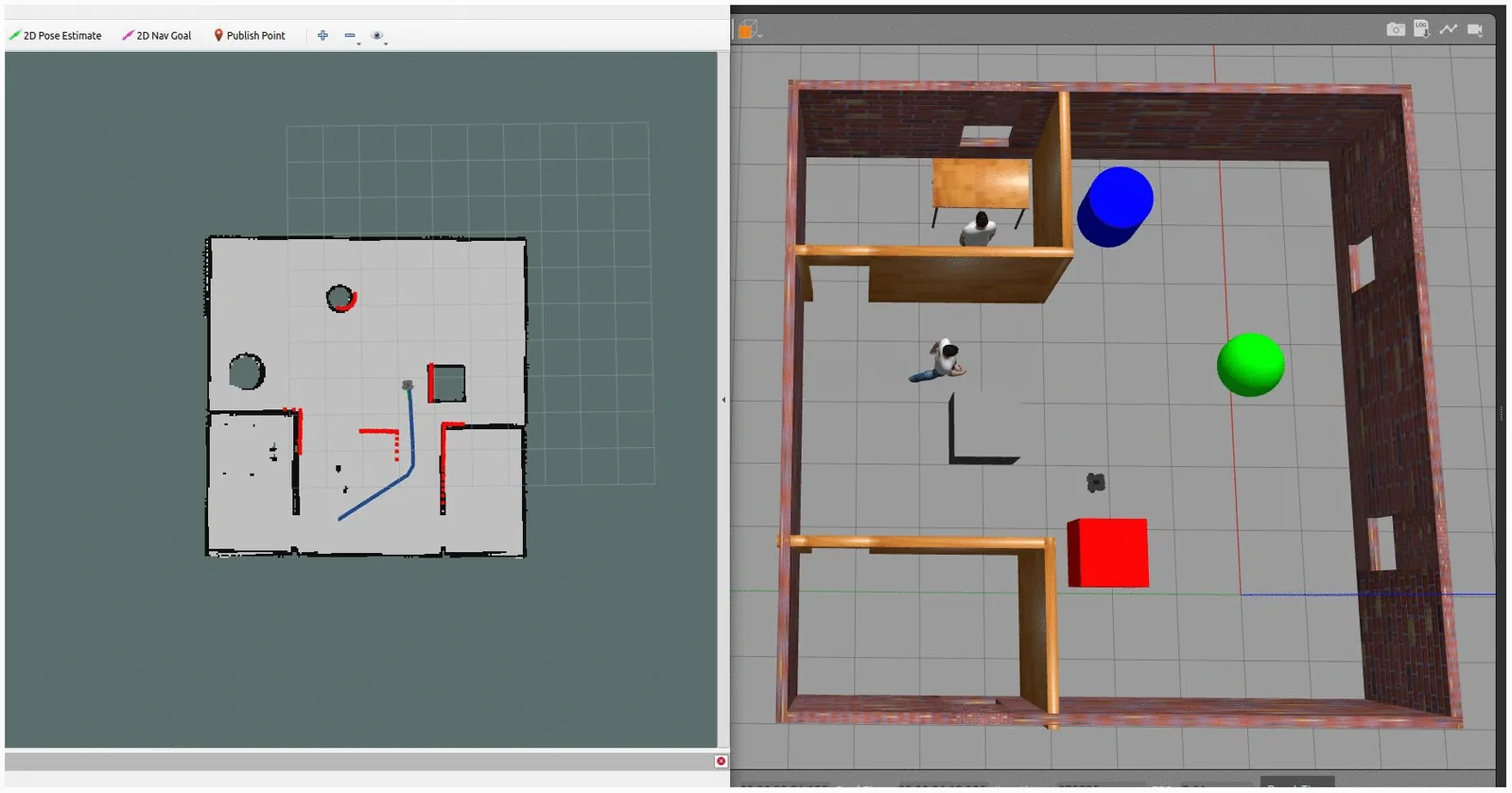
Real-time obstacle avoidance trajectory with local path adjustment in Gazebo.

**Fig 29:**
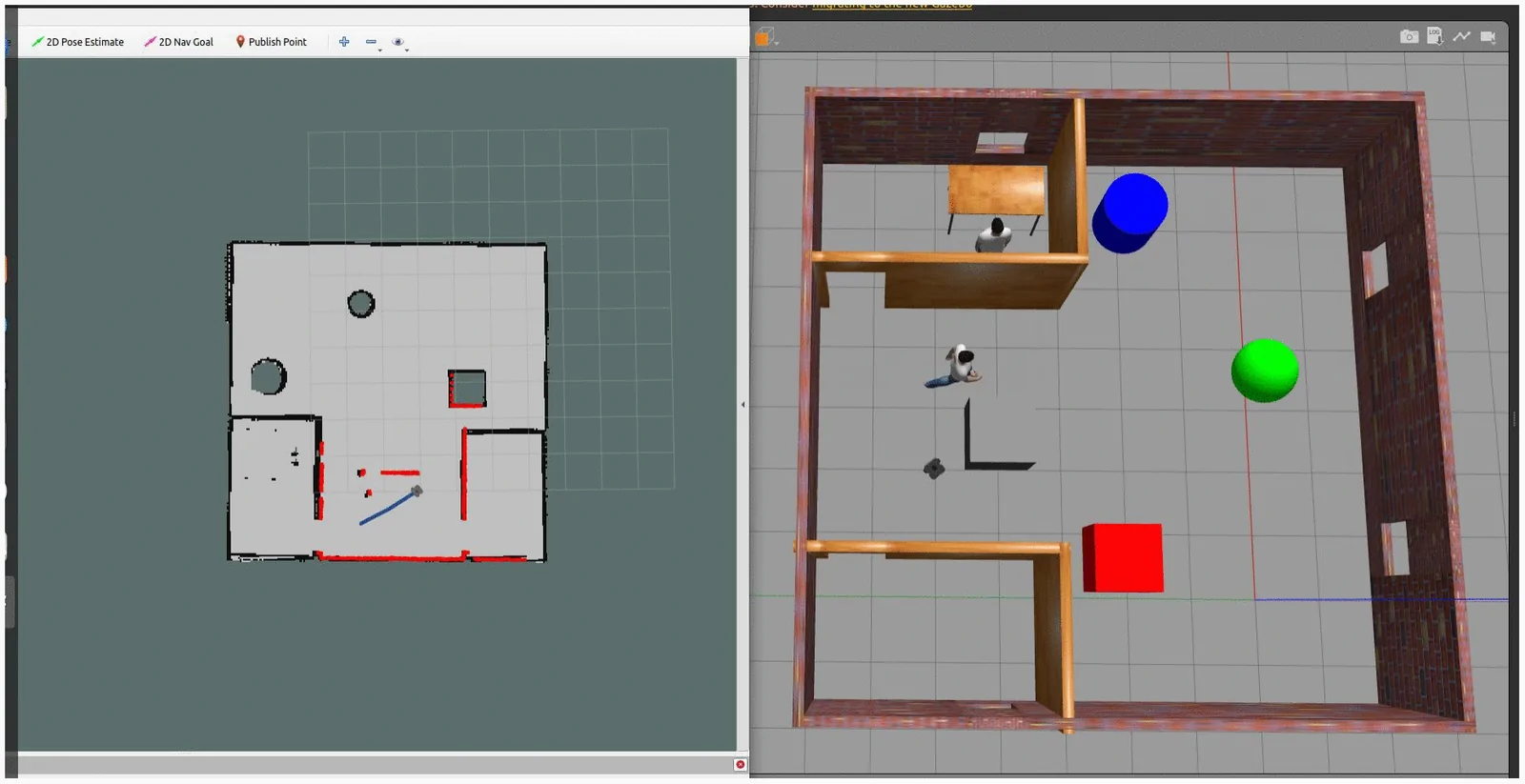
Successful obstacle avoidance trajectory in the Gazebo simulation.

**Fig 30:**
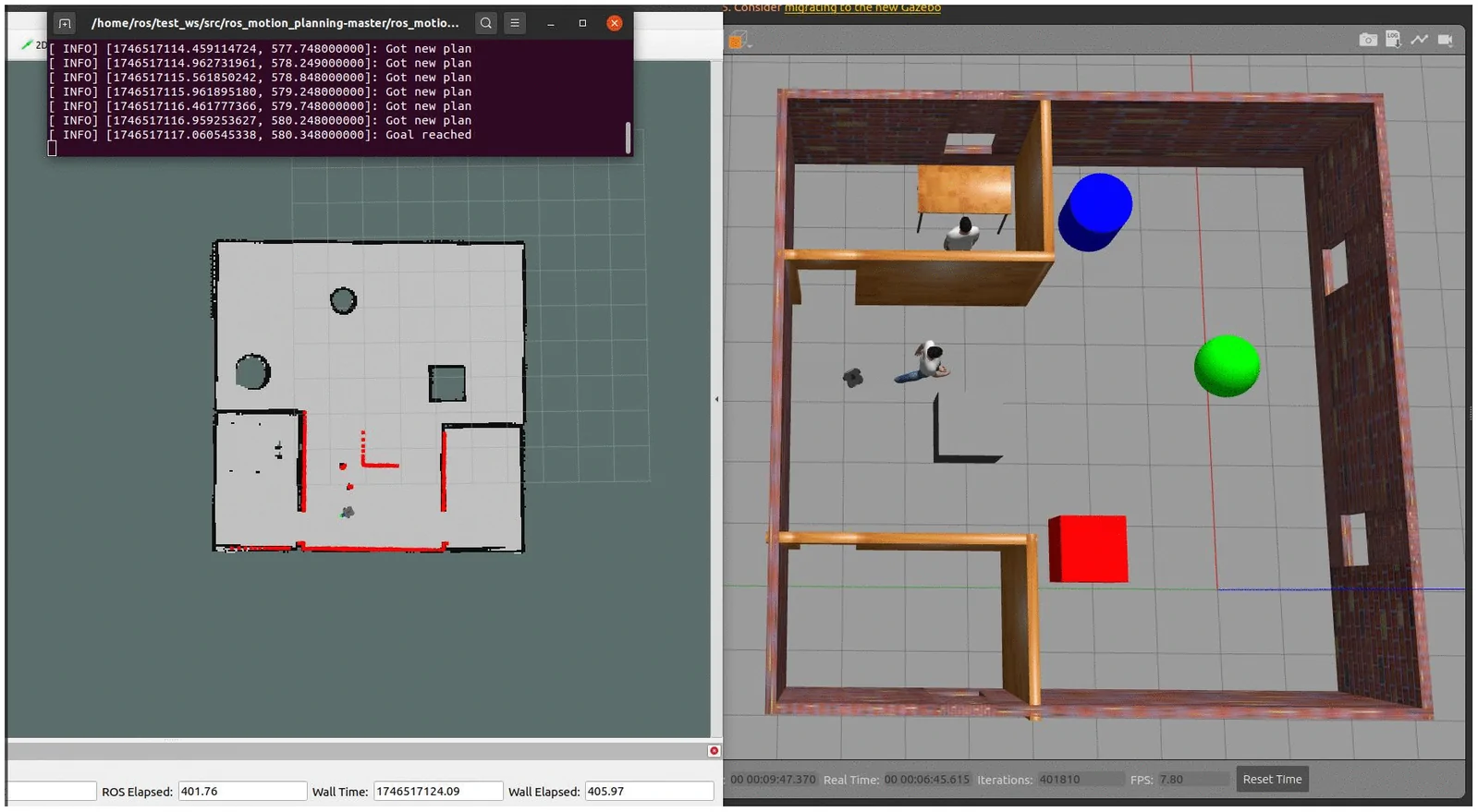
Terminal logs showing continuous local replanning until goal reached.

In this paper, simulations were conducted using Gazebo (Noetic version) on the Ubuntu 20.04 system. The initial position of the robot and the position of the target point are illustrated in Fig 26, which shows the simulated 8×8 meter indoor environment with 6 static cylindrical obstacles (diameter 0.5 m), where coordinate axes are in meters following ROS REP-103 standard. Fig 27 shows the robot’s initial path planning in this static environment, where the blue line represents the global path trajectory, and the green line indicates the local obstacle avoidance trajectory generated by the DWA algorithm, with spatial coordinates in meters. In Fig 28, an unknown obstacle is introduced to verify the obstacle avoidance performance of the improved DWA algorithm, with the figure showing real-time sensor data where coordinate axes are in meters (ROS world frame). As can be seen in Fig 28, the robot’s sensors have detected the unknown obstacle ahead, and the improved DWA initiates local obstacle avoidance. Fig 29 illustrates the successful bypass of the unknown obstacle, where the green trajectory line shows the smooth curvature of the avoidance maneuver with spatial coordinates in meters, and Fig 30 demonstrates that the robot has successfully reached the destination, which is further verified by the “Goal reached” message displayed in the terminal.

#### 4.3.1 ROS mobile robot experiment.

To validate the dynamic obstacle avoidance effect of the improved fusion algorithm proposed in this paper in real-world scenarios, the Jetauto mobile robot was used for testing.

The detailed hardware specifications of the Jetauto platform are listed in Table 10.

**Table 10:** Physical robot parameters of Jetauto platform.

Parameter	Specification
LiDAR	Slamtec RPLIDAR A1
Operating System	Ubuntu 18.04 LTS + ROS Melodic
Storage	32 GB TF card
Servo Model	HTS-20H intelligent bus servo
Motor Model	520 Hall encoder DC geared motor
Main Control Unit	Jetson Nano B01
Depth Camera	Orbbec Astra Pro
Battery Capacity	11.1 V 6000 mAh lithium battery
Endurance Time	60 min
SLAM Algorithm	Gmapping
Localization Algorithm	AMCL

**Fig 31:**
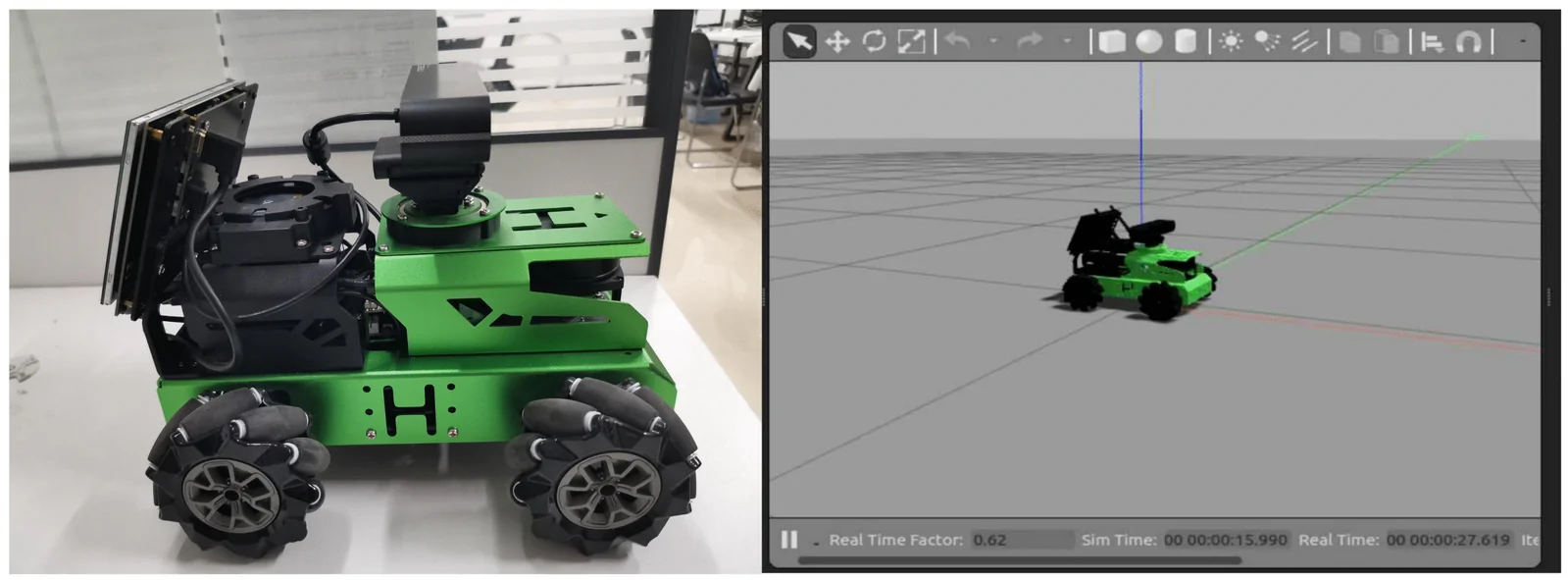
Jetauto robotic platform (left) and its Gazebo simulation model (right).

**Fig 32:**
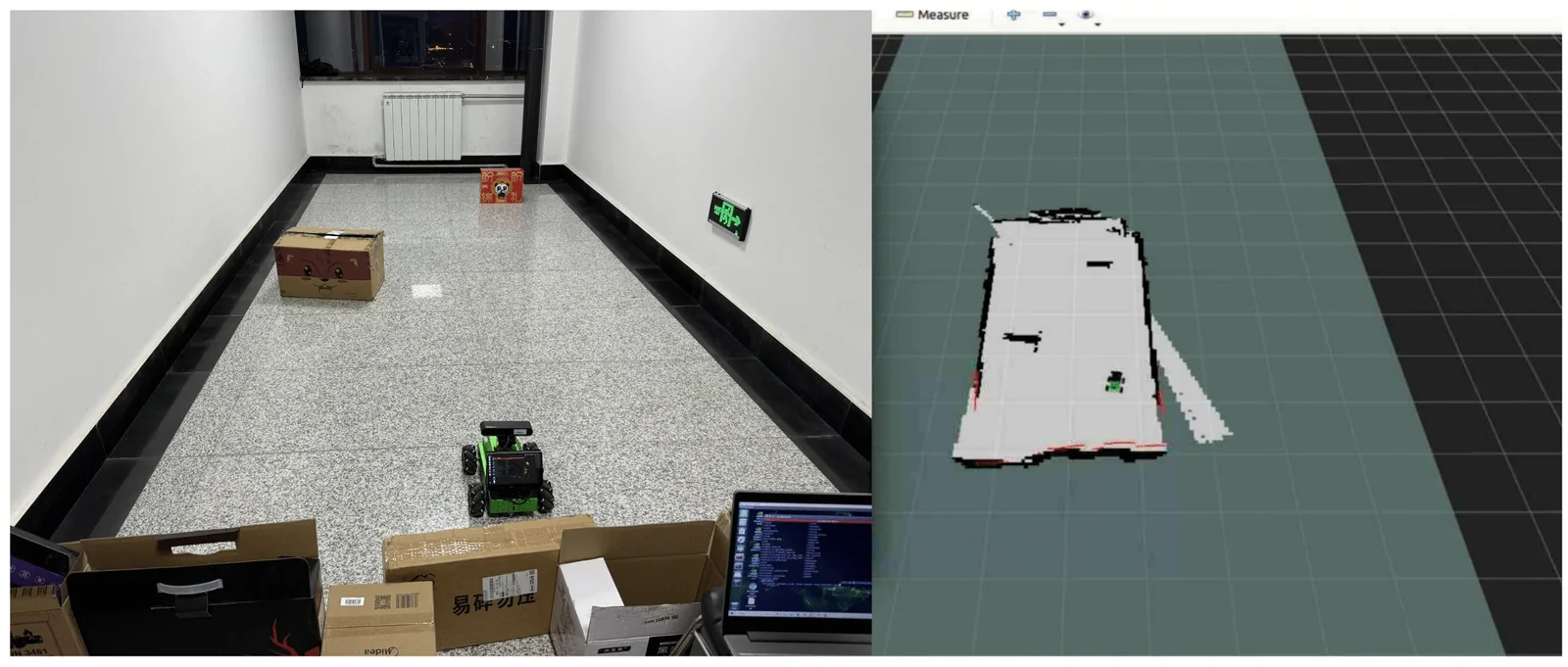
Jetauto robot path planning and obstacle avoidance experiment. Left: the physical test scene; Right: the corresponding raster map and localization in RViz.

The structure of the robot is illustrated in Fig 31, showing the Jetauto platform equipped with Slamtec RPLIDAR A1, Orbbec Astra Pro depth camera, and Jetson Nano controller. {The physical experiment was conducted in a room measuring 5.0 m in length and 2.2 m in width. Two static obstacles were placed in the environment: one measuring 60 × 50 cm and the other 40 × 30 cm. The overall layout of the Jetauto robot and the obstacles is illustrated in Fig 32, where the yellow line represents the ADBI-RRT global path and the blue line represents the DWA local trajectory, with coordinate axes in meters and origin at the room corner. The algorithm was implemented as an independent C++ ROS node, which subscribes to the /move_base_simple/goal topic to receive target poses and publishes the planned trajectory to the /path topic. The visualization in RViz shows the real-time navigation position and detected obstacles, with path colors corresponding to the global planner (yellow) and local controller.

**Fig 33:**
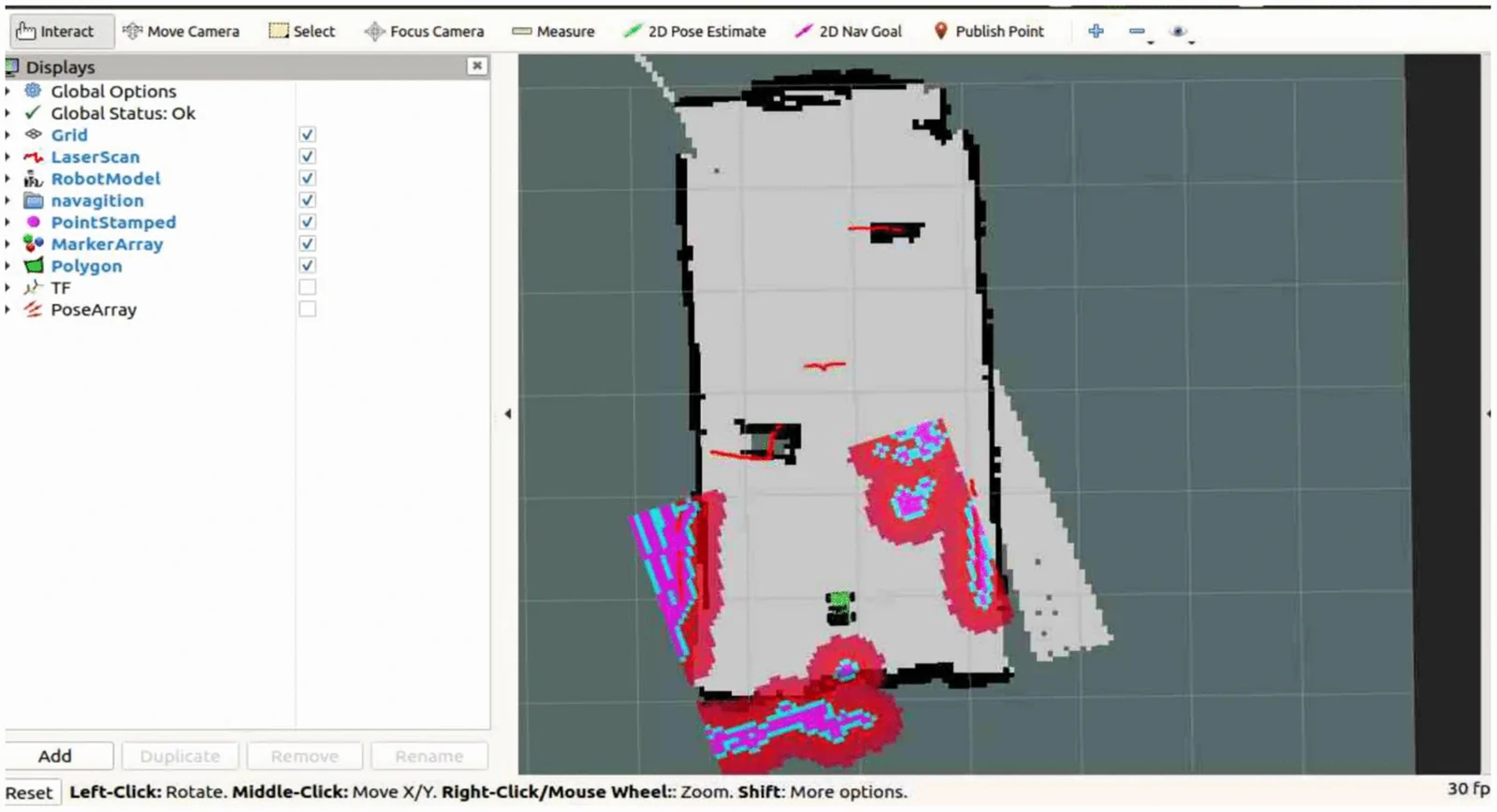
Experimental scene of the Jetauto robot navigating an indoor corridor with cardboard boxes for path planning and obstacle avoidance tests.

**Fig 34:**
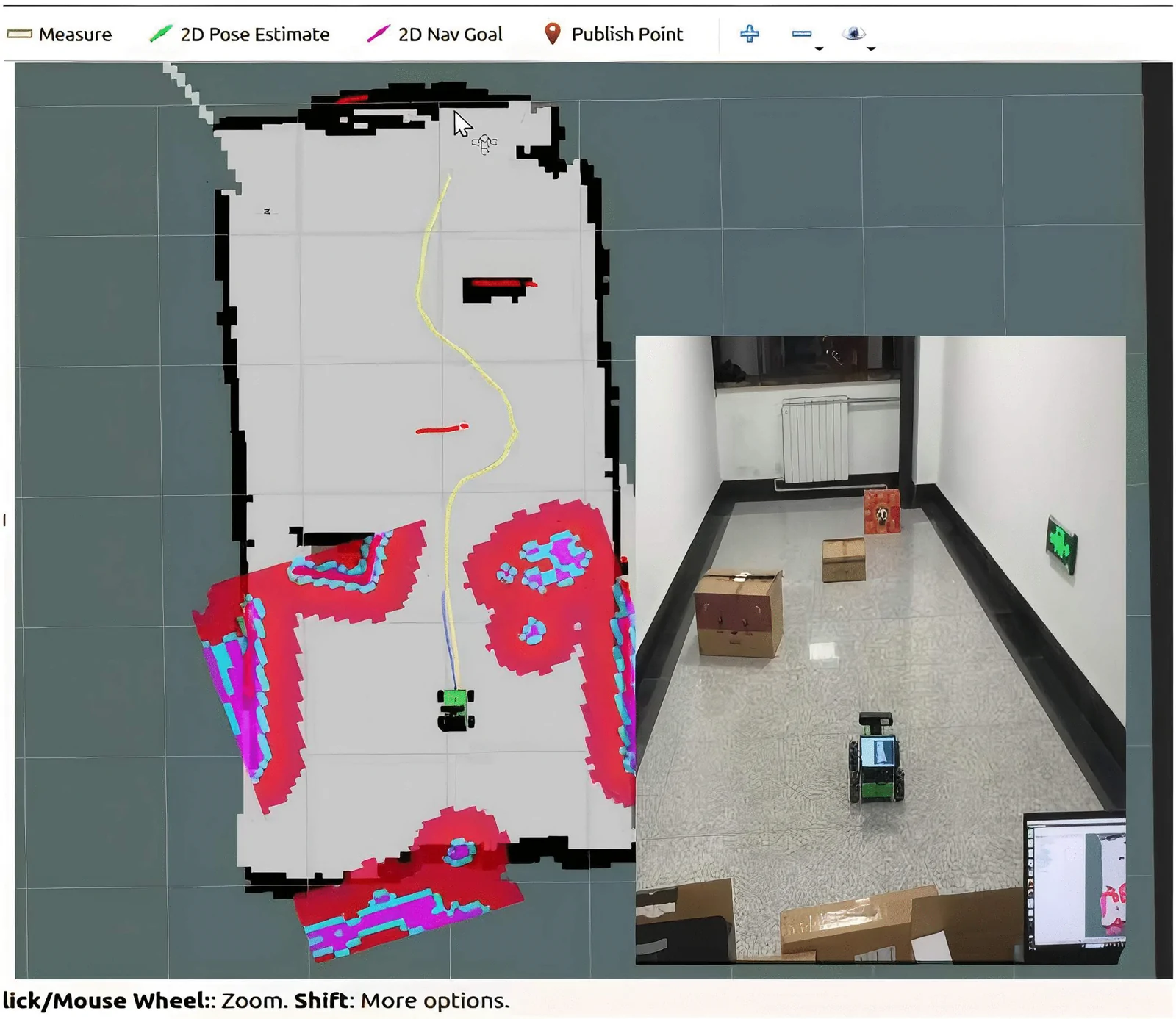
Global path (yellow, ADBI-RRT) and local trajectory (blue, enhanced DWA) in the RViz visualization.

**Fig 35:**
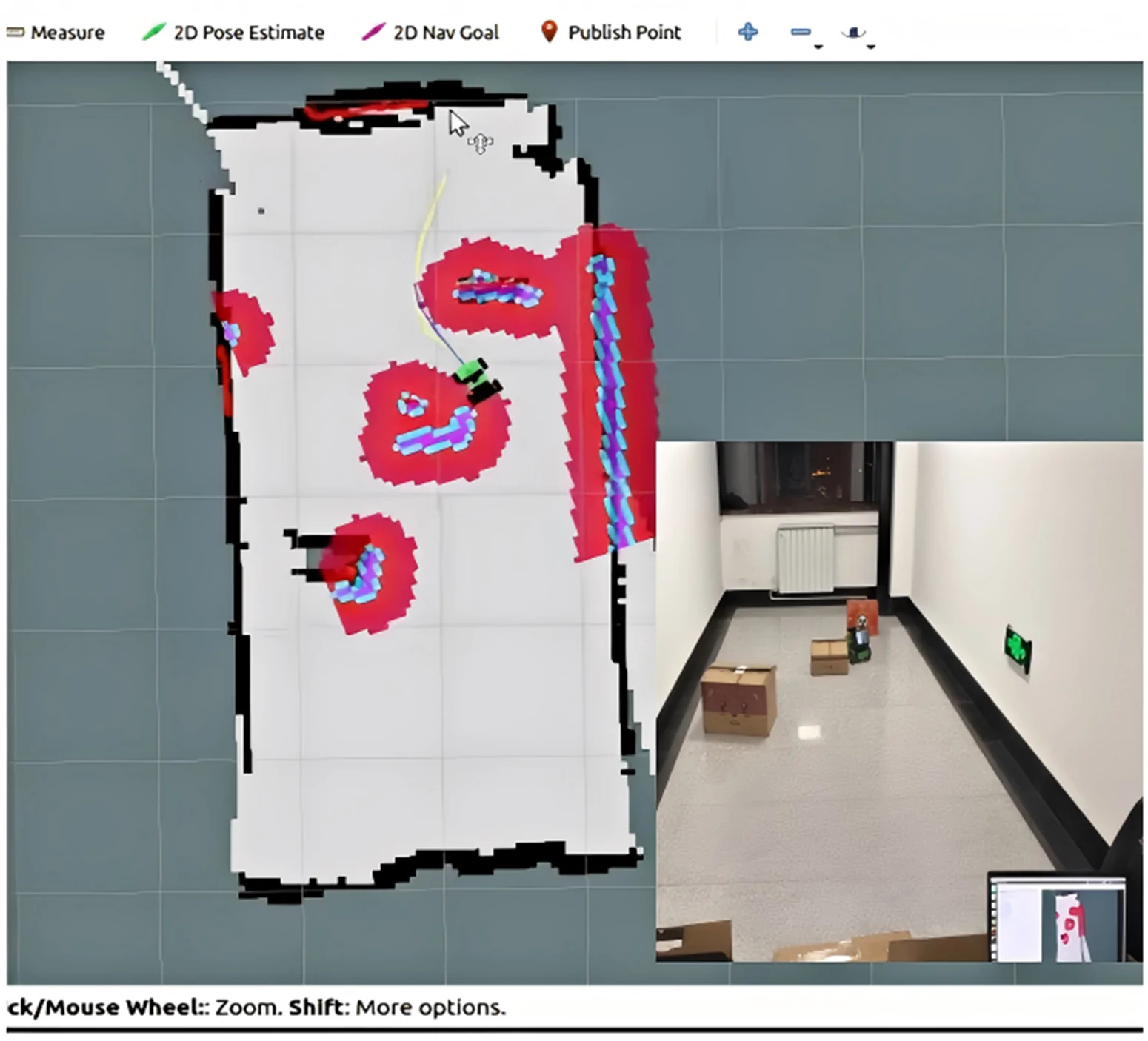
Obstacle avoidance trajectory of the mobile robot during forward motion in the RViz visualization.

**Fig 36:**
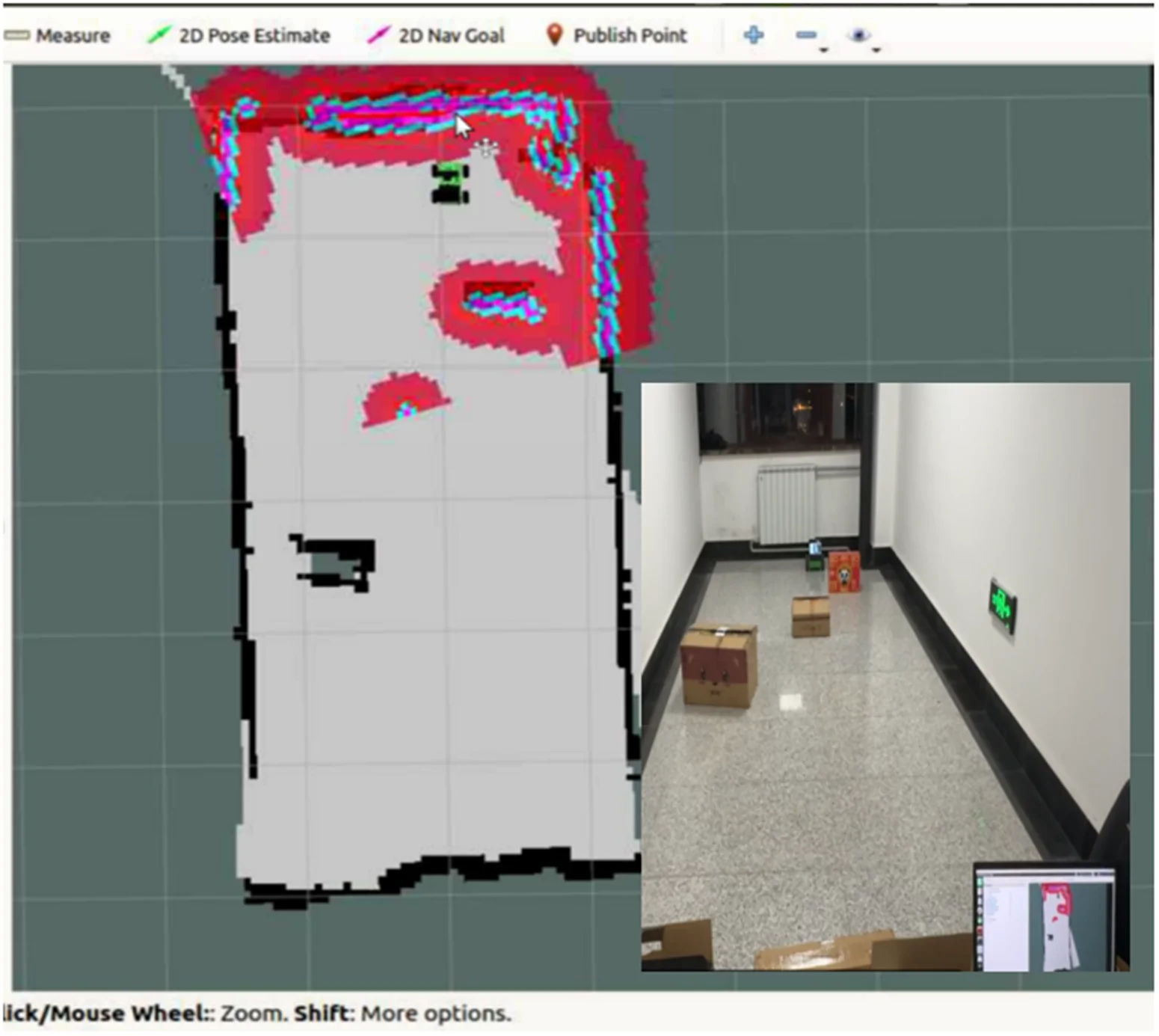
Successful goal arrival of the Jetauto robot in the RViz visualization.

Figs 33 and 34 mark the start position, end position, and local path, showing sequential snapshots of the navigation process where coordinate axes are in meters (room frame). These show that when the Jetauto encounters an obstacle, it replans the path and moves along the local path, with the blue line indicating the executed trajectory. Figs 35 and 36 demonstrate that the robot successfully avoids the obstacle and reaches the target point, indicating that the fusion algorithm achieves autonomous navigation with obstacle avoidance capability in unknown environments, with this single trial completed autonomously without human intervention.

A comparison was conducted between the improved fusion algorithm proposed in this paper and the traditional BI-RRT algorithm in terms of running time, path length, and number of turns during the autonomous navigation process. As can be obtained from Table 11, compared with the traditional BI-RRT algorithm, the improved fusion algorithm proposed in this paper shortens the path length by 8.17%, reduces the running time by 13.85%, and decreases the number of turns by 40%. It can be concluded that the improved fusion algorithm proposed in this paper generates a more optimal path.

**Table 11:** Comparison of planning algorithms in static obstacle avoidance during physical robot navigation (Jetauto platform, single trial).

Algorithm type	Path length (m)	Number of turns	Execution time (s)
Traditional BI-RRT + DWA	4.65	15	32.98
ADBI-RRT + Enhanced DWA (Proposed)	4.27	9	28.41

Physical robot experiments were conducted as single-trial demonstrations to validate real-world feasibility. Each trial was manually verified for path quality and collision avoidance performance. Statistical validation through multiple trials (N = 20) was conducted in MATLAB simulation (Tables 12–14), as physical robot experiments are time-intensive and resource-constrained.

### 4.4 Performance metrics across three environments (See Tables 12–14)

#### 4.4.1 Cross-environment performance analysis.

Table 15 consolidates performance metrics across the three test environments. Path length reductions vary with environmental scale: 60.75% in Environment 1, 8.73% in Environment 2, and 4.14% in Environment 3. The diminishing path length advantage in larger environments reflects the constraint that all algorithms must navigate similar geometric distances when obstacle density remains proportional to environment size. Computation time reductions remain consistently high across all scales (88.97–96.94%), with the maximum reduction occurring in Environment 2 (96.94%). This pattern indicates that the multi-sampling strategy and APF guidance mechanism provide stable computational efficiency regardless of environmental complexity. Node count reductions average 90.23%, with peak efficiency (93.02%) observed in Environment 2, suggesting optimal performance in medium-density obstacle configurations. The algorithm achieves 100% success rate across all environments, while baseline RRT* exhibits declining reliability (98% to 92%) as complexity increases. Collision detection failure rates for ADBI-RRT decrease with environmental scale (2.03% to 1.17%), indicating improved robustness in complex scenarios.

**Table 12:** Statistical analysis for Environment 1 (Simple, 50×50 normalized grid, 12 obstacles, N = 20 trials).

Algorithm	Path Length (dimensionless)		Time (s)		Nodes	
	Mean ± SD	95% CI	Mean ± SD	95% CI	Mean ± SD	95% CI
RRT*	74.71±3.15	[73.24, 76.18]	51.92±5.50	[49.42, 54.42]	638.10±85	[595, 681]
BI-RRT	81.30±2.80	[79.99, 82.61]	38.82±4.20	[36.62, 41.02]	99.40±15	[91, 108]
APF-RRT*	72.69±2.45	[71.50, 73.88]	15.15±1.95	[14.20, 16.10]	678.10±90	[633, 723]
Informed-RRT*	75.23±2.10	[74.25, 76.21]	1223.67±120.00	[1163.67, 1283.67]	7060.00±850	[6635, 7485]
**ADBI-RRT**	29.33±0.85	**[28.93, 29.73]**	1.83±0.20	**[1.74, 1.92]**	73.40±10	**[68, 79]**
***Coefficient of Variation (CV, %)***						
RRT*	4.22		10.59		13.32	
BI-RRT	3.44		10.82		15.09	
APF-RRT*	3.37		12.87		13.27	
Informed-RRT*	2.79		9.81		12.04	
**ADBI-RRT**	**2.90**		**10.93**		**13.62**	

Bold values indicate best performance. CV = Coefficient of Variation.

**Table 13:** Statistical analysis for Environment 2 (Medium, 100×100 normalized grid, 48 obstacles, N = 20 trials).

Algorithm	Path Length (dimensionless)		Time (s)		Nodes	
	Mean ± SD	95% CI	Mean ± SD	95% CI	Mean ± SD	95% CI
RRT*	159.26±5.85	[156.53, 162.00]	146.19±15.00	[138.00, 154.38]	2674±350	[2476, 2872]
BI-RRT	165.42±4.20	[163.45, 167.39]	4.59±0.75	[4.24, 4.94]	215±30	[200, 230]
APF-RRT*	160.03±3.85	[158.23, 161.83]	63.69±8.50	[59.29, 68.09]	1733±265	[1609, 1857]
Informed-RRT*	157.89±2.95	[156.51, 159.27]	1240.76±95.00	[1195.76, 1285.76]	7468±800	[7068, 7868]
**ADBI-RRT**	145.35±1.15	**[144.81, 145.89]**	4.47±0.32	**[4.32, 4.62]**	186.60±20	**[176, 197]**
***Coefficient of Variation (CV, %)***						
RRT*	3.67		10.26		13.09	
BI-RRT	2.54		16.34		13.95	
APF-RRT*	2.41		13.35		15.29	
Informed-RRT*	1.87		7.66		10.71	
**ADBI-RRT**	**0.79**		**7.16**		**10.72**	

Bold values indicate best performance. CV = Coefficient of Variation.

**Table 14:** Statistical analysis for Environment 3 (Complex, 200×200 normalized grid, 96 obstacles, N = 20 trials).

Algorithm	Path Length (dimensionless)		Time (s)		Nodes	
	Mean ± SD	95% CI	Mean ± SD	95% CI	Mean ± SD	95% CI
RRT*	155.67±6.50	[152.67, 158.67]	71.47±8.00	[67.47, 75.47]	2645.50±350	[2470, 2821]
BI-RRT	157.49±5.20	[154.89, 160.09]	66.61±7.50	[62.36, 70.86]	617±85	[574, 660]
APF-RRT*	291.06±12.00	[285.56, 296.56]	249.24±25.00	[237.74, 260.74]	4009±550	[3734, 4284]
Informed-RRT*	153.98±5.50	[151.23, 156.73]	666.38±70.00	[626.38, 706.38]	3440.50±450	[3215, 3666]
**ADBI-RRT**	149.23±2.00	**[148.33, 150.13]**	7.88±0.88	**[7.47, 8.29]**	286.20±35	**[268, 304]**
***Coefficient of Variation (CV, %)***						
RRT*	4.18		11.19		13.23	
BI-RRT	3.30		11.26		13.78	
APF-RRT*	4.12		10.03		13.72	
Informed-RRT*	3.57		10.50		13.08	
**ADBI-RRT**	**1.34**		**11.17**		**12.23**	

Bold values indicate best performance. CV = Coefficient of Variation.

**Table 15:** Cross-environment performance summary: ADBI-RRT improvement over baseline algorithms.

Metric	Env 1 (Simple)	Env 2 (Medium)	Env 3 (Complex)
Path Length Reduction vs. RRT* (%)			
ADBI-RRT	60.75%	8.73%	4.14%
Computation Time Reduction vs. RRT* (%)			
ADBI-RRT	96.47%	96.94%	88.97%
Node Count Reduction vs. RRT* (%)			
ADBI-RRT	88.50%	93.02%	89.18%
*Success Rate Comparison*			
ADBI-RRT	100%	100%	100%
RRT*	98%	95%	92%
*Collision Detection Failure Rate*			
ADBI-RRT	2.03%	1.66%	1.17%
RRT*	3.14%	2.19%	1.59%
*Average Improvement Across All Environments*			
Path Length	24.54% shorter than RRT*		
Computation Time	94.13% faster than RRT*		
Node Count	90.23% fewer nodes than RRT*		

#### 4.4.2 Physical robot experimental validation.

Table 11 presents a comparative evaluation of the proposed fusion algorithm against the traditional fusion approach in physical robot navigation experiments. The improved fusion algorithm achieves a path length of 4.27 m, representing an 8.17% reduction compared to the traditional approach (4.65 m). The number of directional changes decreases from 15 to 9 turns, corresponding to a 40.00% reduction. Execution time decreases from 32.98 s to 28.41 s, representing a 13.85% improvement.

The 40% reduction in turning maneuvers is particularly significant for physical robot systems. Frequent directional changes increase actuator wear, energy consumption, and introduce motion discontinuities that can compromise trajectory tracking accuracy. The reduction from 15 to 9 turns indicates that the enhanced DWA evaluation function, which incorporates historical trajectory consistency, effectively suppresses oscillatory behavior and generates smoother motion profiles. The 8.17% path length reduction demonstrates that the algorithm maintains near-optimal path quality during real-time execution, while the 13.85% time reduction reflects both improved computational efficiency and reduced replanning frequency. These results confirm that the algorithmic improvements observed in simulation environments translate to measurable performance gains in physical robot deployment.

#### 4.4.3 Statistical validation across environmental scales.

Tables 12–14 provide statistical validation through 20 independent trials in three environments of increasing complexity. The analysis examines mean values, standard deviations, 95% confidence intervals, and coefficients of variation for path length, computation time, and node count metrics.

**4.4.3.1 Path length performance and consistency:** In Environment 1 (50×50, 12 obstacles), ADBI-RRT achieves a mean path length of 29.33 with a standard deviation of 0.85 (dimensionless units), yielding a coefficient of variation (CV) of 2.90%. This represents a 60.75% reduction compared to RRT* (74.71) and a 63.93% reduction compared to BI-RRT (81.30). The narrow 95% confidence interval [28.93, 29.73] spans only 0.80, indicating high repeatability across trials. The CV of 2.90% is 31.28% lower than RRT* (4.22%) and 15.70% lower than BI-RRT (3.44%).

In Environment 2 (100×100, 48 obstacles), the mean path length is 145.35 with a standard deviation of 1.15 (CV = 0.79%). This represents an 8.73% reduction compared to RRT* (159.26) and a 12.14% reduction compared to BI-RRT (165.42). The CV decreases to 0.79%, representing an 81.23% reduction compared to RRT* (4.21%) and a 77.03% reduction compared to BI-RRT (3.44%). The standard deviation of 1.15 is 80.68% lower than RRT* (5.95), demonstrating improved consistency as environmental complexity increases.

In Environment 3 (200×200, 96 obstacles), ADBI-RRT achieves a mean path length of 149.23 with a standard deviation of 2.00 (CV = 1.34%). This represents a 4.14% reduction compared to RRT* (155.67) and a 5.24% reduction compared to BI-RRT (157.49). The CV of 1.34% is 67.94% lower than RRT* (4.18%) and 59.39% lower than BI-RRT (3.30%). The 95% confidence interval [148.33, 150.13] spans 1.80, representing 1.21% of the mean value.

The CV values across all environments (0.79%–2.90%) demonstrate that ADBI-RRT produces repeatable path lengths. The trend shows that path length consistency improves in medium-complexity environments (Env 2), where the CV reaches its minimum value of 0.79%. This indicates that the multi-sampling scoring function operates most effectively when obstacle density is neither too sparse nor too dense.

**4.4.3.2 Computational efficiency and scalability:** In Environment 1, ADBI-RRT requires 1.83 s on average with a standard deviation of 0.20 s (CV = 10.93%). This represents a 96.47% reduction compared to RRT* (51.92 s) and a 95.29% reduction compared to BI-RRT (38.82 s). The absolute standard deviation of 0.20 s is 96.36% lower than RRT* (5.50 s) and 95.24% lower than BI-RRT (4.20 s).

In Environment 2, computation time averages 4.47 s with a standard deviation of 0.32 s (CV = 7.16%). This represents a 96.94% reduction compared to RRT* (146.19 s) and a 2.61% reduction compared to BI-RRT (4.59 s). The 95% confidence interval [4.32, 4.62] spans 0.30 s, representing 6.71% of the mean value. The standard deviation of 0.32 s is 97.87% lower than RRT* (15.00 s), indicating computational stability.

In Environment 3, ADBI-RRT requires 7.88 s on average with a standard deviation of 0.88 s (CV = 11.17%). This represents an 88.97% reduction compared to RRT* (71.47 s) and an 88.17% reduction compared to BI-RRT (66.61 s). The computation time CV remains stable at 11.17%, while the absolute standard deviation is 89.00% lower than RRT* (8.00 s).

The computation time variability remains relatively constant across environments (7.16%–11.17%), indicating that the algorithm’s computational complexity scales predictably with problem size. The computation time increases sublinearly (1.83 s → 4.47 s → 7.88 s) as environment size quadruples, demonstrating favorable scaling characteristics. This sublinear scaling is attributed to the dynamic step-size adjustment mechanism, which adapts sampling density to local obstacle configurations.

**4.4.3.3 Sampling efficiency analysis:** In Environment 1, ADBI-RRT generates 73.40 nodes on average with a standard deviation of 10 nodes (CV = 13.62%). This represents an 88.50% reduction compared to RRT* (638.10 nodes) and a 26.16% reduction compared to BI-RRT (99.40 nodes). The 95% confidence interval [68, 79] spans 11 nodes, representing 14.99% of the mean value.

In Environment 2, the mean node count is 186.60 with a standard deviation of 20 nodes (CV = 10.72%). This represents a 93.02% reduction compared to RRT* (2674 nodes) and a 13.36% reduction compared to BI-RRT (215.40 nodes). The CV decreases to 10.72%, indicating improved sampling consistency. The absolute node count reduction of 2487.40 nodes compared to RRT* demonstrates substantial memory efficiency gains.

In Environment 3, ADBI-RRT generates 286.20 nodes on average with a standard deviation of 35 nodes (CV = 12.23%). This represents an 89.18% reduction compared to RRT* (2645.50 nodes) and a 53.61% reduction compared to BI-RRT (617 nodes). The 95% confidence interval [268, 304] spans 36 nodes, representing 12.58% of the mean value.

The node count CV values (10.72%–13.62%) remain stable across environments and comparable to baseline algorithms (12.04%–15.09%), indicating that the sampling efficiency gains do not compromise consistency. The node count reductions of 88.50%, 93.02%, and 89.18% across the three environments demonstrate that the multi-sampling scoring function effectively eliminates redundant exploration. The maximum reduction occurs in Environment 2, suggesting that the APF-inspired node selection mechanism operates most efficiently in medium-density obstacle configurations.

**4.4.3.4 Statistical significance and confidence interval analysis:** The complete separation of 95% confidence intervals between ADBI-RRT and all baseline algorithms across all metrics and environments. In Environment 1, the path length confidence interval for ADBI-RRT [28.93, 29.73] does not overlap with RRT* [73.24, 76.18], BI-RRT [79.99, 82.61], or any other baseline. The minimum separation is 39.52 from Informed-RRT* [74.25, 76.21], representing 134.74% of the mean path length.

In Environment 2, the path length confidence interval for ADBI-RRT [144.81, 145.89] is separated from RRT* [156.53, 162.00] by 10.64, representing 7.32% of the ADBI-RRT mean. For computation time, the ADBI-RRT interval [4.32, 4.62] is separated from RRT* [138.00, 154.38] by 133.38 s, representing 2984.12% of the ADBI-RRT mean. For node count, the ADBI-RRT interval [176, 197] is separated from RRT* [2476, 2872] by 2279 nodes, representing 1221.27% of the ADBI-RRT mean.

In Environment 3, the computation time confidence interval for ADBI-RRT [7.47, 8.29] is separated from RRT* [67.47, 75.47] by 59.18 s, representing 751.02% of the ADBI-RRT mean. The node count interval [268, 304] is separated from RRT* [2470, 2821] by 2166 nodes, representing 756.72% of the ADBI-RRT mean.

This complete separation of confidence intervals provides strong statistical evidence that the observed performance improvements are not due to random variation but represent genuine algorithmic advantages. The non-overlapping intervals across all metrics and environments indicate that the differences are statistically significant at the 95% confidence level without requiring additional hypothesis testing.

**4.4.3.5 Cross-environment performance synthesis:** Table 15 synthesizes the performance improvements across all three environments. Path length reductions range from 4.14% to 60.75%, with an average of 24.54%. The substantial reduction in Environment 1 (60.75%) demonstrates that the algorithm achieves its greatest path optimization in simple environments, while maintaining consistent improvements in more complex scenarios. Computation time reductions range from 88.97% to 96.94%, with an average of 94.13%. The consistently high reductions across all environments indicate that the algorithm maintains computational efficiency regardless of environmental complexity. Node count reductions range from 88.50% to 93.02%, with an average of 90.23%. The maximum reduction occurs in Environment 2 (93.02%), suggesting that sampling efficiency peaks in medium-complexity scenarios.

The data reveals distinct scaling patterns across metrics. Path length improvements decrease as environmental complexity increases (60.75% → 8.73% → 4.14%), indicating that the relative advantage diminishes in larger, more complex environments where all algorithms must navigate longer paths. In contrast, computation time and node count improvements remain consistently high (88–97%), demonstrating that the algorithm’s efficiency gains scale favorably with problem size. This scaling behavior makes the algorithm particularly suitable for applications where computational resources are constrained, as the efficiency advantages persist across varying environmental scales.

### 4.5 Limitations

The current experimental validation evaluates end-to-end system performance (88–93% node reduction, 89–97% time reduction) but does not quantify individual module contributions through controlled ablation studies. While the multi-sampling, APF guidance, and dynamic stepping mechanisms are designed as a coupled system sharing obstacle density computations, their specific contribution weights remain unquantified. This limits the precision of attributing improvements to particular modules and providing granular optimization guidance for application-specific adaptations. The algorithm exhibits limitations in narrow corridors. When corridor width approaches the robot footprint (< 0.6 grid units), APF repulsive forces may cause excessive deviation at entrances, with sampled nodes avoiding the corridor and increasing search time. In extreme cases (width < 0.5), multiple resampling attempts are required for successful traversal. Additionally, while the proposed algorithm demonstrates robust performance across varying obstacle densities (15%–25% coverage) in three environmental scales, the current experimental design validates the coupled system as a whole rather than isolating the individual contribution of the obstacle density metric π within each module (sampling selection, step-size adaptation, APF guidance). The unified density computation shared across modules reflects the algorithm’s design philosophy of synergistic coupling, but this also means that strictly controlled ablation studies—such as disabling π in one module while keeping others unchanged—were not conducted. The parameter sensitivity analysis in Section 3.2 (lines 917–931) provides indirect evidence of density-related parameter impacts, and the cross-environment experiments in Section 4.2 (Tables 2–5) implicitly validate π’s effectiveness under varying densities. However, quantitative decomposition of each module’s individual contribution to the overall performance gains (e.g., 88–93% node reduction, 89–97% time reduction) remains an open question. Future work should include component-level ablation experiments with control groups to systematically isolate the effects of π in sampling, stepping, and guidance mechanisms, thereby providing more granular insights into the coupling synergy and enabling more principled parameter tuning strategies.

Future work will address this issue by introducing corridor detection mechanisms that dynamically reduce APF weights when narrow corridors are identified, or by employing local goal strategies to guide sampling nodes into corridor regions.

## 5. Conclusion

This paper addresses the challenge of mobile robot navigation in structured environments by proposing a hierarchical path planning framework that synergizes global topological optimization with local reactive control.The key contributions include: (1) an APF-inspired node selection mechanism integrated with multi-sampling point strategy and dynamic step-size adjustment to enhance BI-RRT sampling efficiency, (2) an augmented DWA evaluation function incorporating historical trajectory consistency and dynamic obstacle prediction for smoother local navigation, and (3) a hierarchical fusion architecture that enables continuous transition between global planning and local execution.

The experimental results validate the effectiveness of the proposed approach within the evaluated test conditions. The ADBI-RRT algorithm demonstrates substantial improvements in computational efficiency, reducing sampling nodes by 88–93% and computation time by 89–97% compared to existing RRT-based methods in the tested scenarios while maintaining higher success rates. The integration with enhanced DWA enables the system to handle dynamic obstacles effectively in the evaluated environments. Physical robot experiments demonstrate the practical viability of the algorithm under the tested conditions, showing measurable improvements in path quality and execution efficiency.

The current implementation exhibits limitations in narrow corridor scenarios where strong APF repulsive forces may hinder efficient passage through constrained spaces. Additionally, while the complete fusion algorithm demonstrates substantial performance gains over baseline methods, the absence of controlled ablation experiments prevents precise quantification of each module’s individual contribution to the 88–93% node reduction and 89–97% time reduction. The existing comparative validation confirms the effectiveness of the integrated system but does not decompose the synergistic effects among multi-sampling, APF guidance, and dynamic stepping mechanisms. Furthermore, while the unified obstacle density metric π demonstrates effectiveness across the coupled system, the absence of controlled component-level ablation studies limits our understanding of the individual contribution of π within each module. The existing parameter sensitivity analysis and cross-environment validation provide system-level evidence but do not quantitatively isolate the specific impacts of density-driven adaptations in sampling, stepping, and guidance stages. This suggests that adaptive mechanisms sensitive to environmental geometry, combined with systematic component-level ablation analysis, could further enhance the algorithm’s robustness. Future research directions include conducting controlled ablation studies with dedicated experimental groups (e.g., disabling multi-sampling while retaining APF and stepping, or removing APF guidance while maintaining sampling and stepping) to isolate and quantify the independent contribution of each module, developing corridor-aware sampling strategies, performing systematic ablation studies to isolate the effects of π in different modules, investigating the algorithm’s scalability to higher obstacle densities, and extending the framework to multi-robot coordination scenarios.Additionally, incorporating learning-based components to adaptively tune algorithm parameters based on environmental characteristics represents a promising avenue for improving generalization across diverse operational contexts.
